# Synthesis and Antibacterial Activity of New 6″-Modified Tobramycin Derivatives

**DOI:** 10.3390/antibiotics13121191

**Published:** 2024-12-06

**Authors:** Kseniya S. Shapovalova, Georgy V. Zatonsky, Elizaveta A. Razumova, Daria A. Ipatova, Dmitrii A. Lukianov, Petr V. Sergiev, Natalia E. Grammatikova, Alexander S. Tikhomirov, Andrey E. Shchekotikhin

**Affiliations:** 1 Gause Institute of New Antibiotics, 11 B. Pirogovskaya Street, Moscow 119021, Russia; schapowalowa.kseniya2014@yandex.ru (K.S.S.); gzatonsk@gmail.com (G.V.Z.); ngrammatikova@yandex.ru (N.E.G.); tikhomirov.chem@gmail.com (A.S.T.); 2 Department of Chemistry, Lomonosov Moscow State University, Leninskie Gory 1, Moscow 119991, Russia; elizaveta_razumova@list.ru (E.A.R.); ipatova.daria@yandex.ru (D.A.I.); dmitrii.a.lukianov@gmail.com (D.A.L.); petya@belozersky.msu.ru (P.V.S.); 3 Center for Molecular and Cellular Biology, Moscow 121205, Russia; 4 A.N. Belozersky Institute of Physico-Chemical Biology, Lomonosov Moscow State University, Moscow 119991, Russia

**Keywords:** antimicrobial resistance, aminoglycosides, tobramycin, chemical modification, semisynthetic antibiotics, mode of action, translation inhibition

## Abstract

**Objectives:** Aminoglycosides are one of the first classes of natural antibiotics which have not lost relevance due to their broad spectrum of action against Gram-positive, Gram-negative bacteria and mycobacteria. The high growth rate of antimicrobial resistance (AMR) together with the severe side effects of aminoglycosides increase the importance of developing improved semisynthetic derivatives. **Methods:** In this work, we proposed a synthetic route to new tobramycin derivatives modified at the 6″-position with aminoalkylamine or guanidinoalkylamine residues. **Results:** The antibacterial activity of the new compounds against reference strains of microorganisms was comparable to the parental tobramycin. In striking contrast to tobramycin (resistance index, >256), its 6″-modified derivatives were significantly more potent against resistant clinical isolates of *P. aeruginosa* strains (resistance index = 4–16) and they demonstrated a promising AMR circumvention in *E. coli* strains associated with mutations in the fusA gene encoding elongation factor G. All the obtained tobramycin derivatives exhibited reduced cytotoxicity for the eukaryotic HEK293T cells compared to the tobramycin and thereby they potentially may have improved therapeutic index. The proposed modification of the 6″-position of tobramycin does not change the mechanism of aminoglycoside’s antibacterial activity: new compounds induced translation errors which resulted in the inhibition of protein synthesis in bacterial cells. **Conclusions:** Taken together, we can suggest that further modifications of the 6″-position of tobramycin may be beneficial for circumvention of AMR to aminoglycosides or used for conjugation with other molecules of interest.

## 1. Introduction

Throughout the pressure of induced selection, pathogenic microorganisms have developed resistance to widely used antibiotics. This has been exacerbated by the misuse of antimicrobial agents, both in medical practice and in agriculture, leading to a significant increase in the level of antimicrobial resistance (AMR) [1]. The AMR-associated infections became the cause of approximately 5 million deaths in 2019, predominantly in low- and middle-income countries [2], instead of the initially estimated 1.2 million [3]. Thus, the O’Neill’s prognosis [3] of an increase up to 10 million deaths per year by 2050 is now considered even too optimistic. The World Health Organization’s 2024 report identifies a group of 15 priority antibiotic-resistant pathogens, most of which are multidrug-resistant Gram-negative bacteria [2]. This highlights the need of creating new and updated means of eradicating Gram-negative infections.

**Figure 1:**
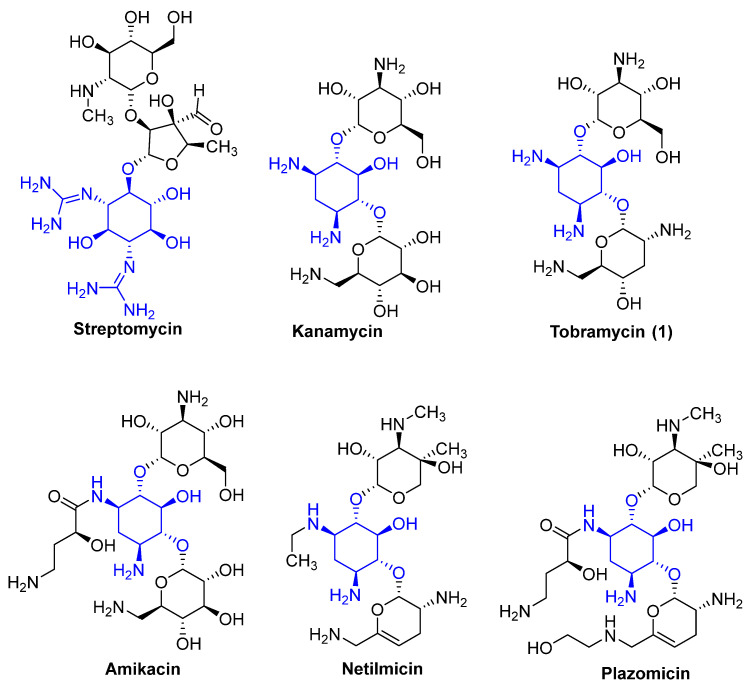
Structures of selected natural (**top**) and semi-synthetic (**bottom**) aminoglycoside antibiotics.

Aminoglycosides are one of the first discovered classes of natural antibiotics which still remain clinically relevant due to their broad spectrum of action including Gram-positive (G+), Gram-negative bacteria (G−) and mycobacteria. In particular, streptomycin and kanamycin (Figure 1) are applied in a combination therapy as the second line treatment of *Mycobacterium tuberculosis* [4]. Tobramycin is used for the curing of eye infections and respiratory tract infections caused by *Pseudomonas aeruginosa* (Figure 1) [5,6]. The widespread application of aminoglycosides since the 1940s stimulated the emergence of bacterial resistance. Some of the specific modes of resistance to aminoglycosides are being implemented with various modifying enzymes such as acetyltransferase (AAC), phosphotransferase (APH) or nucleotidyl transferase (ANT), etc. [7]. However, in recent years, new mechanisms of resistance to this class of antibiotics have been discovered, for example, mutations in the EF-G gene in *P. aeruginosa* [8]. It is worth noting that such kind of resistance was detected in clinical isolates of *P. aeruginosa* obtained after treatment with tobramycin [6]. Altogether, the sustained growth of AMR towards aminoglycosides together with their serious side effects, including oto- and nephrotoxicity [9,10], emphasize the importance of developing of new aminoglycoside derivatives.

In response to AMR spreading, both the pharmaceutical industry and academia have taken efforts to combat this challenge. Among the other strategies for AMR circumvention, the chemical modification of natural antibiotics has a validated approach in the development of antibacterial drugs. Semi-synthetic derivatives of aminoglycosides have significant advantages over parental antibiotics, including improved activity towards resistant strains of pathogens. In particular, such antibacterial agents as amikacin, netilmicin and plazomicin (Figure 1) [11,12] were approved for treatment of serious bacterial infections, including sepsis, complicated urinary tract infections, and nosocomial respiratory tract infections. Of note, plazomicin is listed as a «drug of choice» which should be used only to combat severe infections caused by multidrug-resistant pathogens.

**Figure 2:**
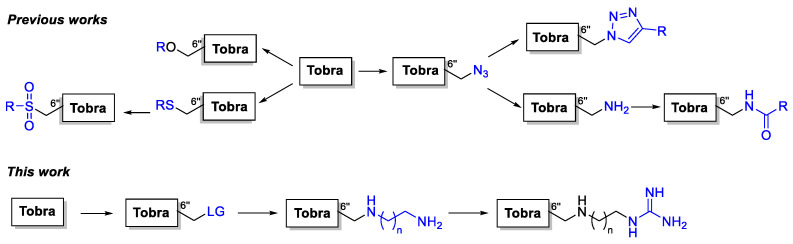
The key directions of chemical transformation of the 6″-hydroxy group of tobramycin (Tobra).

Tobramycin was chosen for further functional group modifications because of its high effectiveness against Gram-negative bacteria, such as *P. aeruginosa*. These bacteria are currently difficult to treat due to the presence of various types of AMR genes, including specific resistance to aminoglycosides. Extensive reviews have been recently published on various types of aminoglycosides modifications [13,14]. Considering the presence of several amino and hydroxyl groups with similar reactivity in its structure and the importance of some of them for ribosomal binding, we chose the primary hydroxyl group at the 6″-position of the antibiotic for modification, since it is more accessible for selective transformations. Previously, 6″-hydroxyl group of tobramycin underwent several transformations, including primary amine, azide, triazole, as well as derivatives of amides, ethers, thioethers, and sulfones (Figure 2) [14,15,16,17,18,19,20]. It has been demonstrated that attaching a linear alkyl chain (e.g., dodecyl or tetradecyl radical) at the 6″-position of tobramycin or kanamycin B via a thioether bond leads to aminoglycosides with antifungal activity against *C. albicans* [21,22]. To enhance the activity of the tobramycin derivative containing a dodecyl radical at the 6″-position, researchers explored how different functional groups acting as linkers between the alkyl chain and the AG backbone affected the efficacy of derivatives against 22 fungal strains. The resulting semi-synthetic tobramycin derivatives had antifungal potency against *C. albicans* and were active against clinical isolates of *C. neoformans* [23].

Introducing additional aminoalkyl or guanidinoalkyl groups into the structure of natural antibiotics often enhances their interaction with cellular targets and helps overcome antimicrobial resistance. It also increases aqueous solubility and optimizes pharmacokinetic profiles, among other benefits [24,25,26,27,28]. In particular, a selective modification of the 6″-hydroxyl group of kanamycin A increased the antibacterial activity compared to the original antibiotic [29]. To tackle AMR with aminoglycoside antibiotics, we assessed the feasibility of introducing aminoalkylamino and guanidinoalkylamino groups at the 6″-position of tobramycin. Here, we describe a method for synthesizing new 6″-modified tobramycin derivatives and evaluating their antibacterial properties. This includes studies against resistant strains of microorganisms with characterized mutations in the fusA (EF-G) gene, as well as assessments of the cytotoxicity and mechanisms of action.

## 2. Results and Discussion

### 2.1. Chemistry

**Scheme 1:**
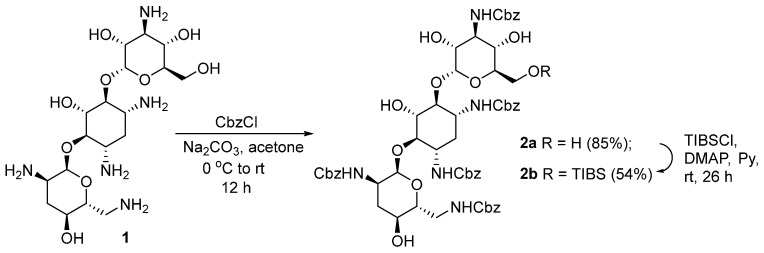
Synthesis of 1,3,6′,2′,3″-(penta-*N*-Cbz)-6″-*O*-(2,4,6-triisopropylbenzosulfonyl)tobramycin (**2b**).

To synthesize new 6″-modified tobramycin derivatives, we employed a strategy consisting of four synthetic steps: (1) exhaustive protection of all amino groups; (2) activation of the primary 6″-hydroxyl group by transformation into a good leaving group (LG); (3) nucleophilic substitution of the activated 6″-LG; and (4) cleavage of *N*-protective groups. The preparation of the 6″-substituted tobramycin derivatives began from the commercially available tobramycin monosulfate (**1**), which was treated with benzyl chloroformate (CbzCl) in the presence of Na_2_CO_3_ resulting in 1,3,6′,2′,3″-(penta-*N*-Cbz)-tobramycin (**2a**, Scheme 1). Previously, the use of the sterically hindered sulfonating agent as 2,4,6-triisopropylbenzosulfonyl chloride (TIBSCl) was demonstrated which directs the reaction mainly at the primarily hydroxyl group at the 6″-position of kanamycin [30]. Sulfonylation of **2a** at the 6″-hydroxyl group with TIBSCl in the presence of DMAP in dry pyridine at room temperature resulted in 1,3,6′,2′,3″-(penta-*N*-Cbz)-6″-*O*-(2,4,6-triisopropylbenzosulfonyl)tobramycin (**2b**) in moderate yield.

**Scheme 2:**
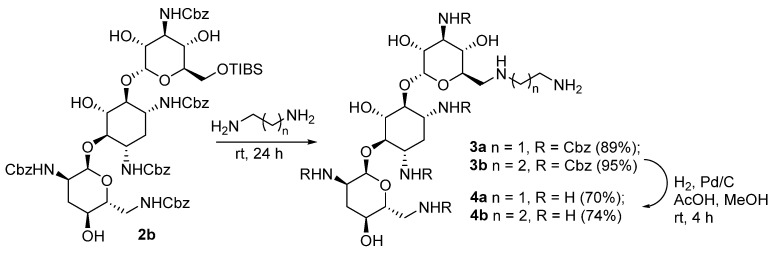
Synthesis of 6″-(aminoalkylamino)-6″-deoxytobramycin derivatives **4a**,**b.**

Introducing ethylenediamine and its homologs is considered a promising structural modification of natural products, which can enhance the pharmacological activity and solubility of compounds [31]. Substitution of the 6″-(2,4,6-triisopropylbenzosulfonyloxy) group of **2b** with an excess of 1,2-diaminoethane or 1,3-diaminopropane led to 6″-(aminoalkylamino)-1,3,6′,2′,3″-(penta-*N*-Cbz)-6″-deoxy-tobramycins **3a**,**b** (Scheme 2). Hydrogenolysis of *N*-Cbz-protected tobramycin derivatives **3a**,**b** on Pd on charcoal (5 mas. %) in the presence of acetic acid in methanol led to the cleavage of Cbz-groups and the formation of 6″-(aminoalkylamino)-6″-deoxytobramycins **4a**,**b** isolated as acetates in good yields.

**Scheme 3:**
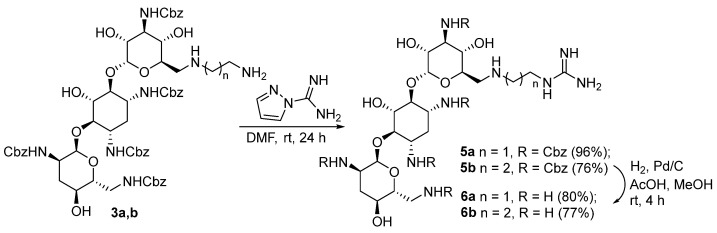
Synthesis of 6″-(guanidinoalkylamino)-6″-deoxytobramycin derivatives **6a**,**b**.

To enhance the basic character of the introduced 6″-side chains of 6″-(aminoalkylamino)-1,3,6′,2′,3″-(penta-*N*-Cbz)-6″-deoxy-tobramycin derivatives **3a**,**b**, the terminal amino group was converted into guanidine (Scheme 3). Treatment of **3a**,**b** with 1*H*-pyrazole-1-carboxamidine gave guanidinated Cbz-protected intermediates **5a**,**b**. Following deprotection of **5a**,**b** by hydrogenolysis gave the corresponding 6″-(2-guanidinoethylamino)-6″-deoxytobramycin (**6a**) and 6″-(3-guanidinopropyl-1-amino)-6″-deoxytobramycin (**6b**) which were also isolated as acetates in 77% and 59% yields (from **3a**,**b**), respectively.

The structure of all the obtained compounds was confirmed by physicochemical and spectral methods, including ^1^H and ^13^C NMR spectroscopy (Supporting Information, Figures S1–S20) and high-resolution mass spectrometry (Supporting Information, Figures S21–S30). The purity of the obtained compounds absorbing in the UV spectrum was confirmed by HPLC (Supporting Information, Figures S31–S36), as well as TLC and elemental analysis for non-absorbing compounds. Assignment of the signals in the ^1^H and ^13^C spectra is presented in Tables S1–S3, Supporting Information.

### 2.2. In Vitro Antibacterial Activity Studies

Antibacterial activity of the newly obtained tobramycin derivatives **4a**,**b**, **6a**,**b** was evaluated on the panel of G− and G+ bacterial strains, including *S. aureus* ATCC 29213 (G+), *E. coli* ATCC 25922 (G−), *E. coli* JW5503 (G−), *P. aeruginosa* ATCC 27853 (G−), and *M. smegmatis* ATCC 607, compared to tobramycin, kanamycin A and 6″-(2-aminoethylamino)-6″-deoxykanamycin (**7**), reported previously [29]. The results in Table 1 show that the introduction of the ethylenediamine side chain in the 6″-position of tobramycin slightly attenuated the antibacterial effect of the parental antibiotic against all the tested strains. Elongation of the alkyl linker from C2 to C3 was accompanied with a more pronounced decrease in potency. In contrast to kanamycin A and its derivative (**7**), all new derivatives **4a**,**b** and **6a**,**b**, as well as tobramycin (**1**) effectively suppress the growth of *P. aeruginosa* ATCC 27853 (MICs~0.5–8 µg/mL vs. MICs > 32 µg/mL for kanamycin and compound **7**). Guanidination of the terminal amine group of **4a** does not affect the antibacterial potency, while (2-guanidinoethyl)amino derivative **6a** superiors the action of (3-guanidinopropyl)amino substituted compound **6b**.

Subsequently, an in-depth assessment of the antibacterial activity of compounds **4a**,**b**, **6a**,**b** was carried out against clinical isolates of *P. aeruginosa* resistant to aminoglycosides, including tobramycin (Table 2). Importantly, 6″-aminoethylamino and 6″-guanidinoethylamino substituted derivatives **4a** and **6a** reliably and more potently inhibited the growth of the multidrug-resistant clinical isolates of *P. aeruginosa* 21653 and *P. aeruginosa* 21571 (MIC ~16–32 μg/mL) over the parental tobramycin (MIC > 128 μg/mL). Despite the mechanism of resistance of these strains of *P. aeruginosa* not being defined, the results prove that modification of the 6″-position of tobramycin may result in circumvention of AMR.

The measurements of the MIC values show that new derivatives have activity against susceptible strain of *E. coli* JW5503 kanS close to the original tobramycin (two-fold difference can be considered insignificant for this test). Table 3 again exhibits that derivatives **4a** and **6a**, which have a shorter side linker at the 6″-position than **4b** and **6b,** were more potent. We also performed comparative evaluation of new derivatives and parental tobramycin on *E. coli* strains (JW5503 kanS EF-G P610T and JW5503 kanS EF-G P610L) with mutations in the *fusA* gene encoding elongation factor G, which determined a resistance to kanamycin [32,33]. It was found that these mutations confer a resistance to all aminoglycosides of the 2-deoxystreptamine group (Supporting Information, Figure S37). Susceptible strain *E. coli* JW5503 kanS and its resistant mutants P610T and P610L were slightly less sensitive for novel tobramycin derivatives **4a**,**b**, **6a**,**b**, than the paternal antibiotic, as shown by the MIC values (Table 3). The activity of the derivatives **4a** and **6a**, which have a shorter linker group in the side chain, was slightly higher than **4b** and **6b** that correlated with the results (Table 1 and Table 2) of the screening of other stains of pathogens. Despite the fact that the new derivatives, **4a**,**b** and **6a**,**b**, were generally less potent than tobramycin, the ratios of the MIC values against resistant strains over the wild type strains (MIC_RS_/MIC_WTS_) were equal to the parent antibiotic and substantially superior kanamycin (Table 3).

### 2.3. In Vitro Cytotoxicity Studies

Despite the high effectiveness of aminoglycosides for the treatment of life-threatening infections, their use might be associated with acute adverse effects [35]. Tobramycin is known to have a certain level of toxicity. Therefore, it was crucial to evaluate the cytotoxicity of our analogs, which demonstrated activity against bacteria that was either slightly inferior to or comparable to that of the parent compound. The cytotoxicity of new compounds was assessed using the MTT assay [36] on a normal human epithelial-like cell culture (HEK293T). Importantly, derivatives **4a**,**b**, **6a**,**b** did not cause significant cell death in up to maximally used concentrations (IC_50_ > 140 µg/mL). In striking contrast, tobramycin, used as a control, showed cytotoxic effect with IC_50_ = 58 ± 3 µg/mL, indicating that performed modification of tobramycin at 6″-position potentially may reduce toxicity (Table 4, Supporting Information, Figure S38).

### 2.4. Cell-Free Translation Inhibition Assay

**Figure 3:**
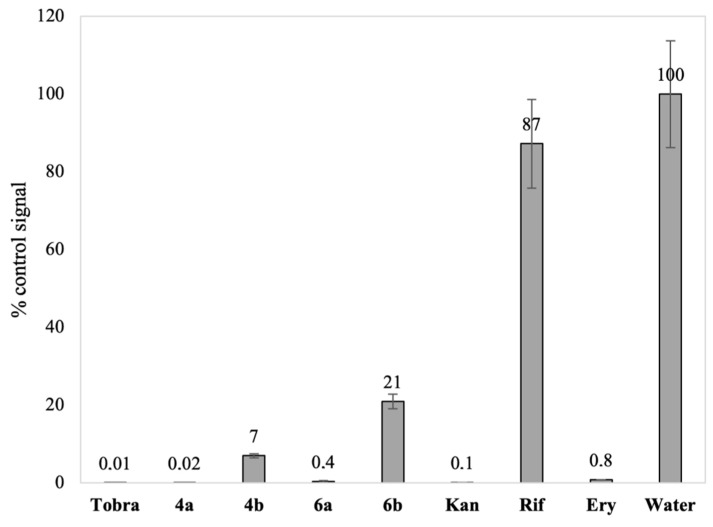
The inhibition of protein synthesis in vitro in the presence of new tobramycin derivatives **4a**,**b** and **6a**,**b** and reference drugs (Tobra—tobramycin, Kan—kanamycin A, Rif—rifampicin, Ery—erythromycin). All compounds were tested at a final concentration of 2 μM. Relative maximal rates of the firefly luciferase (Fluc) accumulation in vitro are shown. Experiments were performed at least three times, and the error bars represent the SD.

In order to determine whether the antibacterial action of new tobramycin derivatives was caused by the antibiotic’s translation-affecting mechanism or other processes, we tested their effect in the cell-free translation system. Figure 3 demonstrates that all tested derivatives, **4a**,**b**, **6a**,**b**, inhibit translation in vitro as well as the parent tobramycin. The efficacies **4a** and **6a** were comparable with the parent antibiotic while derivatives **4b** and **6b** with the elongated side chain linker were one order less potent. The antibiotic’s ability to bind to the 16S subunit of the ribosome is probably hindered by the bulkier substituent. The observed patterns of in vitro protein synthesis inhibition by tobramycin derivatives are in agreement with antibacterial properties of the compounds (Table 1, Table 2 and Table 3). Furthermore, the data indicate that the reduction in the efficacy of tobramycin derivatives is associated with their impact on the translation apparatus, rather than with the inhibition of cell penetration.

### 2.5. Mechanism of Action

**Figure 4:**
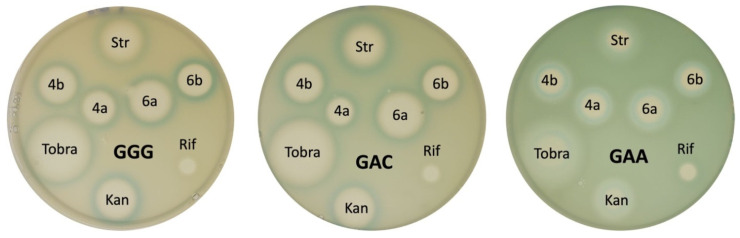
Tobramycin and new derivatives **4a**,**b** and **6a**,**b** increase the misreading of the 537 codon GGG/GAC/GAA of the reporter β-galactosidase gene in *E. coli*. Results of the test for compounds **4a**,**b**, **6a**,**b**, Str—streptomycin, Tobra—tobramycin, Kan—kanamycin A, and Rif—rifampicin. Concentration of **4a**,**b**, **6a**,**b** and tobramycin—50 mg/mL; streptomycin—5 mg/mL; kanamycin—5 mg/mL; and rifampicin—1 mg/mL. Volume of each antibiotic—2 µL.

It is known that tobramycin inhibits protein biosynthesis by the blocking of initiation and inducing translation errors [37]. We have tested whether the new tobramycin derivatives **4a**,**b** and **6a**,**b** retain the ability to induce translation errors. This assay uses an *E. coli* strain with a plasmid construct that encodes beta-galactosidase in such a way that a functional protein is only synthesized in the presence of translation errors. If the mechanism of action of the antibiotic is related to the translation errors, we observe indigo blue staining along the edge of the inhibition zone because functional b-galactosidase degrades the substrate X-Gal. As a result, if the antibiotic causes translation errors, blue staining forms around the edge of the inhibition zone. Kanamycin A and streptomycin acted as miscoding inductors while rifampicin affects RNA synthesis and does not induce ribosomal errors. The data in Figure 4 show that the tested compounds **4a**,**b** and **6a**,**b** caused a blue coloring of the bacterial lawn the same way as the original antibiotic, which induced the translation errors in the targeted cells. According to this, an introduction at the 6″-position of the aminoalkylamino or guanidinoalkylamino residues of tobramycin retains an original mechanism of aminoglycoside’s antibacterial activity affecting the protein synthesis.

## 3. Materials and Methods

### 3.1. Chemistry

Instruments, general information and synthetic procedures

Tobramycin sulfate was obtained from Abcr GmbH (Karlsruhe, Germany). Unless otherwise stated, all solvents and reagents were sourced from commercial suppliers such as Merck, ABCR Chemicals, and others, and were used without further purification. Pyridine was dried over KOH, then over CaCl_2_, and distilled prior to use. ^1^H and ^13^C NMR spectra were recorded in DMSO-*d*_6_ or D_2_O solutions at 25 °C on a Bruker AV III 500 MHz spectrometer (BrukerDaltonics GmbH, Bremen, Germany), with proton and carbon frequencies at 500.2 MHz and 125.8 MHz, respectively. Signal assignments in the NMR spectra were based on 2D COSY, TOCSY, NOESY, HSQC, and HMBC experiments, employing standard pulse sequences. The chemical shifts were referenced to the residual solvent signals in DMSO-*d*_6_ (2.50 ppm for ^1^H and 49.5 ppm for ^13^C) or to the internal DSS reference for D_2_O solutions. High-resolution mass spectrometry (HRMS) was performed using electrospray ionization (ESI) on a Bruker Daltonics microOTOF-QII instrument (BrukerDaltonics GmbH, Bremen, Germany). Elemental analysis was performed on the automated PerkinElmer 2400 CHN microanalyzer. Thin-layer chromatography (TLC) was conducted on 60F254 silica gel plates (Merck, Darmstadt, Germany), and column chromatography was carried out using Merck 60 silica gel. Tobramycin and its derivatives were visualized on TLC plates by exposure to iodine vapor or 6N sulfuric acid solution, followed by heating. UV-absorbing derivatives were also detected under UV light. All solutions were dried over sodium sulfate and concentrated under reduced pressure at temperatures below 40 °C. Analytical HPLC was performed on an LC-20AD chromatograph (Shimadzu Corp., Kyoto, Japan), equipped with a UV detector and a Kromasil-100-C18, 4.6 × 250 mm column (Akzo Nobel, Bohus, Sweden), at a flow rate of 1 mL/min using the following mobile phase systems: System A1: A (0.2% HCOONH_4_, pH 4.5) and B (MeCN), with a linear gradient of acetonitrile from 50% to 90% over 30 min; System A2: A (H_2_O) and B (MeCN), with a linear gradient of acetonitrile from 80% to 95% over 30 min; and System A3: A (0.2% HCOONH_4_, pH 4.5) and B (MeCN), with a linear gradient of acetonitrile from 20% to 90% over 30 min. The purity of intermediates and final compounds was determined to be at least 90% by HPLC for UV-absorbing compounds.

1,3,6′,2′,3″-(Penta-*N*-Cbz)-tobramycin **(2a).** The solution of benzylchloroformate (2.59 mL, 3.10 g, 18.16 mmol) in acetone (12 mL) was added dropwise at 0°C to the mixture of tobramycin monosulfate (**1**, 1.40 g, 3.00 mmol) and saturated water solution of Na_2_CO_3_ (16 mL). The reaction mixture was stirred at 0°C for 2 h and then at room temperature for 12 h. The resulting precipitate was filtered off, suspended in HCl (70 mL, 1 M) and stirred for 30 min. The solid was filtered off, washed with H_2_O (3 × 10 mL) and dried in vacuum over P_2_O_5_. The yield of compound **2a** is 2.9 g (85%) as a white solid, *R*_f_ = 0.5 (CHCl_3_:MeOH = 10:1), and HPLC (system A1) *t*_R_ = 15.7 min. ^1^H NMR (DMSO-*d*_6_; δ, ppm; *J*, Hz): 1.44–3.64 (m, 7H, cHex), 1.59–5.00 (m, 8H, CH_1′-6′_), 3.33–5.01 (m, 7H, CH_1″-6″_), 4.86–5.11 (m, 10H, -OCH_2_), 6.96 (s, 1H, -NH-CH_2 CH2′_-), 6.91 (s, 1H, -NH-CH_2 CH6′_-), 7.03 (s, 1H, -NH-CH_2 CH3″_-), 7.36 (s, 1H, -NH-CH_cHex3_-), 7.26–7.38 (m, 25H, 5×Ph), 7.17 (s, 1H, -NH-CH_cHex1_-). ^13^C NMR (DMSO-*d*_6_; δ, ppm): 33.1, 34.4, 41.6, 49.6, 49.9, 49.9, 56.4, 60.1, 64.6, 65.1, 65.2, 67.0, 69.8, 71.7, 73.2, 73.9, 81.6, 81.7, 97.1, 97.1, 127.6, 128.2, 137.0, 137.2, 155.4, 155.6, 155.8, 156.4, 156.5. HRMS (ESI): calculated for [C_58_H_68_N_5_O_19_]^+^ = 1138.4530, found [M+H]^+^ = 1138.4543.

1,3,6′,2′,3″-(Penta-*N*-Cbz)-6″-*O*-(2,4,6-triisopropylbenzosulfonyl)tobramycin **(2b)**. 2,4,6-Triisopropylbenzosulfonylchloride (173 mg, 0.57 mmol) and 4-(dimethylamino)pyridine (70 mg, 0.57 mmol) were subsequently added to the solution of 1,3,6′,2′,3″-(penta-*N*-Cbz)-tobramycin (**2a**, 150 mg, 0.13 mmol) in dry pyridine (3 mL). The reaction mixture was stirred 6 h at room temperature then, an additional amount of 2,4,6-triisopropylbenzosulfonylchloride (173 mg, 0.57 mmol) was added and the reaction mixture was stirred for 20 h. The reaction mixture was quenched by the addition of an aqueous solution of HCl (1N, 10 mL) and water (10 mL). The product was extracted with ethyl acetate (3 × 20 mL), organic layers were combined, dried over Na_2_SO_4_, and evaporated to dryness. The crude residue was purified by the column chromatography on silica gel with chloroform (100 mL) followed by a mixture (100 mL) of chloroform–methanol (50:1). The yield of compound **2b** is 100 mg, (54%) as a light-yellow solid. *R*_f_ = 0.5 (CHCl_3_:MeOH = 13:1), HPLC (system A2) *t*_R_ = 14.1 min. ^1^H NMR (DMSO-*d*_6_; δ, ppm; *J*, Hz): 1.18–1.19 (m, 18H, 6×-CH_3_), 1.40–3.59 (m, 7H, cHex), 1.56–4.99 (m, 8H, CH_1′-6′_), 2.92–4.04 (m, 3H, -CH(CH_3_)_3_), 3.35–5.02 (m, 7H, CH_1″-6″_), 4.95–5.21 (m, 10H, -OCH_2_), 6.89 (s, 1H, -NH-CH_2 CH2′_-), 6.96 (s, 1H, -NH-CH_2 CH6′_-), 7.07 (s, 1H, -NH-CH_2 CH3″_-), 7.17 (s, 1H, -NH-CH_cHex1_-), 7.26–7.38 (m, 25H, 5×Ph), 7.36 (s, 1H, -NH-CH_cHex3_-). ^13^C NMR (DMSO-*d*_6_; δ, ppm): 23.2, 23.4, 29.0, 33.1, 33.3, 34.6, 41.5, 49.6, 49.9, 50.0, 56.4, 64.5, 65.1, 65.2, 66.4, 67.4, 69.5, 69.8, 71.6, 73.8, 80.8, 81.7, 96.8, 97.1, 123.7, 127.4, 127.6, 128.2, 129.1, 137.0, 137.2, 150.1, 153.5, 155.3, 155.5, 156.5, 156.6. HRMS (ESI): calculated for [C_73_H_93_N_6_O_21_S]^+^ = 1421.6109, found [M+NH_4_]^+^ = 1421.5891

6″-(2-Aminoethyamino)-1,3,6′,2′,3″-(penta-*N-*Cbz)-6″-deoxytobramycin **(3a)**. The solution of the compound **2b** (44 mg, 0.03 mmol) in ethylenediamine (1.5 mL) was stirred at room temperature for 24 h. The reaction mixture was diluted with H_2_O (50 mL), the forming precipitate was filtered off, washed with H_2_O (3 × 10 mL) and dried in a vacuum at 45 °C and then in a vacuum over P_2_O_5_. The yield of compound **3a** is 32 mg (89%) as a light-pink solid. *R*_f_ = 0.35 (CHCl_3_:MeOH:HCOOH 13:5:0.1), HPLC (system A3) *t*_R_ = 20.3 min. ^1^H NMR (DMSO-*d*_6_; δ, ppm; *J*, Hz): 1.48–3.64 (m, 7H, cHex), 1.60–5.03 (m, 8H, CH_1′-6′_), 2.44 (s, 1H, -CH_1′_- CH_2′_-), 2.51–4.97 (m, 7H, CH″_1″-6″_), 2.52 (s, 1H, -CH_1′_- CH_2′_-), 4.86–5.10 (m, 10H, -OCH_2_), 6.66 (s, 1H, -NH-CH_2 CH3″_-), 6.91 (s, 1H, -NH-CH_2 CH6′_-), 7.00 (s, 1H, -NH-CH_2 CH2′_-), 7.18 (s, 1H, -NH-CH_cHex1_-), 7.26–7.38 (m, 25H, 5×Ph), 7.44 (s, 1H, -NH-CH_cHex3_-). ^13^C NMR (DMSO-*d*_6_; δ, ppm): 33.2, 34.2, 41.0, 41.7, 49.7, 49.8, 49.9, 50.0, 52.2, 56.5, 64.6, 65.1, 65.2, 69.9, 69.9, 71.4, 71.8, 73.9, 81.7, 82.1, 97.1, 97.1, 127.4, 127.5, 127.6, 128.2, 128.3, 137.0, 137.2, 155.4, 155.6, 155.8, 156.4, 156.5. HRMS (ESI): calculated for [C_60_H_75_N_7_O_18_]^+^ = 1181.5158, found [M+2H]^+^ = 1181.5524.

6″-((3-Aminoprop-1-yl)amino)-1,3,6’,2’,3″-(penta-*N*-Cbz)-6″-deoxytobramicyn **(3b)**. Compound **3b** was prepared from **2b** and propan-1,3-diamine as described for **3a**. White powder, yield 95%. *R*_f_ = 0.32 (CHCl_3_:MeOH:HCOOH 13:5:0.1), HPLC (system A3) *t*_R_ = 18.9 min. ^1^H NMR (DMSO-*d*_6_; δ, ppm; *J*, Hz): 1.40 (s, 1H, -CH_1′_-CH_2′_-), 1.47–3.66 (m, 7H, cHex), 1.60–5.02 (m, 8H, CH_1′-6′_), 2.44 (s, 1H, -CH_1′_-CH_2′_-), 2.50 (s, 1H, -CH_2′_-CH_3′_-), 2.51–4.97 (m, 7H, CH″_1″-6″_), 4.86–5.10 (m, 8H, -OCH_2_), 6.66 (s, 1H, -NH-CH_2 CH3″_-), 6.92 (s, 1H, -NH-CH_2 CH6′_-), 7.01 (s, 1H, -NH-CH_2 CH2′_-), 7.17 (s, 1H, -NH-CH_cHex1_-), 7.26–7.38 (m, 25H, 5×Ph), 7.44 (s, 1H, -NH-CH_cHex3_-). ^13^C NMR (DMSO-*d*_6_; δ, ppm): 33.0, 33.2, 34.2, 39.6, 41.6, 47.4, 49.6, 49.9, 50.0, 50.1, 56.5, 64.6, 64.9, 65.2, 69.8, 69.8, 71.2, 71.8, 73.8, 81.8, 82.1, 97.0, 97.2, 127.6, 128.2, 137.0, 155.4, 155.6, 155.8, 156.5. HRMS (ESI): calculated for [C_61_H_76_N_7_O_18_]^+^ = 1194.5242, found [M+H]^+^ = 1194.5330.

6″-(2-Aminoethyamino)-6″-deoxytobramycin acetate **(4a)**. 6″-(2-Aminoethyamino)-1,3,6′,2′,3″-(penta-*N-*Cbz)-6″-deoxytobramycin (**3a**, 60 mg, 0.05 mmol) was dissolved in methanol (2 mL) and Pd on carbon (5%, 60 mg) was added to the solution. Acetic acid was added to the mixture until pH = 3 was reached and the mixture was vigorously stirred at H_2_ flow (30 psi) for 4 h at room temperature. The catalyst was filtered off via celite pad; the cake was washed with methanol (5 mL) and H_2_O (5 mL), and combined filtrate was concentrated in a vacuum. The target compound was precipitated by acetone (10 mL), filtered off and dried in a vacuum over P_2_O_5._ The yield of compound **4a** was 30 mg (70%) as a white solid. *R*_f_ = 0.29 (NH_4_OH:*i*PrOH 10:7). ^1^H NMR (D_2_O; δ, ppm; *J*, Hz): 1.51–3.73 (m, 7H, cHex), 1.86–5.53 (m, 8H, CH_1′-6′_), 1.92 (s, 15H, -CH_3_-COOH), 2.86–5.10 (m, 7H, CH″_1″-6″_), 2.94 (s, 1H, -CH_1′_-CH_2′_-), 3.12 (s, 1H, -CH_1′_-CH_2′_-). ^13^C NMR (D_2_O; δ, ppm): 26.2, 35.2, 35.9, 41.1, 43.3, 49.0, 51.3, 51.5, 51.9, 53.4, 57.4, 68.4, 72.0, 72.5, 72.8, 73.8, 77.4, 85.5, 88.7, 99.1, 103.0, 184.3. HRMS (ESI): calculated for [C_20_H_44_N_7_O_8_]^+^ = 510.3173, found [M+H]^+^ = 510.3213. Anal. calculated for [C_20_H_43_N_7_O_8_×5AcOH×3H_2_O]: C, 41.71; H, 8.05; N, 11.35. Found: C, 41.95; H, 8.17; N, 11.12.

6″-(3-Aminopropyl-1-amino)-6″-deoxytobramycin acetate **(4b)**. Compound **4b** was prepared from **3b** as described for **4a**. White solid, yield 74%. *R*_f_ = 0.16 (NH_4_OH:*i*PrOH 10:7). ^1^H NMR (D_2_O; δ, ppm; *J*, Hz): 1.51–3.73 (m, 7H, cHex), 1.86–5.53 (m, 8H, CH_1′-6′_), 1.92 (s, 15H, -CH_3_- COOH), 2.86–5.10 (m, 7H, CH″_1″-6″_), 2.94 (s, 1H, -CH_1′_- CH_2′_-), 3.12 (s, 1H, -CH_1′_- CH_2′_-). ^13^C NMR (D_2_O; δ, ppm): 26.1, 35.2, 35.9, 41.1, 43.3, 49.0, 51.3, 51.5, 51.9, 53.4, 57.4, 68.4, 72.0, 72.5, 72.8, 73.8, 77.4, 85.5, 88.7, 99.1, 103.0, 184.3. HRMS (ESI): calculated for [C_21_H_46_N_7_O_8_]^+^ = 524.3533, found [M+H]^+^ = 524.3515. Anal. calculated for [C_21_H_45_N_7_O_8_×5AcOH×3H_2_O]: C, 42.41; H, 8.15; N, 11.17. Found: C, 42.77; H, 8.29; N, 10.98.

1,3,6′,2′,3″-(Penta-*N*-Cbz)-6″-(2-guanidinoethylamino)-6″-deoxytobramycin **(5a)**. Diisopropylethylamine (DIPEA, 93 µL, 0.53 mmol) was added to the mixture of 6″-(2-aminoethylamino)-1,3,6′,2′,3″-(penta-*N*-Cbz)-6″-deoxytobramycin (**3a**, 209 mg, 0.18 mmol) and 1*H*-pyrazol-1-carboximidamide hydrochloride (39 mg, 0.35 mmol) in DMF (2 mL). The reaction mixture was stirred at room temperature for 24 h, then H_2_O (50 mL) was added. The forming precipitate was filtered off, washed with H_2_O (3 × 10 mL), and dried in a vacuum over P_2_O_5._ The yield of compound **5a** is 201 mg (96%) as a white solid. *R*_f_ = 0.2 (CHCl_3_:MeOH:HCOOH = 5:1:0.3), HPLC (system A3) *t*_R_ = 20.2 min. ^1^H NMR (DMSO-*d*_6_; δ, ppm; *J*, Hz): 1.48–3.65 (m, 7H, cHex), 1.59–5.03 (m, 8H, CH_1′-6′_), 2.52 (s, 1H, -CH_1′_- CH_2′_-), 2.62 (s, 1H, -CH_1′_- CH_2′_-), 2.61–4.98 (m, 7H, CH″_1″-6″_), 3.09 (s, 2H, -CH_1′_- CH_2′_-), 4.86–5.10 (m, 10H, -OCH_2_), 6.56 (s, 1H, -NH-CH_2 CH3″_-), 6.87 (s, 1H, -NH-CH_2 CH6′_-), 6.97 (s, 1H, -NH-CH_2 CH2′_-), 7.15 (s, 1H, -NH-CH_cHex1_-), 7.26–7.38 (m, 25H, 5×Ph), 7.39 (s, 1H, -NH-CH_cHex3_-). ^13^C NMR (DMSO-*d*_6_; δ, ppm): 33.1, 34.4, 41.0, 41.6, 48.4, 49.5, 49.6, 49.9, 50.0, 56.3, 64.5, 65.0, 65.3, 69.1, 69.8, 71.4, 71.7, 73.2, 82.6, 97.0, 97.4, 80.9, 127.6, 128.3, 137.1, 155.4, 155.6, 155.8, 156.5, 157.3, 157.6, 155.7, 162.2. HRMS (ESI): calculated for [C_61_H_76_N_9_O_18_]^+^ = 1222.5303, found [M+H]^+^ = 1222.5284.

1,3,6′,2′,3″-(Penta-N-Cbz)-6″-(3-guanidinopropyl-1-amino)-6″-deoxytobramycin **(5b)**. Compound **5b** was prepared from **3b** as described for **5a**. White solid, yield 76%. *R*_f_ = 0.18 (CHCl_3_:MeOH:HCOOH = 5:1:0.3), HPLC (system A3) *t*_R_ = 19.3 min. ^1^H NMR (DMSO-*d*_6_; δ, ppm; *J*, Hz): 1.43–3.64 (m, 7H, cHex), 1.49 (s, 2H, -CH_1′_- CH_2′_-), 1.59–5.01 (m, 8H, CH_1′-6′_), 2.37 (s, 1H, -CH_1′_- CH_2′_-), 2.44 (s, 1H, -CH_1′_- CH_2′_-), 2.53–4.98 (m, 7H, CH_1″-6″_), 3.04 (s, 2H, -CH_2′_- CH_3′_-), 4.86–5.10 (m, 10H, -OCH_2_), 6.86 (s, 1H, -NH-CH_2 CH6′_-), 6.98 (s, 1H, -NH-CH_2 CH2′_-), 7.13 (s, 1H, -NH-CH_cHex1_-), 7.26–7.38 (m, 25H, 5×Ph). ^13^C NMR (DMSO-*d*_6_; δ, ppm): 28.9, 33.2, 34.5, 38.5, 41.6, 46.0, 49.6, 49.6, 49.9, 50.0, 56.4, 64.6, 65.0, 65.3, 69.3, 69.8, 71.2, 71.7, 73.2, 80.7, 81.7, 97.1, 97.3, 127.6, 128.3, 137.1, 155.4, 155.6, 155.7, 155.8, 156.5, 157.3, 157.6, 162.3. HRMS (ESI): calculated for [C_62_H_78_N_9_O_18_]^+^ = 1236.5460, found [M+H]^+^ = 1236.5483.

6″-(2-Guanidinoethylamino)-6″-deoxytobramycin acetate **(6a)**. Compound **6a** was prepared from **5a** as described for **4a**. White solid, yield 80%. *R*_f_ = 0.25 (NH_4_OH:*i*PrOH 10:7). ^1^H NMR (D_2_O; δ, ppm; *J*, Hz): 1.63–3.78 (m, 7H, cHex), 1.92 (s, 15H, -CH_3_- COOH), 1.96–5.67 (m, 8H, CH_1′-6′_), 3.04–5.14 (m, 7H, CH″_1″-6″_), 3.06 (s, 2H, -CH_1′_- CH_2′_-), 3.45 (s, 2H, -CH_1′_- CH_2′_-). ^13^C NMR (D_2_O; δ, ppm): 26.1, 33.5, 34.2, 42.1, 43.1, 49.8, 51.0, 51.3, 51.7, 53.3, 57.6, 67.9, 70.0, 71.5, 72.4, 73.1, 77.4, 84.1, 87.8, 97.6, 103.3, 159.9, 184.2. HRMS (ESI): calculated for [C_21_H_46_N_9_O_8_]^+^ = 552.3391, found [M+H]^+^ = 552.3418. Anal. calculated for [C_21_H_45_N_9_O_8_×5AcOH×1H_2_O]: C, 42.80; H, 7.76; N, 14.49. Found: C, 42.95; H, 7.91; N, 14.33.

6″-(3-Guanidinopropyl-1-amino)-6″-deoxytobramycin acetate **(6b)**. Compound **6b** was prepared from **5b** as described for **4a**. White solid, yield 77%. *R*_f_ = 0.18 (NH_4_OH:*i*PrOH 10:7). ^1^H NMR (D_2_O; δ, ppm; *J*, Hz): 1.61–3.76 (m, 7H, cHex), 1.92 (s, 15H, -CH_3_- COOH), 1.93–5.65 (m, 8H, CH_1′-6′_), 2.00 (s, 2H, -CH_1′_- CH_2′_-), 3.11 (s, 2H, -CH_1′_- CH_2′_-), 3.21–5.13 (m, 7H, CH_1″-6″_), 3.30 (s, 2H, -CH_2′_- CH_3′_-). ^13^C NMR (D_2_O; δ, ppm): 26.1, 27.9, 33.9, 34.6, 41.2, 43.1, 48.6, 51.0, 51.1, 51.7, 53.5, 57.4, 68.0, 71.0, 71.4, 71.6, 72.4, 77.5, 84.6, 87.9, 98.0, 103.2, 159.7, 184.2. HRMS (ESI): calculated for [C_22_H_47_N_9_O_8_]^+^ = 566.3548, found [M+H]^+^ = 566.3572. Anal. calculated for [C_22_H_47_N_9_O_8_×5AcOH×1H_2_O]: C, 43.48; H, 7.87; N, 14.26. Found: C, 43.72; H, 8.01; N, 14.03.

### 3.2. Microorganisms

The microbial strains used in this study were obtained from the collection of the Gause Institute of New Antibiotics. The control strains included in the analysis were *S. aureus* ATCC 29213, *E. coli* ATCC 25922, *E. coli* JW5503, *P. aeruginosa* ATCC 27853, *M. smegmatis* ATCC 607, and clinical isolates of *P. aeruginosa* resistant to tobramycin. The microbial cultures were stored at −75 °C in trypticase soy broth (Becton, Dickinson, France) containing 10–15% glycerol. Storage was carried out in accordance with CLSI guidelines [38]. Prior to experimentation, the bacterial strains were revived from cryopreservation by inoculating them onto trypticase soy agar (Becton, Dickinson, France) and incubating at (36 ± 1) °C for 16–24 h. Individual morphologically homogeneous colonies were then suspended in sterile physiological saline, and the turbidity of the suspension was adjusted to 0.5 McFarland units using a DEN-1 spectrophotometer (Biosan, Riga, Latvia), which corresponds to a concentration of 1.5 × 10^8^ CFU/mL.

Sample Preparation

The samples were dissolved in sterile distilled water or dimethyl sulfoxide (DMSO), depending on the physicochemical properties of the compounds under investigation, to a concentration of 10,000 µg/mL. Working solutions were then prepared by serial dilution, resulting in concentrations ranging from 64 µg/mL to 0.5 µg/mL.

Assay setting

Activity was evaluated by determining the minimum inhibitory concentration (MIC) values using the microdilution method in Mueller–Hinton Broth (Beckton and Dickinson, Le Pont-de-Claix, France), in accordance with the standard procedure for assessing the antimicrobial susceptibility of microorganisms [38].

The analysis was conducted in 96-well microtiter plates (Medpolymer, Saint Petersburg, Russia). The bacterial inoculum was introduced within 15 min of preparation. MIC values were determined after incubation for 15–18 h at (36 ± 1) °C. Microbial growth in the presence of the tested compounds was compared to the growth control (without exposure to the test samples). The MIC was defined as the lowest concentration at which visible microbial growth was inhibited.

To ensure the accuracy of the results, the control antibiotics kanamycin A and tobramycin (Abcr GmbH, Karlsruhe, Germany) were included, along with standard reference strains: *S. aureus* ATCC 29213 and *E. coli* ATCC 25922. The MIC values for kanamycin A were determined as follows: *S. aureus* ATCC 29213—1–4 µg/mL; *E. coli* ATCC 25922—1–4 µg/mL. For tobramycin, the MIC values were *S. aureus* ATCC 29213—0.12–1 µg/mL; *E. coli* ATCC 25922—0.25–1 µg/mL; and *P. aeruginosa* ATCC 27853—0.25–1 µg/mL. The MIC values of the reference strains were required to fall within the acceptable ranges specified in [30], provided that standard assay conditions were maintained.

### 3.3. Determination of the Minimum Inhibitory Concentration on E. coli Against Aminoglycoside-Resistant Mutants

The determination of the minimum inhibitory concentration (MIC) was performed on three strains from the working collection of MSU: *E. coli* JW5503 [34], which lacked a kanamycin resistance cassette removed as per the method outlined in [39], and two strains derived from this strain through in vivo selection with kanamycin. The latter strains harbored P610T and P610L substitutions in the *fusA* gene. For the MIC determination, 100 μL (rows 2–11) and 200 μL (row 1) of LB liquid medium containing a 1:1000 dilution of overnight culture was added to each well of a 96-well transparent plate. Subsequently, 2 μL of a 50 μg/mL antibiotic solution was introduced into the first row, and serial two-fold dilutions were performed across each horizontal row, decreasing the antibiotic concentration incrementally. The final two rows were designated as controls: row 12 contained LB medium without cells or antibiotics, and row 11 contained cells without antibiotics. Following 18 h of incubation at 37 °C with constant agitation, the optical density at 590 nm (A590) was measured using a Victor X5 2030 spectrofluorometer (PerkinElmer, Waltham, MA, USA). The MIC was defined as the antibiotic concentration in the first well where no cell growth occurred. Each experiment was conducted in triplicate for all tested antibiotics.

### 3.4. MTT Assay

An MTT assay was conducted to assess the cytotoxicity of compounds on HEK293T cell lines. Cell line HEK293T was kindly provided by Dr. E. Knyazhanskaya. A total of 2500 cells per well were seeded into 96-well plates containing 100 μL of DMEM/F12 medium (Paneco LLC, Moscow, Russia) and incubated at 37 °C with 5% CO_2_ for 24 h prior to treatment. Dilutions of the test compounds **4a**, **4b**, **6a**, and **6b**, prepared in deionized sterile water, were added in 11 μL aliquots to achieve final concentrations ranging from 8.75 to 140 μg/mL (five dilutions per compound) in triplicate. The cells were incubated with the compounds for 72 h, after which 0.5 mg/mL of MTT reagent (Paneco LLC, Russia) was added. Following a 1.5 h incubation, the medium was removed, and 120 μL of DMSO was added to dissolve the blue formazan derivative formed by metabolically active cells. The absorbance at 565 nm was measured using a plate reader, and the data were normalized to untreated control cells. The half-maximal inhibitory concentration (IC_50_) was calculated using GraphPad Prism 5 software (GraphPad Software, Inc., San Diego, CA, USA).

### 3.5. Cell-Free Translation Inhibition Assay

The inhibitory effects of the tested compounds on in vitro firefly luciferase synthesis were evaluated using the *E. coli* S30 Extract System for Linear Templates (Promega, Madison, WI, USA), as described previously [40]. Briefly, reaction mixtures with a total volume of 5 μL were prepared, containing 0.1 mM of a mixture of all canonical amino acids, 4 U of RiboLock RNase Inhibitor (Thermo Fisher Scientific, Waltham, MA, USA), and 2 μM of the tested compounds. After pre-incubation at room temperature for 5 min, 100 ng of in vitro-transcribed firefly luciferase mRNA was added. The mixtures were then incubated at 37 °C for 20 min. After incubation, each reaction was supplemented with 5 μL of Bright-Glo™ reagent (Promega, Madison, WI, USA), and chemiluminescence was measured using a VICTOR X5 Multilabel Plate Reader (PerkinElmer, Waltham, MA, USA). The luminescence values were normalized to the negative control (water), which was assigned 100% activity.

### 3.6. Determination of Translation Accuracy Using Reporters

To qualitatively evaluate translation accuracy in the presence of antibiotics, *E. coli* JW5503 [34] (kanamycin resistance cassette removed as per [39]) transformed with pJC27 plasmids was utilized. The plasmids encode the β-galactosidase gene containing a mutation in its active center (E537: GAA→GAC/GGG), alongside a control plasmid without the mutation. Active β-galactosidase synthesis could only occur if translation errors were introduced. Petri dishes (9 cm) containing 20 mL of solid LB agar (1.5%) supplemented with chloramphenicol (11.3 μg/mL) and X-Gal (80 μg/mL) were prepared. A secondary layer comprising 3.5 mL of LB agar (0.6%), supplemented with 11.3 μg/mL chloramphenicol and 0.5 mL of liquid culture (A600~1.0), was added and allowed to solidify. Antibiotics were applied to the plates in 1 μL aliquots at the following concentrations: 50 mg/mL for **4a**, **4b**, **6a**, **6b**, and tobramycin; 5 mg/mL for streptomycin and kanamycin; and 1 mg/mL for rifampicin. Plates were incubated at 37 °C for 18 h. The occurrence of blue indigo staining, a product of X-Gal substrate degradation by β-galactosidase, at the edge of inhibition zones indicated that the antibiotic induced translation errors. Results were documented using a Samsung Galaxy Tab A71 camera.

## 4. Conclusions

An emergence and rapid spread of AMR among pathogenic bacteria increases the urgency to develop more effective antibacterial agents. Aminoglycosides are still an important class of antibiotics for treating life-threatening infections. However, the rise of aminoglycoside-resistant strains of *P. aeruginosa*, *K. pneumoniae*, *A. baumannii*, and *M. tuberculosis*, reported in recent years, shows the relevance for developing updated aminoglycosides.

Here we developed the route to new tobramycin derivatives modified at the 6″-position with various *N*-substituted amino residues. An approach is based on the steric hindrance factor: the 6″-hydroxymethyl group can be selectively activated for nucleophilic substitution with the sulfonylation in contrast to secondary hydroxyl groups of the core. Following this method, four derivatives of natural antibiotic tobramycin bearing aminoalkylamines or guanidinoalkylamines at the 6″-position were prepared and evaluated.

The antibacterial activity of new compounds **4a**,**b** and **6a**,**b** was comparable to the parental tobramycin against control strains of microorganisms. In striking contrast to tobramycin (resistance index, >256), 6″-modified derivatives were significantly more potent against resistant clinical isolates of *P. aeruginosa* strains (resistance index = 4–16). We also demonstrated that new derivatives as well as tobramycin exhibit a promising AMR circumvention in *E. coli* strains associated with mutations in the *fusA* gene encoding elongation factor G, while another aminoglycoside antibiotic, for example, kanamycin A, was not as effective. Moreover, all of the tobramycin derivatives that were obtained had a tendency to have less cytotoxicity for eukaryotic HEK293T cells than tobramycin, so they could potentially have an improved therapeutic index.

The proposed modification of the 6″-position of tobramycin does not change the mechanism of aminoglycoside’s antibacterial activity: new compounds induced translation errors which resulted in the inhibition of protein synthesis in bacterial cells.

Taken together we can suggest that modifications of the 6″-position of tobramycin may be beneficial for circumvention of AMR to aminoglycosides. On the other hand, the 6″-position of tobramycin can be used for conjugation with other molecules (e.g., antibiotics of different classes or fluorescent probes) without substantial binding with cellular targets.

## Figures and Tables

**Table 1:** Antibacterial activity (MIC, µg/mL) of the new tobramycin derivatives **4a**,**b**, **6a**,**b** and reference drugs tobramycin, kanamycin A and its 6″-(2-aminoethylamino)-derivative **7**.

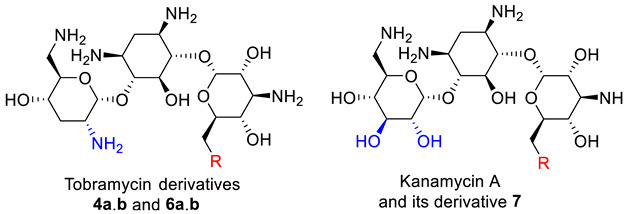						
Compound	R	MIC *, µg/mL				
		*S. aureus* ATCC 29213	*E. coli* ATCC 25922	*E. coli* JW5503	*P. aeruginosa* ATCC 27853	*M. smegmatis* ATCC 607
Tobramycin	OH	0.06	0.25	1	0.5	0.06
**4a**	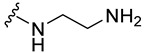	0.125	0.5	2	1	0.125
**4b**	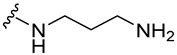	0.125	1	8	8	0.125
**6a**	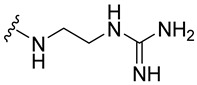	0.125	1	4	1	0.125
**6b**	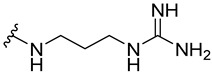	0.5	2	8	2	0.25
Kanamycin A	OH	0.5	1	1	>32	0.125
**7**	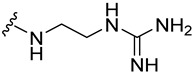	0.125	1	4	>32	0.06

* MIC—minimal inhibitory concentration, MICs are measured as the lowest concentration of agents that prevent any visible growth. The results of the experiments were definitely reproducible. In cases of full coincidence of the data obtained, the MIC is represented as a single number.

**Table 2:** Antibacterial activity (MIC, µg/mL) of new tobramycin derivatives **4a**,**b**, **6a**,**b**, kanamycin A derivative **7** and reference drugs tobramycin, ciprofloxacin and aztreonam against resistant clinical isolates of *P. aeruginosa*.

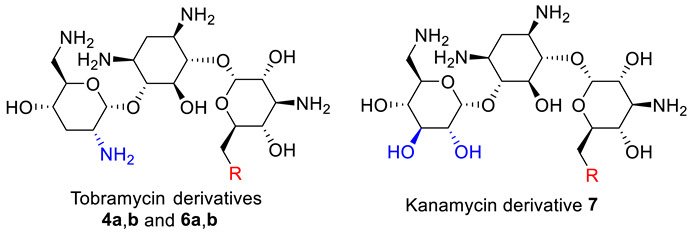							
Compound	R	MIC, µg/mL					
		*P. aeruginosa* Strains					
		ATCC 27853	27853	27853	27853	27853	27853
Tobramycin	OH	0.5	>128	>128	>128	>32	>32
Ciprofloxacin	-	0.125	32	64	32	32	32
Aztreonam	-	4	16	8	32	16	16
**4a**	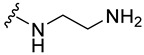	1	16	32	>32	>32	32
**4b**	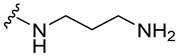	8	32	32	>32	>32	>32
**6a**	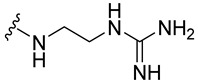	1	16	16	>32	32	32
**6b**	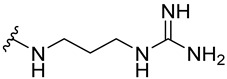	2	16	32	>32	>32	>32
**7**	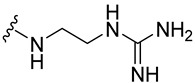	>128	>128	>128	>128	>128	>128

**Table 3:** Antibacterial activity against aminoglycoside-resistant strains (MIC, µg/mL) of new tobramycin derivatives **4a**,**b**, **6a**,**b** and reference drugs tobramycin, kanamycin A and control antibiotics.

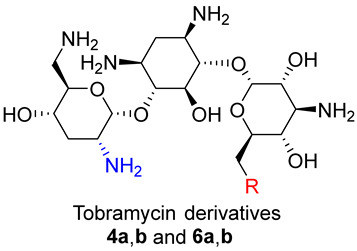				
Compound	R	MIC, µg/mL		
		*E. coli* JW5503 kanS [34]	*E. coli* JW5503 kanS EF-G P610T	*E. coli* JW5503 kanS EF-G P610L
Tobramycin	OH	5	20	40
**4a**	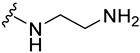	10	40	40
**4b**	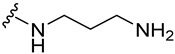	20	80	80
**6a**	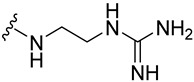	10	40	40
**6b**	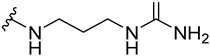	40	80	80
Kanamycin A	-	5	80	80
Rifampicin	-	5	5	5
Erythromycin	-	2	2	2

**Table 4:** Cytotoxicity (MTT test, 72 h) of new tobramycin derivatives **4a**,**b**, **6a**,**b**, and reference drug tobramycin for HEK293T cells.

Compound	IC_50_, µg/mL
**4a**	>140
**4b**	>140
**6a**	>140
**6b**	>140
Tobramycin	58 ± 3

## Data Availability

Data are contained within the article and in the Supporting Information file.

## Supplementary Materials

The following supporting information can be downloaded at: https://www.mdpi.com/article/10.3390/antibiotics13121191/s1, Tables S1–S3: Assignments of the signals in ^1^H and ^13^C NMR spectra of tobramycin derivatives; Figures S1–S20: ^1^H and ^13^C NMR spectra of the tobramycin derivatives; Figures S21–S30: HRMS (ESI) spectra of the tobramycin derivatives; Figures S31–S36: HPLC chromatograms of the tobramycin derivatives; Figure S37: Drop-test for E. coli strains with P610T/L substitution in EF-G; Figure S38: Cell viability curves of HEK293T cell line incubated with tested substances.
