# Supplementary material for: Synthesis and Antibacterial Activity of New 6″-Modified Tobramycin Derivatives
Source: Antibiotics (Basel). 2024 Dec 6;13(12):1191. doi: 10.3390/antibiotics13121191 (PMC11672562; doi:10.3390/antibiotics13121191)
Supplement: Supplementary file 1 [file antibiotics-13-01191-s001.zip › antibiotics-3312784-supplementary.pdf]

SUPPORTING INFORMATION

Synthesis and Antibacterial Activity of New 6″-Modified Tobramycin Derivatives

Kseniya S. Shapovalova [1], Georgy V. Zatonsky [1], Elizaveta A. Razumova [2], Daria A. Ipatova [2], Dmitrii A. Lukianov [2,3], Petr V. Sergiev [2,3,4], Natalia E. Grammatikova [1], Alexander S. Tikhomirov [1] and Andrey E. Shchekotikhin [1,*]

[1] Gause Institute of New Antibiotics, 11 B. Pirogovskaya Street, Moscow 119021, Russia
[2] Department of Chemistry, Lomonosov Moscow State University, Leninskie Gory 1, Moscow 119991, Russia
[3] Center for Molecular and Cellular Biology, Moscow 121205, Russia
[4] A.N. Belozersky Institute of Physico-Chemical Biology, Lomonosov Moscow State University, Moscow 119991, Russia
* Correspondence: shchekotikhin@mail.ru

Table of content

Table S1. ^{1}H and ^{13}C NMR data of tobramycin derivatives **2a-b**, **3a-3b** in DMSO-d_6. 4
Table S2. ^{1}H and ^{13}C NMR data of tobramycin derivatives **5a-b** in DMSO-d_6 5
Table S3. ^{1}H and ^{13}C NMR data of tobramycin derivatives **4a-b**, **6a-b** in D_2O................................6
Figure S1. ^{1}H NMR (500.2 MHz, DMSO-d_6) spectrum of 1,3,6',2′,3''-penta-*N*-Cbz-tobramycin **2a** 7
Figure S2. ^{13}C NMR (125.8 MHz, DMSO-d_6) spectrum of 1,3,6',2′,3''-penta-*N*-Cbz-tobramycin **2a**8
Figure S3. ^{1}H NMR (500.2 MHz, DMSO-d_6) spectrum of 1,3,6′,2′,3″-penta-N-Cbz-6″-*O*-(2,4,6-triisopropylbenzosulfonyl)tobramycin **2b** 9
Figure S4. ^{13}C NMR (125.8 MHz, DMSO-d_6) spectrum of of N-Cbz-6″-*O*-(2,4,6-triisopropylbenzosulfonyl)tobramycin **2b** 10
Figure S5. ^{1}H NMR (500.2 MHz, DMSO-d_6) spectrum of 6''-(2-aminoethyamino)-1,3,6',2′,3''-penta-*N*-Cbz-6''-deoxytobramycin **3a** 11
Figure S6. ^{13}C NMR (125.8 MHz, DMSO-d_6) spectrum of 6''-(2-aminoethyamino)-1,3,6',2′,3''-penta-*N*-Cbz-6''-deoxytobramycin **3a** 12
Figure S7. ^{1}H NMR (500.2 MHz, DMSO-d_6) spectrum of 6''-(3-aminopropyl-1-amino)-1,3,6',2′,3''-penta-*N*-Cbz-6''-deoxytobramycin **3b**..13
Figure S8. ^{13}C NMR (125.8 MHz, DMSO-d_6) spectrum of 6''-(3-aminopropyl-1-amino)-1,3,6',2′,3''-penta-*N*-Cbz-6''-deoxytobramycin **3b**..14
Figure S9. ^{1}H NMR (500.2 MHz, D_2O) spectrum of 6''-(2-aminoethyamino)-6''-deoxytobramycin **4a** 15
Figure S10. ^{13}C NMR (125.8 MHz, D_2O) spectrum of 6''-(2-aminoethyamino)-6''-deoxytobramycin **4a** 16
Figure S11. ^{1}H NMR (500.2 MHz, D_2O) spectrum of 6″-(3-Aminopropyl-1-amino)- 6″-deoxytobramycin **4b**..17
Figure S12. ^{13}C NMR (125.8 MHz, D_2O) spectrum of 6″-(3-Aminopropyl-1-amino)- 6″-deoxytobramycin **4b**..18
Figure S13. ^{1}H NMR (500.2 MHz, DMSO-d_6) spectrum of 1,3,6',2′,3''-penta-N-Cbz-6″-(2-guanidinoethylamino)-6''-deoxytobramycin **5a** 19
Figure S14. ^{13}C NMR (125.8 MHz, DMSO-d_6) spectrum of 1,3,6',2′,3''-penta-N-Cbz-6″-(2-guanidinoethylamino)-6''-deoxytobramycin **5a** 20
Figure S15. ^{1}H NMR (500.2 MHz, DMSO-d_6) spectrum of 1,3,6',2′,3''-penta-N-Cbz-6″-(3-guanidinpropil-1-amino)-6''-deoxytobramycin **5b** 21
Figure S16. ^{13}C NMR (125.8 MHz, DMSO-d_6) spectrum of 1,3,6',2′,3''-penta-N-Cbz-6″-(3-guanidinpropil-1-amino)-6''-deoxytobramycin **5b** 22
Figure S17. ^{1}H NMR (500.2 MHz, D_2O) spectrum of 6″-(2- Guanidinoethylamino)-6″-deoxytobramycin **6a** 23
Figure S18. ^{13}C NMR (125.8 MHz, D_2O) spectrum of 6″-(2- Guanidinoethylamino)-6″-deoxytobramycin **6a** 24
Figure S19. ^{1}H NMR (500.2 MHz, D_2O) spectrum of 6''-(3-Guanidinopropil-1-amino)-6''-deoxytobramycin **6b**..25
Figure S20. ^{13}C NMR (125.8 MHz, D_2O) spectrum of 6''-(3-Guanidinopropil-1-amino)-6''-deoxytobramycin **6b**..26
Figure S21. HRMS (ESI) spectrum of 1,3,6',2′,3''-penta-*N*-Cbz-tobramycin **2a** 27

Figure S22. HRMS (ESI) spectrum of 1,3,6′,2′,3″-penta-N-Cbz-6″-*O*-(2,4,6-triisopropylbenzosulfonyl)tobramycin **2b** 28
Figure S23. HRMS (ESI) spectrum of 6"-(2-aminoethyamino)-1,3,6',2′,3"-penta-*N*-Cbz-6"-deoxytobramycin **3a** 29
Figure S24. HRMS (ESI) spectrum of 6"-(3-aminopropyl-1-amino)-1,3,6',2′,3"-penta-*N*-Cbz-6"-deoxytobramycin **3b** 30
Figure S25. HRMS (ESI) spectrum of 6"-(2-aminoethyamino)-6"-deoxytobramycin **4a** 31
Figure S26. HRMS (ESI) spectrum of 6″-(3-aminopropyl-1-amino)- 6″-deoxytobramycin **4b** 32
Figure S27. HRMS (ESI) spectrum of 1,3,6',2′,3"-penta-N-Cbz-6″-(2-guanidinoethylamino)-6"-deoxytobramycin **5a** 33
Figure S28. HRMS (ESI) spectrum of 1,3,6',2′,3"-penta-N-Cbz-6″-(3-guanidinpropil-1-amino)-6"-deoxytobramycin **5b** 34
Figure S29. HRMS (ESI) spectrum of 6″-(2- Guanidinoethylamino)-6″- deoxytobramycin **6a** 35
Figure S30. HRMS (ESI) spectrum of 6"-(3-Guanidinopropil-1-amino)-6"-deoxytobramycin **6b** 36
Figure S31. HPLC chromatogram of 1,3,6',2′,3"-penta-*N*-Cbz-tobramycin **2a** 37
Figure S32. HPLC chromatogram of 1,3,6′,2′,3″-penta-N-Cbz-6″-*O*-(2,4,6-triisopropylbenzosulfonyl)tobramycin **2b** 38
Figure S33. HPLC chromatogram of 6"-(2-aminoethyamino)-1,3,6',2′,3"-penta-*N*-Cbz-6"-deoxytobramycin **3a** 39
Figure S34. HPLC chromatogram of 6"-(3-aminopropyl-1-amino)-1,3,6',2′,3"-penta-*N*-Cbz-6"-deoxytobramycin **3b** 40
Figure S35. HPLC chromatogram of 1,3,6',2′,3"-penta-N-Cbz-6″-(2-guanidinoethylamino)-6"-deoxytobramycin **5a** 41
Figure S36. HPLC chromatogram of 1,3,6',2′,3"-penta-N-Cbz-6″-(3-guanidinpropil-1-amino)-6"-deoxytobramycin **5b** 42
Figure S37. Drop-test for *E. coli* strains with P610T/L substitution in EF-G. 43
Figure S38. Cell viability curves of HEK293T cell line incubated with tested substances. 43

Table S1. ^{1}H and ^{13}C NMR data of tobramycin derivatives **2a-b**, **3a**-**3b** in DMSO-d_6.

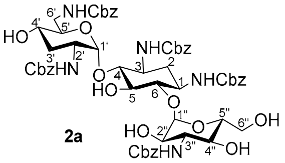

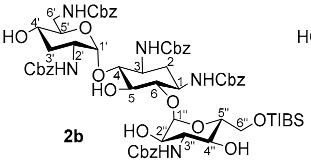

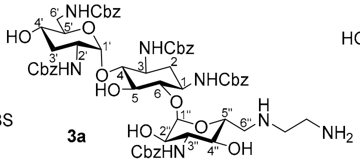

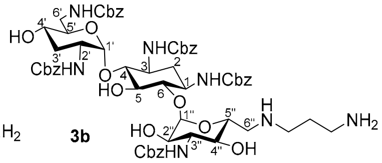

	Chemical shift, ppm							
Compound	**2a**		**2b**		**3a**		**3b**	
Position of C atom	^{1}H NMR	^{13}C NMR	^{1}H NMR	^{13}C NMR	^{1}H NMR	^{13}C NMR	^{1}H NMR	^{13}C NMR
1’	5.00	97.1	4.99	97.1	5.03	97.1	5.02	97.2
2’	3.58	49.9	3.56	50.0	3.58	50.0	3.58	50.0
3’	1.86/1.59	33.1	1.87/1.56	33.1	1.88/1.60	33.2	1.88/1.60	33.2
4’	3.34	64.6	3.34	64.5	3.34	64.6	3.34	64.6
5’	3.55	71.1	3.54	71.6	3.55	71.8	3.55	71.8
6’	3.38/3.19	41.6	3.37/3.20	41.5	3.39/3.18	41.7	3.39/3.18	41.6
1	3.53	49.9	3.50	49.9	3.53	49.9	3.53	49.9
2	1.90/1.44	34.4	1.84/1.40	34.6	1.90/1.48	34.2	1.89/1.47	34.2
3	3.54	49.6	3.56	49.6	3.52	49.7	3.51	49.6
4	3.44	81.7	3.44	81.7	3.45	82.1	3.45	82.1
5	3.64	73.9	3.59	73.8	3.64	73.9	3.66	73.8
6	3.43	81.6	3.42	80.8	3.42	81.7	3.42	81.8
1”	5.01	97.1	5.02	96.8	4.97	97.1	4.97	97.0
2”	3.37	69.8	3.35	69.5	3.38	69.9	3.38	69.8
3”	3.59	56.4	3.61	56.4	3.55	56.5	3.54	56.5
4”	3.33	67.0	3.37	66.4	3.21	69.6	3.20	69.8
5”	3.79	73.2	4.16	69.8	3.85	71.4	3.85	71.2
6”	3.53/3.53	60.1	4.30/4.08	67.4	2.71/2.51	49.8	2.69/2.51	50.1
1-N	7.17	-	7.17	-	7.18	-	7.17	-
3-N	7.36	-	7.36	-	7.44	-	7.44	-
2’-N	6.96	-	6.89	-	7.00	-	7.01	-
6’-N	6.91	-	6.96	-	6.91	-	6.92	-
3”-N	7.03	-	7.07	-	6.66	-	6.66	-
Ar (Cbz)	7.26-7.38	128.2/127.6/137.2/137.1/137.0/136.9	7.26-7.38	137.2/137.1/137.0/136.9/128.2/127.6/127.4	7.26-7.38	137.2/137.1/137.0/136.9/128.3/128.2/127.5/127.4	7.26-7.38	137.0/128.2/127.6
CH_2 (Cbz)	4.86-5.11	65.2/65.1/65.0/64.9	4.95-5.21	65.2/65.1/65.0	4.86-5.10	65.2/65.1/65.0/64.9	4.86-5.10	65.2/64.9/64.6
C=O (Cbz)	-	156.5/156.4/155.8/155.6/155.4	-	156.6/156.5/155.6/155.5/155.3	-	156.5/156.4/155.8/155.6/155.4	-	156.5/155.8/155.6/155.4
Substituent 6”			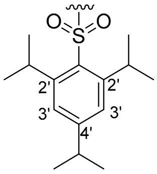		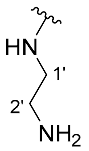		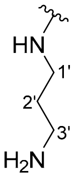	
1’			-	153.5	2.44	52.2	2.44	47.4
2’			-	150.1	2.52	41.0	1.40	33.0
3’			7.29	123.7			2.50	39.6
4’			-	129.1				
2’-CH			4.04	29.0				
2’-CH_3			1.19	23.4				
4’-CH			2.92	33.3				
4’-CH_3			1.18	23.2				

Table S2. ^{1}H and ^{13}C NMR data of tobramycin derivatives **5a-b** in DMSO-d_6

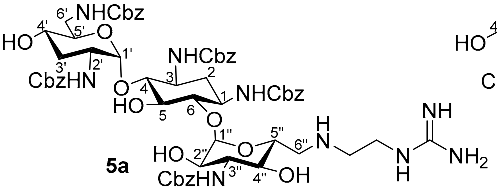

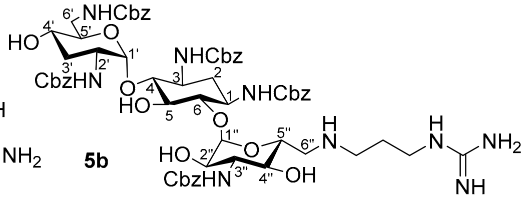

	Chemical shift, ppm			
Compound	**5a**		**5b**	
Position of C atom	^{1}H NMR	^{13}C NMR	^{1}H NMR	^{13}C NMR
1’	5.03	97.0	5.01	97.1
2’	3.58	50.0	3.53	49.6
3’	1.88/1.59	33.1	1.88/1.59	33.2
4’	3.35	64.5	3.35	64.6
5’	3.55	71.7	3.55	71.7
6’	3.38/3.19	41.6	3.37/3.19	41.6
1	3.52	49.9	3.52	49.9
2	1.89/1.48	34.4	1.88/1.43	34.5
3	3.53	49.6	3.57	50.0
4	3.42	82.6	3.44	81.7
5	3.65	73.2	3.64	73.2
6	3.44	80.9	3.42	80.7
1”	4.98	97.4	4.98	97.3
2”	3.37	69.8	3.37	69.8
3”	3.58	56.3	3.57	56.4
4”	3.24	69.1	3.22	69.3
5”	3.89	71.4	3.87	71.2
6”	2.69/2.61	49.5	2.65/2.53	49.6
1-N	7.15	-	7.13	-
3-N	7.36	-	n.o.	-
2’-N	6.97	-	6.98	-
6’-N	6.87	-	6.86	-
3”-N	6.56	-	n.o.	-
Ar (Cbz)	7.26-7.38	137.1/128.3/127.6	7.26-7.38	137.1/128.3/127.6
CH_2 (Cbz)	4.86-5.10	65.3/65.0/64.9	4.86-5.10	65.3/65.0/64.9
C=O (Cbz)	-	157.6/157.3/156.5/155.8/155.7/155.6/155.4	-	157.6/157.3/156.5/155.8/155.7/155.6/155.4
Substituent 6”	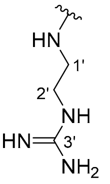		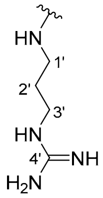	
1’	2.62/2.52	48.4	2.44/2.37	46.0
2’	3.09	41.0	1.49	28.9
3’	-	162.2	3.04	38.5
4’			-	162.3

Table S3. ^{1}H and ^{13}C NMR data of tobramycin derivatives **4a-b**, **6a-b** in D_2O.

4a 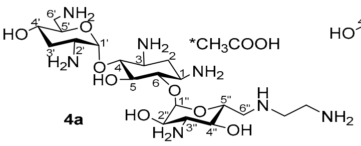

4b 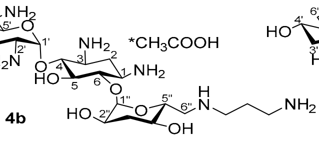

6a 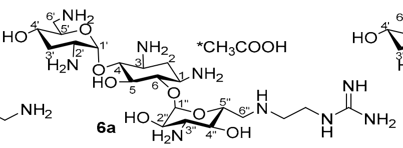

6b 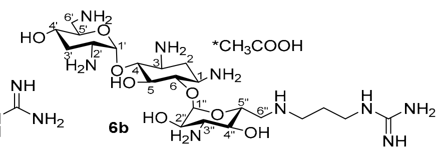

	Chemical shift, ppm							
Compound	**4a**		**4b**		**6a**		**6b**	
Position of C atom	^{1}H NMR	^{13}C NMR	^{1}H NMR	^{13}C NMR	^{1}H NMR	^{13}C NMR	^{1}H NMR	^{13}C NMR
1’	5.53	99.1	5.54	99.2	5.67	97.6	5.65	98.0
2’	3.36	51.3	3.38	51.3	3.57	51.0	3.51	51.1
3’	2.22/1.86	35.2	2.22/1.86	35.0	2.25/1.96	33.5	2.27/1.93	33.9
4’	3.64	68.4	3.65	68.3	3.69	67.9	3.67	68.0
5’	3.90	72.5	3.93	72.4	3.97	72.4	3.94	72.4
6’	3.41/3.17	43.3	3.42/3.19	43.2	3.44/3.23	43.1	3.43/3.20	43.1
1	3.09	51.9	3.09	51.8	3.18	51.7	3.15	51.7
2	2.18/1.51	35.9	2.19/1.51	35.6	2.27/1.63	34.2	2.26/1.61	34.6
3	3.25	57.4	3.29	57.4	3.45	57.6	3.41	57.4
4	3.53	88.7	3.55	88.4	3.66	87.8	3.64	87.9
5	3.73	77.4	3.74	77.4	3.78	77.4	3.76	77.5
6	3.55	85.5	3.56	85.7	3.66	84.1	3.63	84.6
1”	5.10	103.0	5.11	103.1	5.14	103.3	5.13	103.2
2”	3.75	72.8	3.77	72.7	3.97	71.5	3.95	71.4
3”	3.23	53.4	3.28	53.5	3.41	53.3	3.36	53.5
4”	3.48	72.0	3.45	72.0	3.59	70.7	3.55	71.0
5”	4.00	73.8	4.08	72.5	4.07	73.1	4.16	71.6
6”	3.00/2.86	51.5	3.20/3.06	51.5	3.18/3.04	51.3	3.40/3.21	51.0
1-N	-	-	-	-	-	-	-	-
3-N	-	-	-	-	-	-	-	-
2’-N	-	-	-	-	-	-	-	-
6’-N	-	-	-	-	-	-	-	-
3”-N	-	-	-	-	-	-	-	-
Substituent 6”	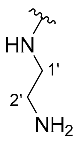		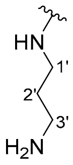		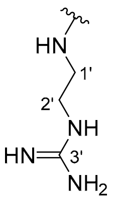		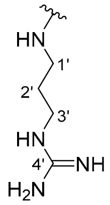	
1’	2.94	49.0	2.95	48.6	3.06	49.8	3.11	48.6
2’	3.12	41.1	1.99	27.8	3.45	42.1	2.00	27.9
3’			3.07	40.1	-	159.9	3.30	41.2
4’							-	159.7
CH_3 (Acetate)	1.92	26.2	1.92	26.1	1.92	26.1	1.92	26.1
COOH (Acetate)	-	184.3	-	184.3	-	184.2	-	184.2

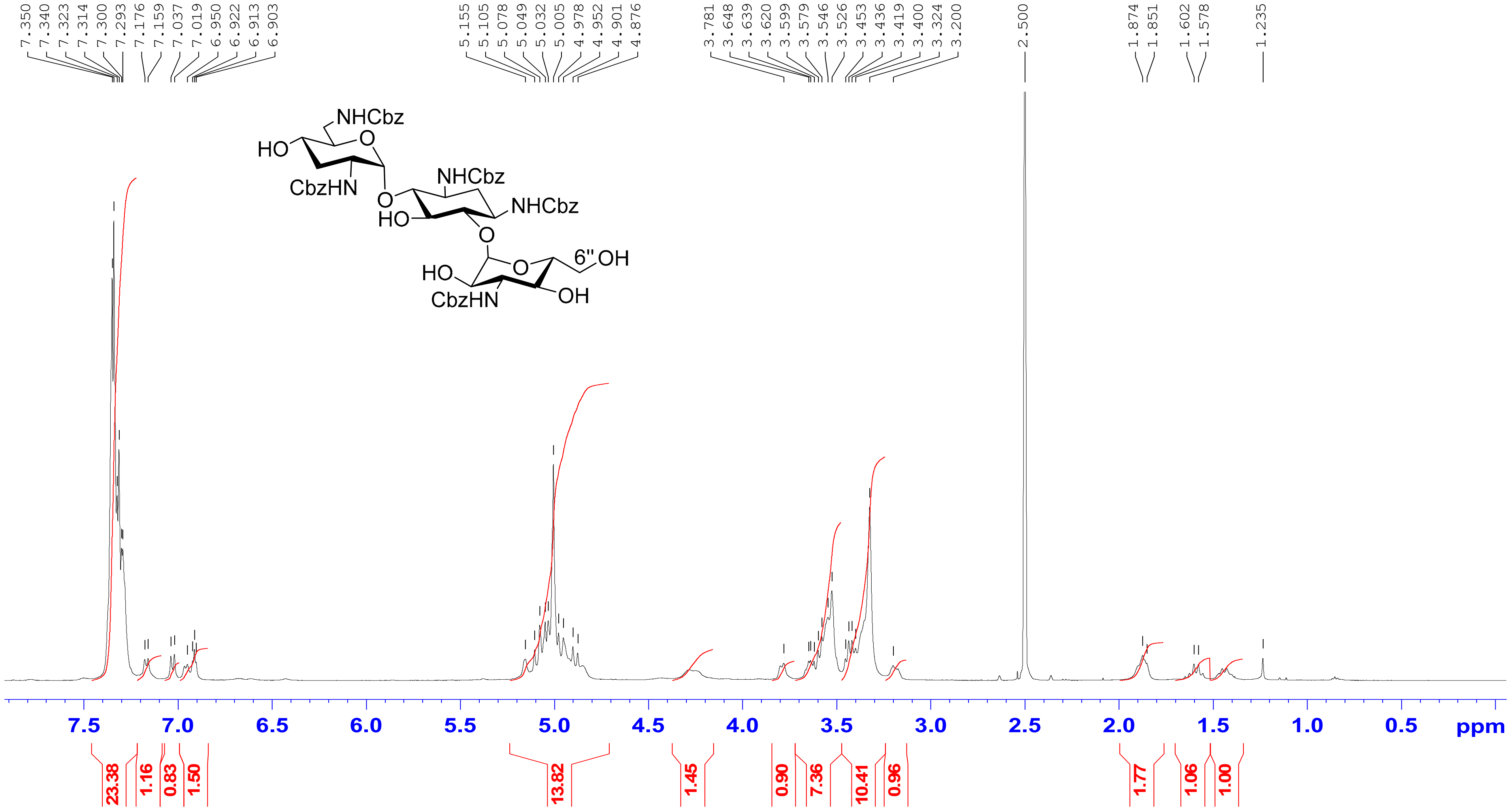

Figure S1. ^{1}H NMR (500.2 MHz, DMSO-d_6) spectrum of 1,3,6',2',3''-penta-*N*-Cbz-tobramycin **2a**

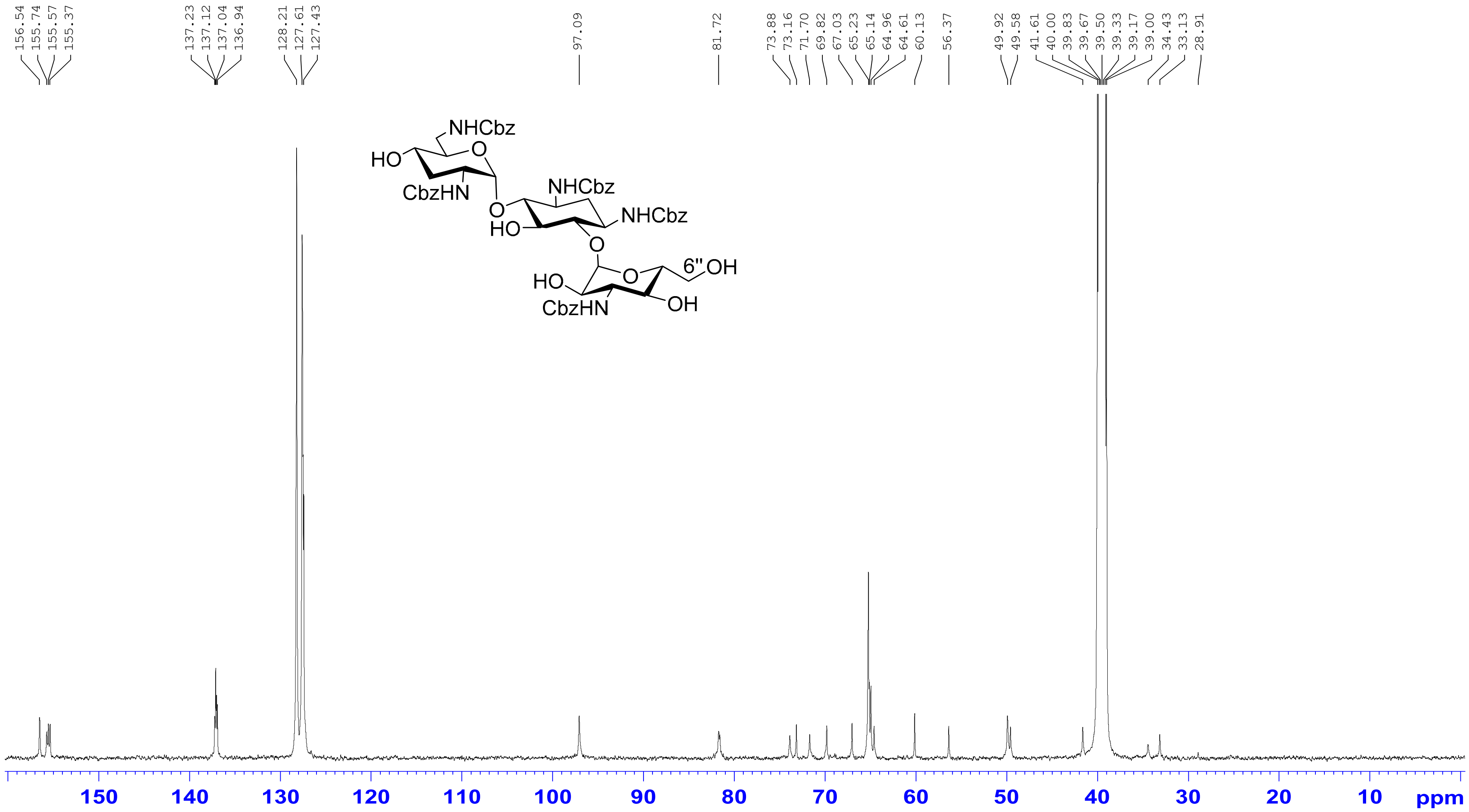

Figure S2. ^{13}C NMR (125.8 MHz, DMSO-d_6) spectrum of 1,3,6',2′,3''-penta-*N*-Cbz-tobramycin **2a**

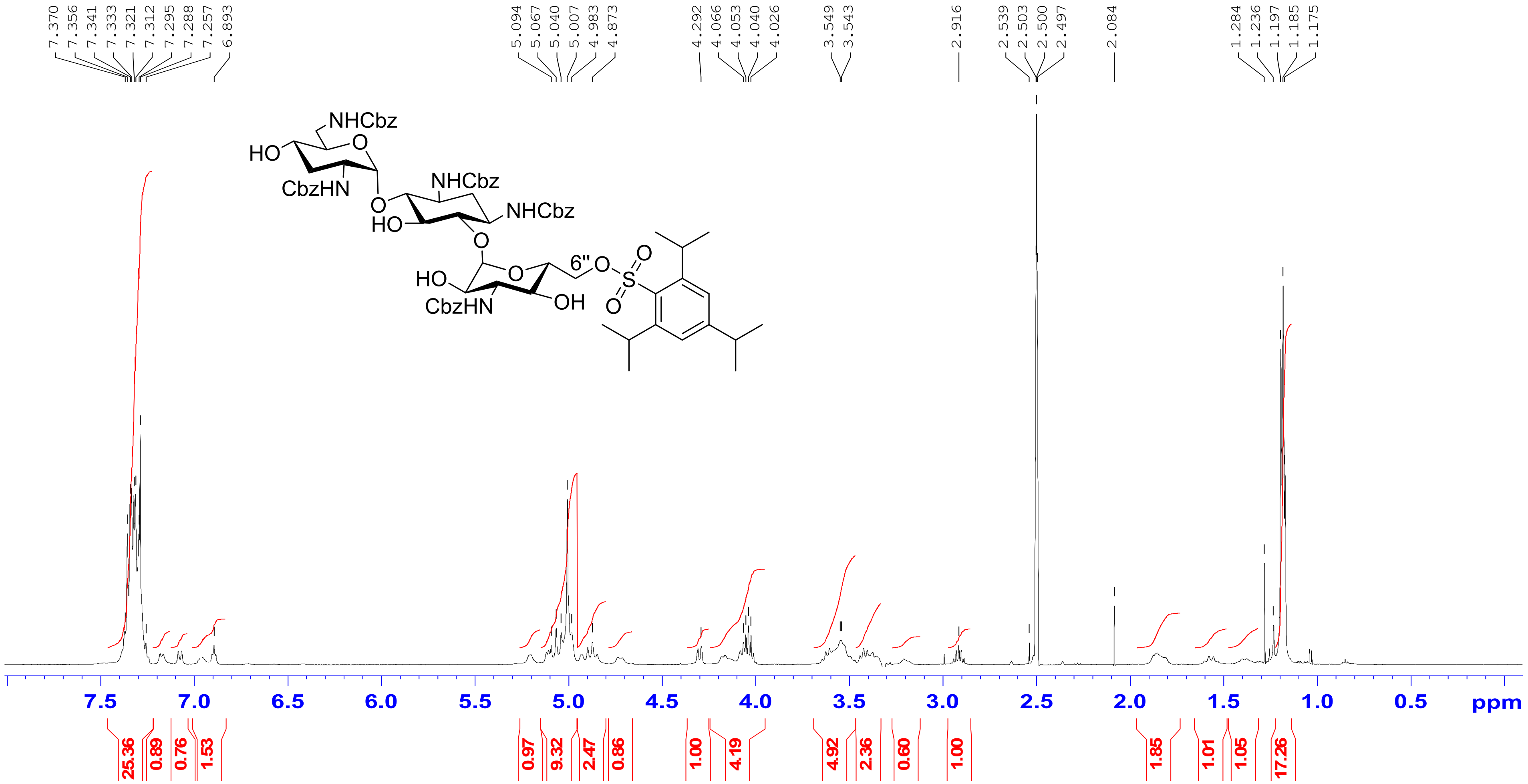

Figure S3. ^{1}H NMR (500.2 MHz, DMSO-*d*$_6$) spectrum of 1,3,6′,2′,3″-penta-N-Cbz-6″-*O*-(2,4,6-triisopropylbenzosulfonyl)tobramycin **2b**

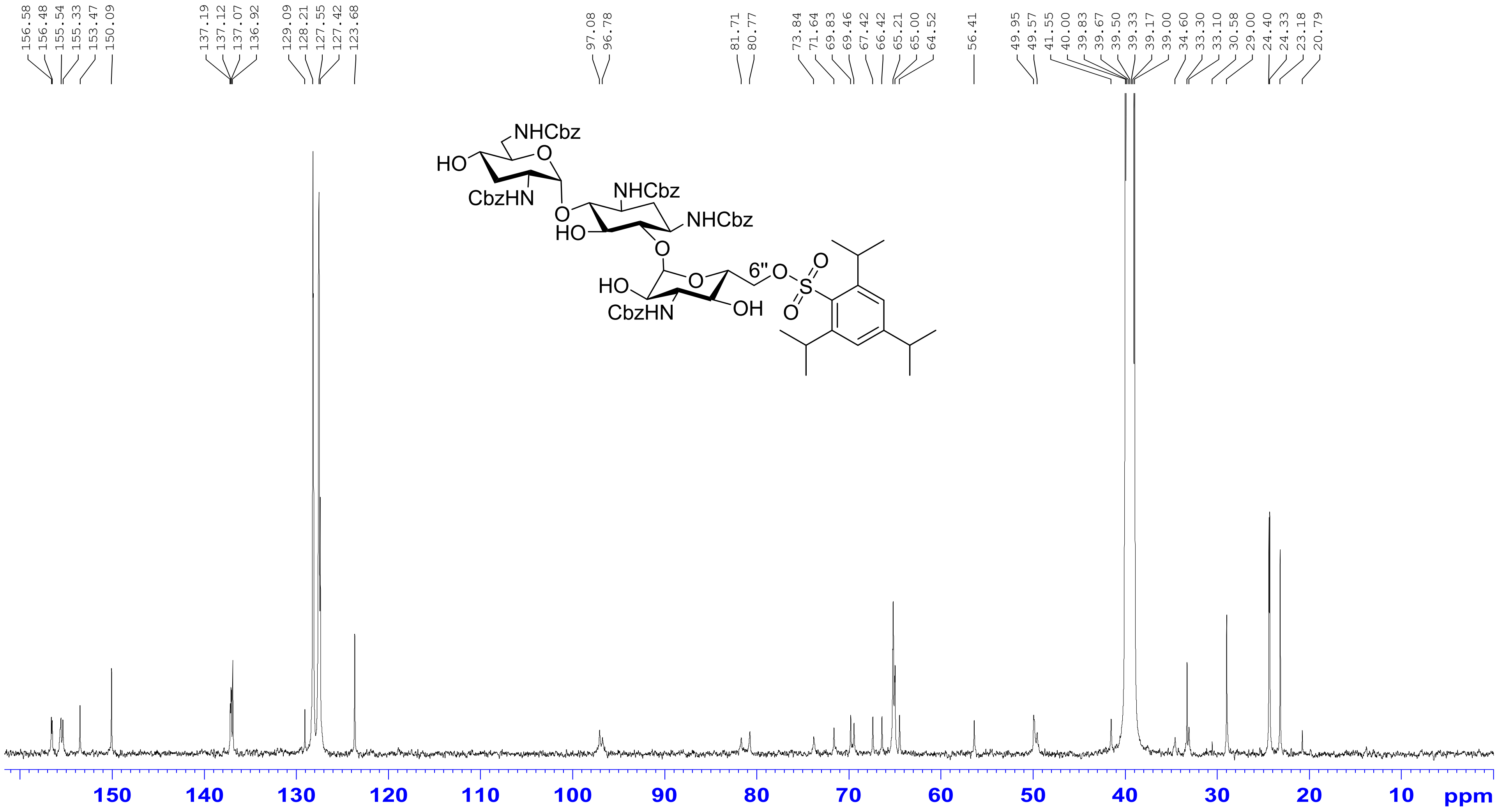

Figure S4. ^{13}C NMR (125.8 MHz, DMSO-d_6) spectrum of of N-Cbz-6″-*O*-(2,4,6-triisopropylbenzosulfonyl)tobramycin **2b**

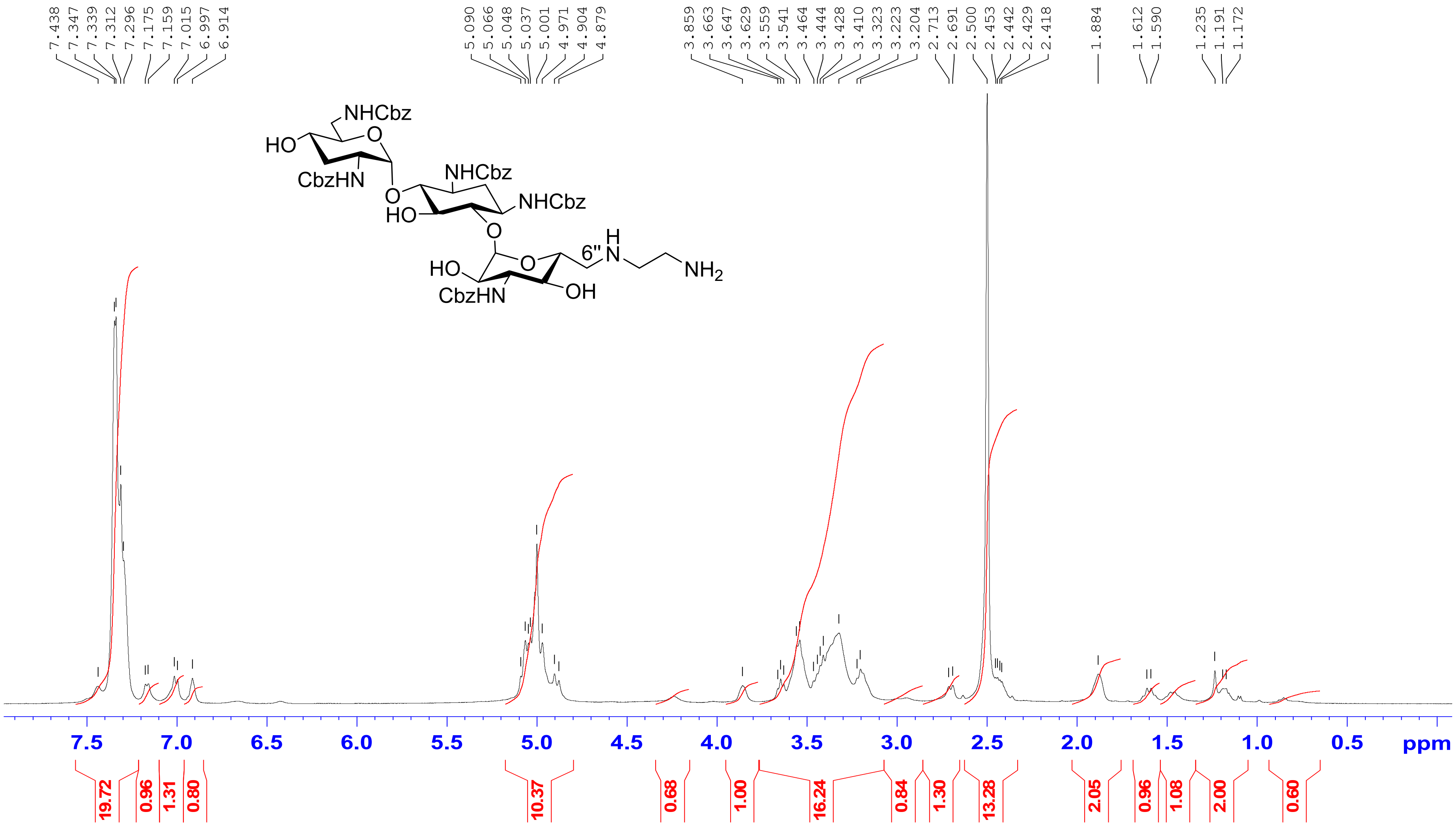

Figure S5. ^{1}H NMR (500.2 MHz, DMSO-d_6) spectrum of 6''-(2-aminoethyamino)-1,3,6',2′,3''-penta-*N*-Cbz-6''-deoxytobramycin **3a**

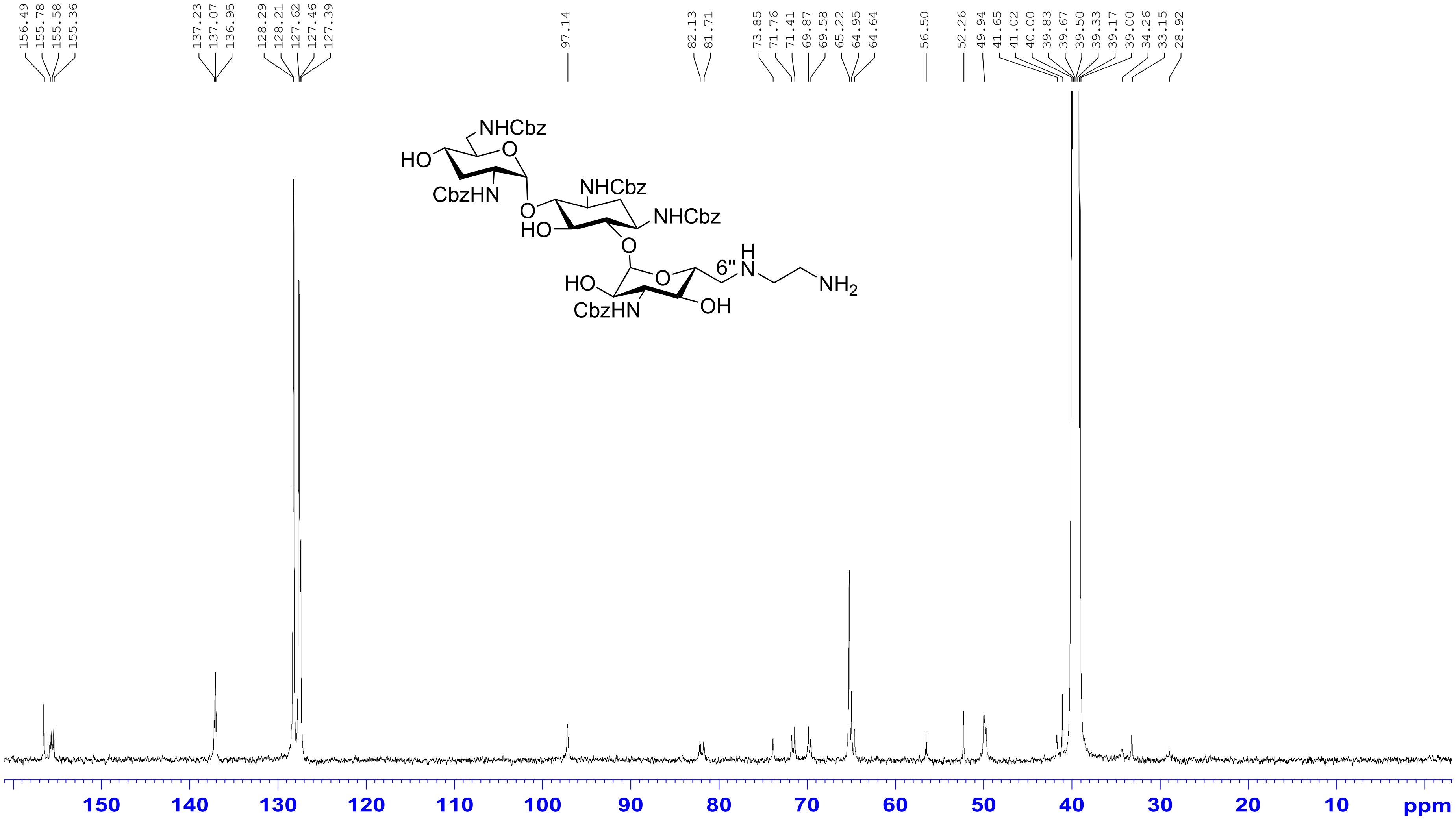

Figure S6. ^{13}C NMR (125.8 MHz, DMSO-d_6) spectrum of 6''-(2-aminoethyamino)-1,3,6',2′,3''-penta-*N*-Cbz-6''-deoxytobramycin **3a**

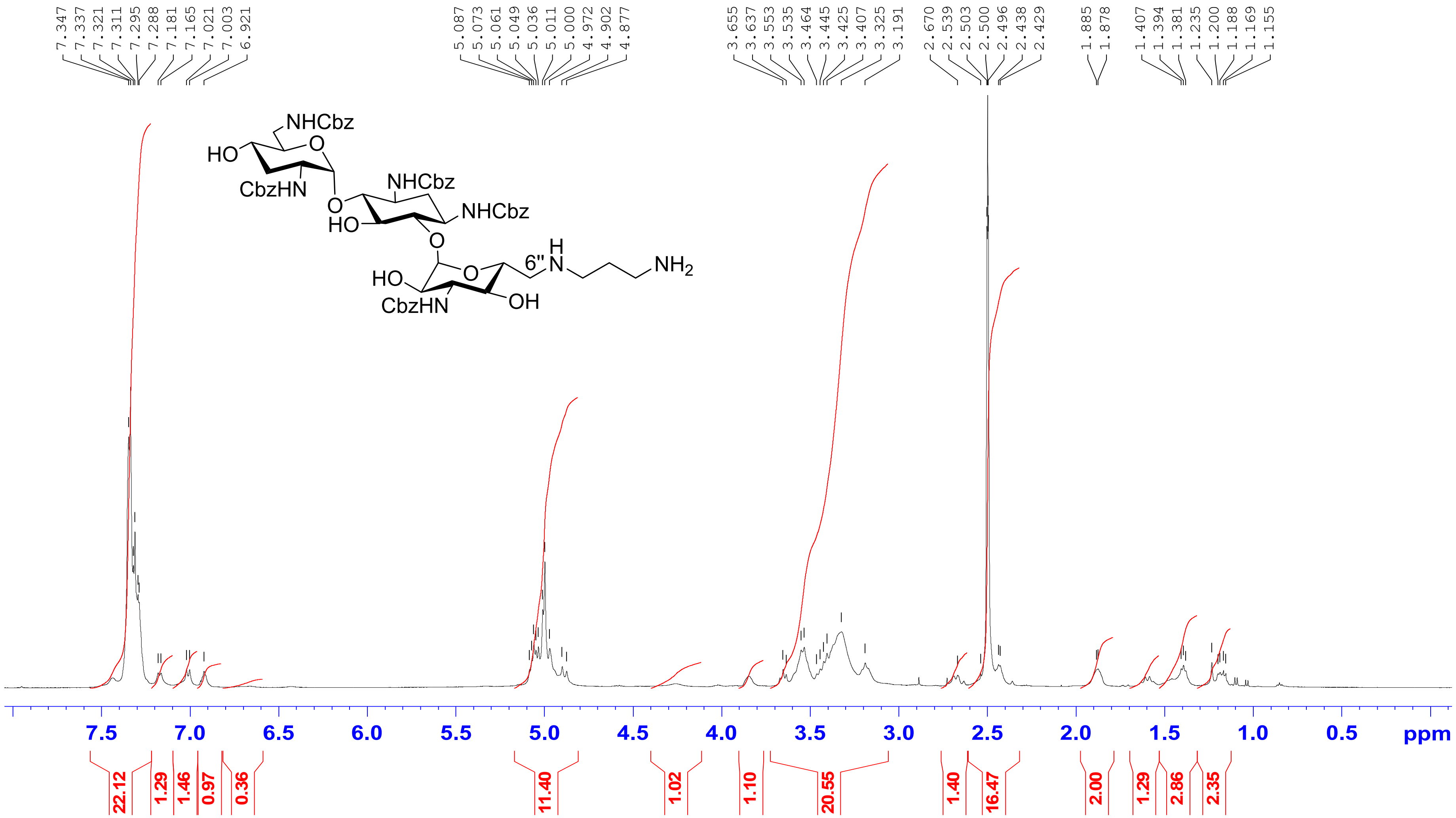

Figure S7. ¹H NMR (500.2 MHz, DMSO-*d*₆) spectrum of 6''-(3-aminopropyl-1-amino)-1,3,6',2',3''-penta-*N*-Cbz-6''-deoxytobramycin **3b**

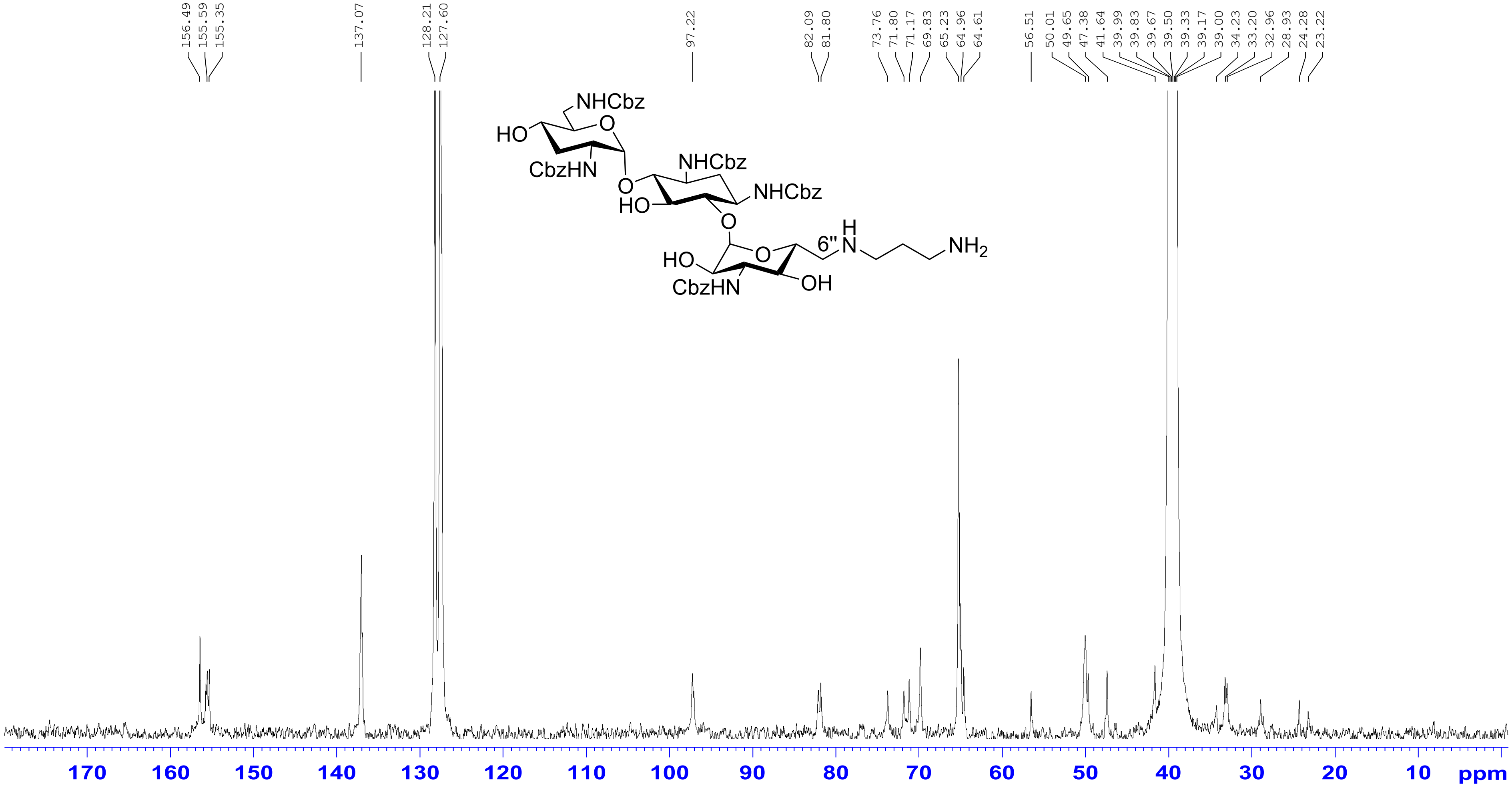

Figure S8. ^{13}C NMR (125.8 MHz, DMSO-d_6) spectrum of 6''-(3-aminopropyl-1-amino)-1,3,6',2',3''-penta-*N*-Cbz-6''-deoxytobramycin **3b**

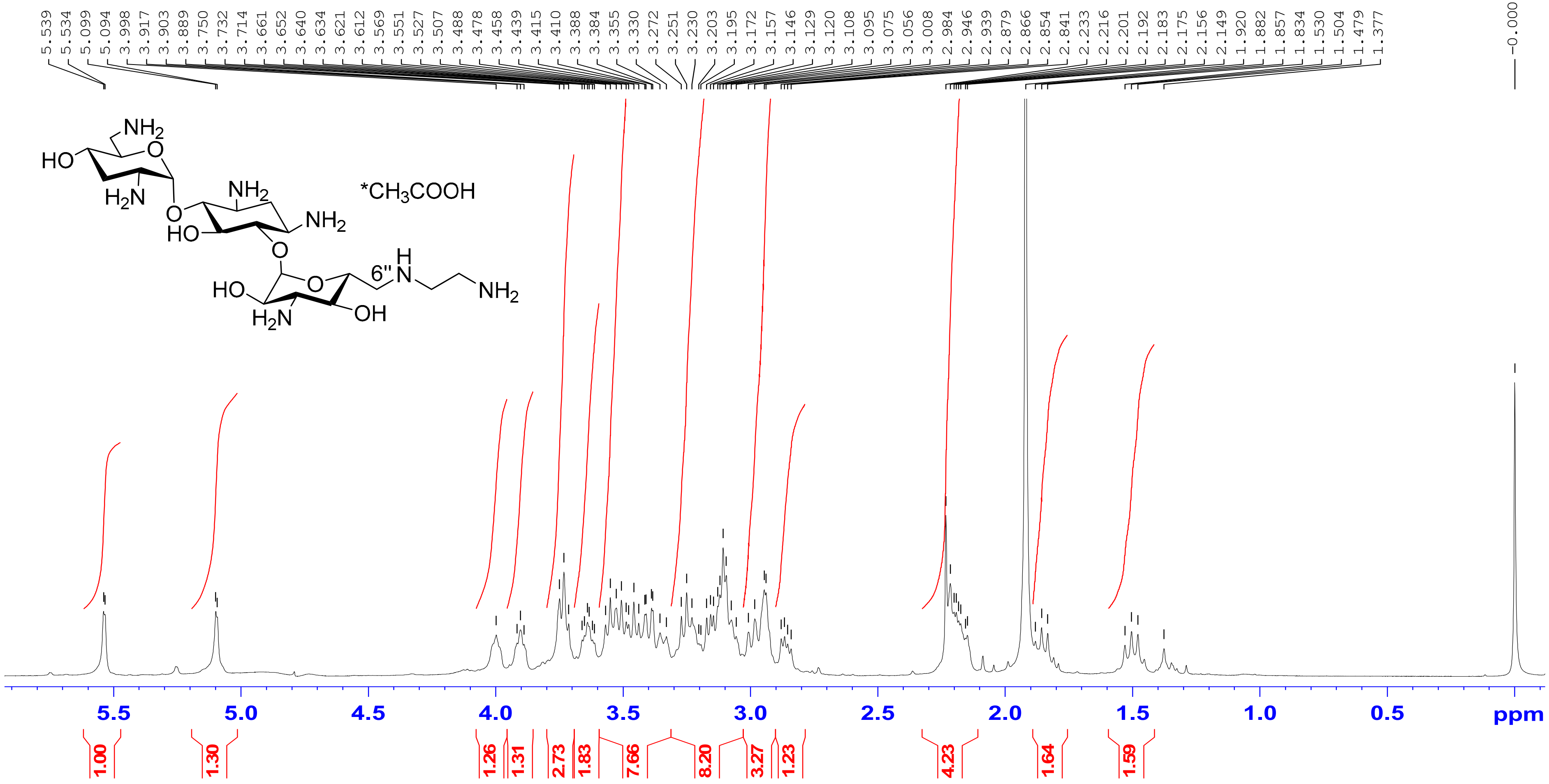

Figure S9. ^{1}H NMR (500.2 MHz, D_2O) spectrum of 6''-(2-aminoethyamino)-6''-deoxytobramycin **4a**

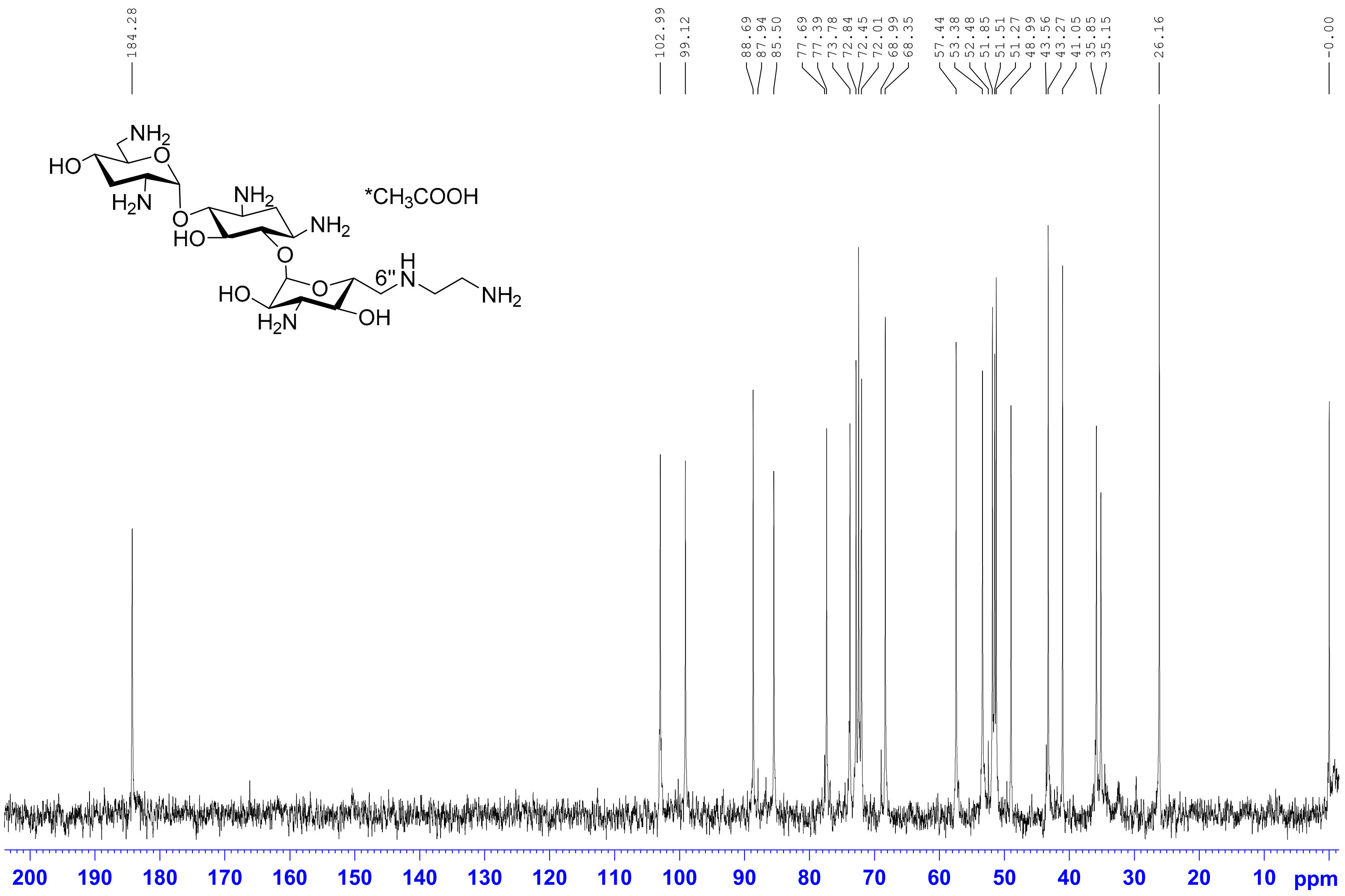

Figure S10. ^{13}C NMR (125.8 MHz, D_2O) spectrum of 6''-(2-aminoethyamino)-6''-deoxytobramycin **4a**

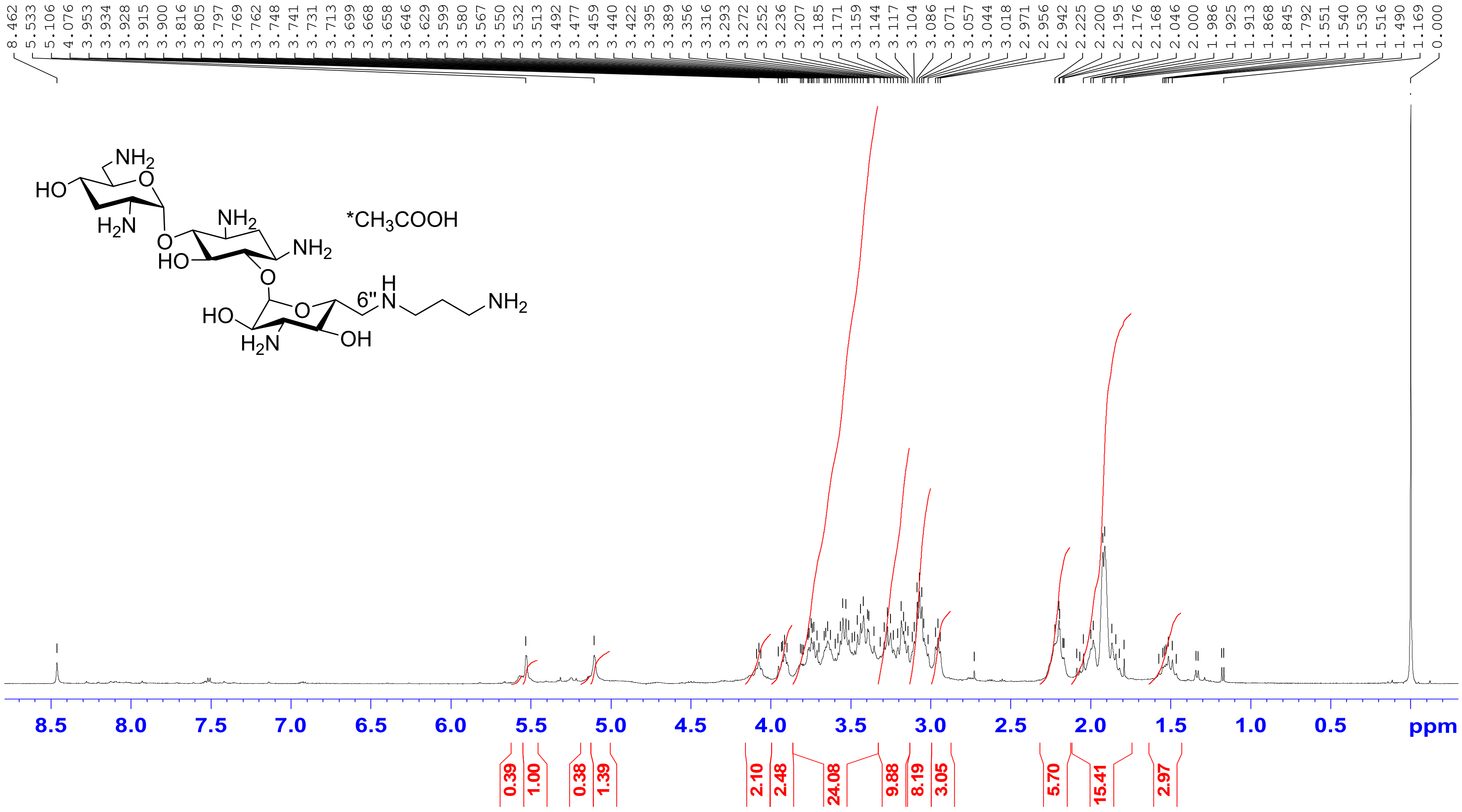

Figure S11. ^{1}H NMR (500.2 MHz, D_2O) spectrum of 6″-(3-Aminopropyl-1-amino)- 6″-deoxytobramycin **4b**

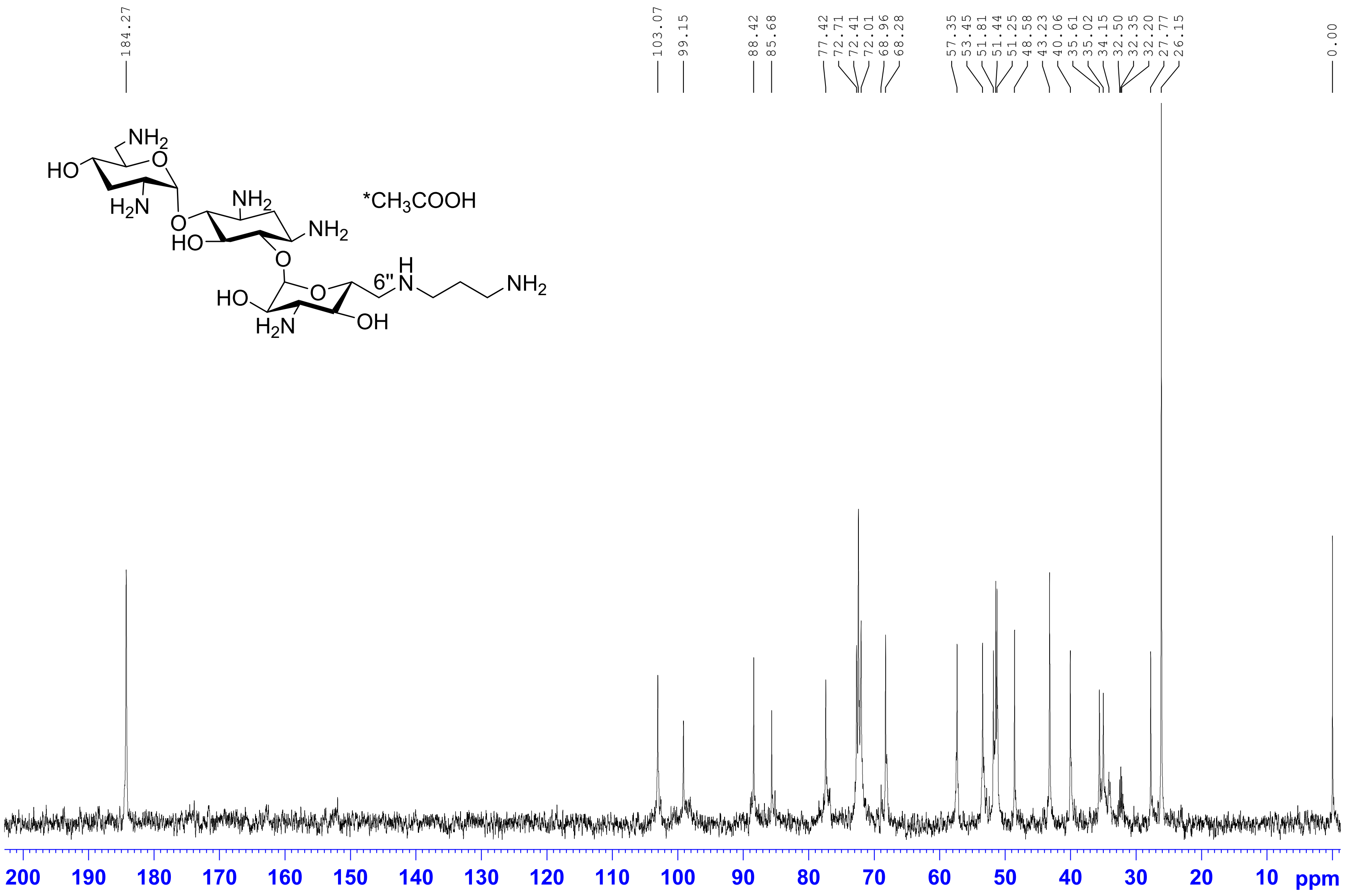

Figure S12. ^{13}C NMR (125.8 MHz, D_2O) spectrum of 6″-(3-Aminopropyl-1-amino)- 6″-deoxytobramycin **4b**

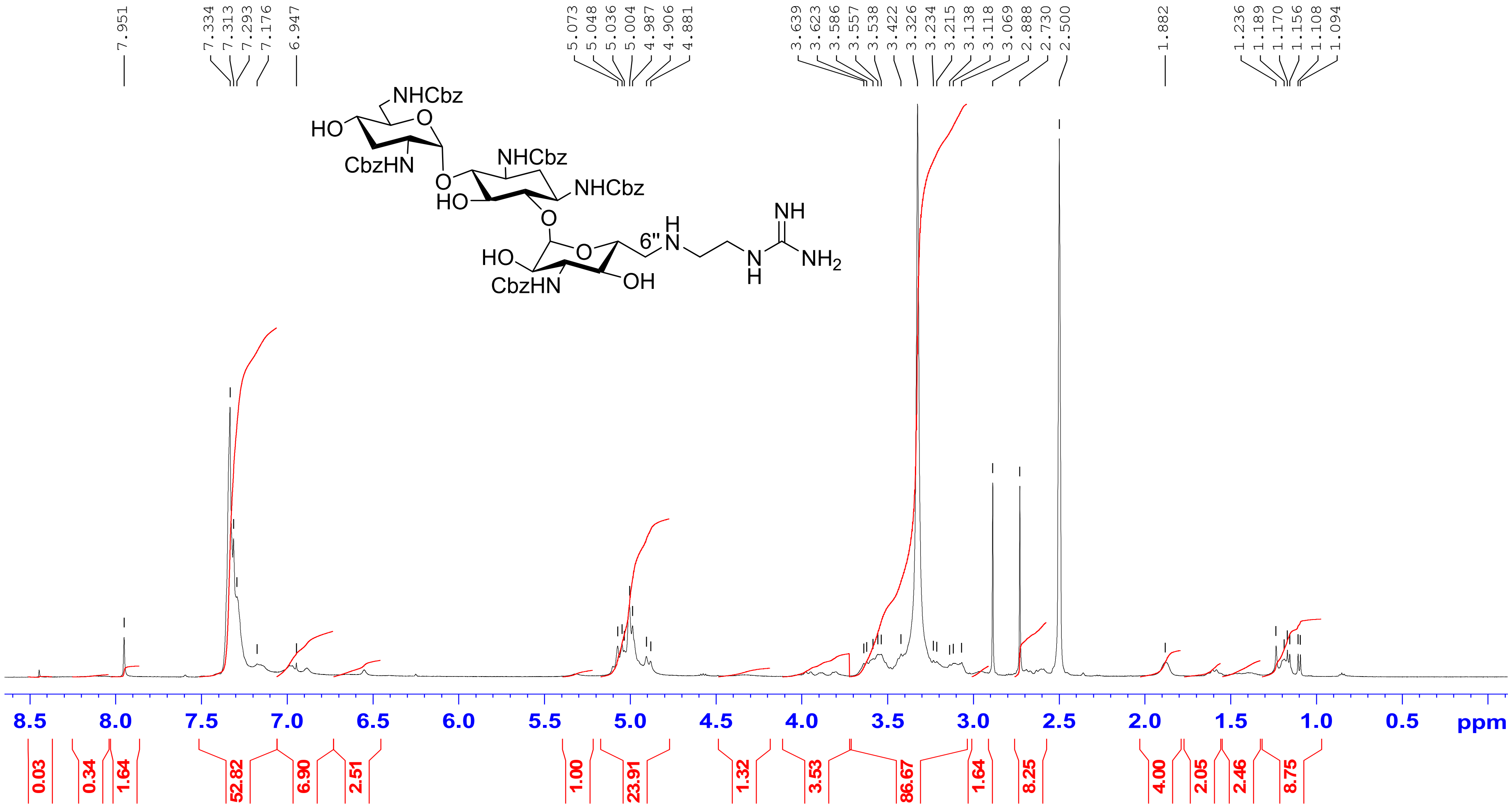

Figure S13. ^{1}H NMR (500.2 MHz, DMSO-d_6) spectrum of 1,3,6',2',3''-penta-N-Cbz-6''-(2-guanidinoethylamino)-6''-deoxytobramycin **5a**

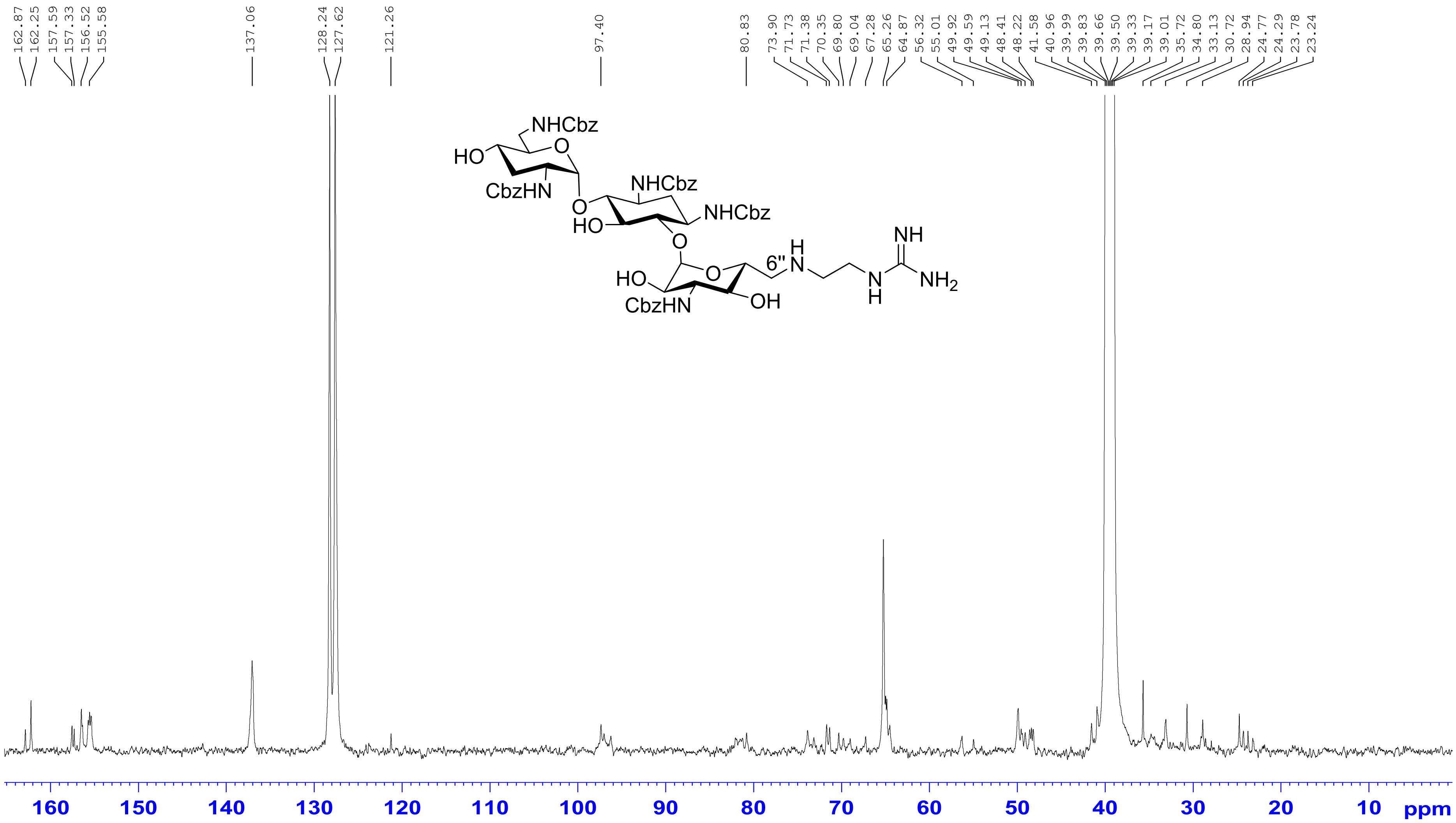

Figure S14. ^{13}C NMR (125.8 MHz, DMSO-d_6) spectrum of 1,3,6',2′,3''-penta-N-Cbz-6″-(2-guanidinoethylamino)-6''-deoxytobramycin **5a**

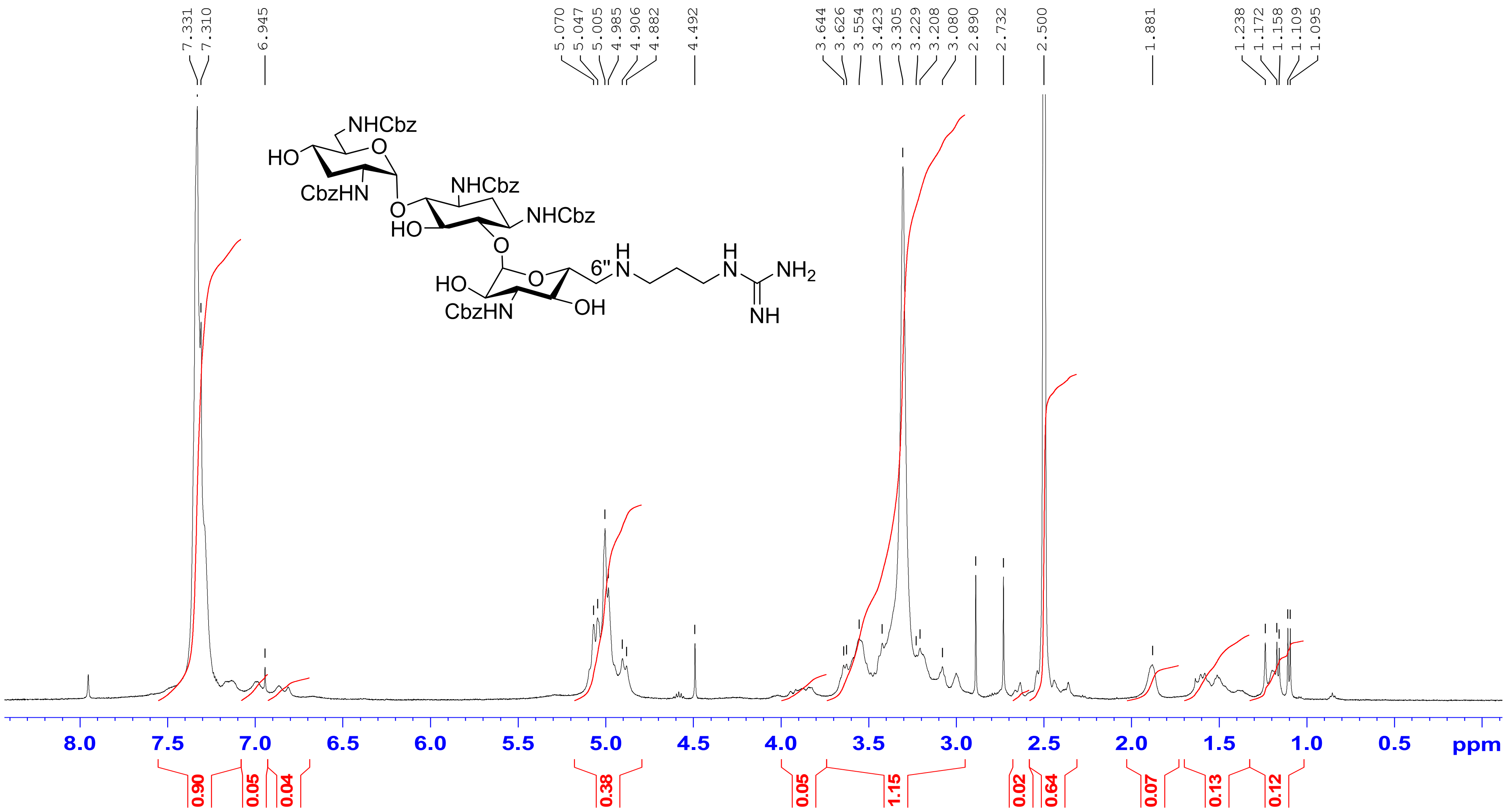

Figure S15. ^{1}H NMR (500.2 MHz, DMSO-d_6) spectrum of 1,3,6',2',3''-penta-N-Cbz-6''-(3-guanidinpropil-1-amino)-6''-deoxytobramycin **5b**

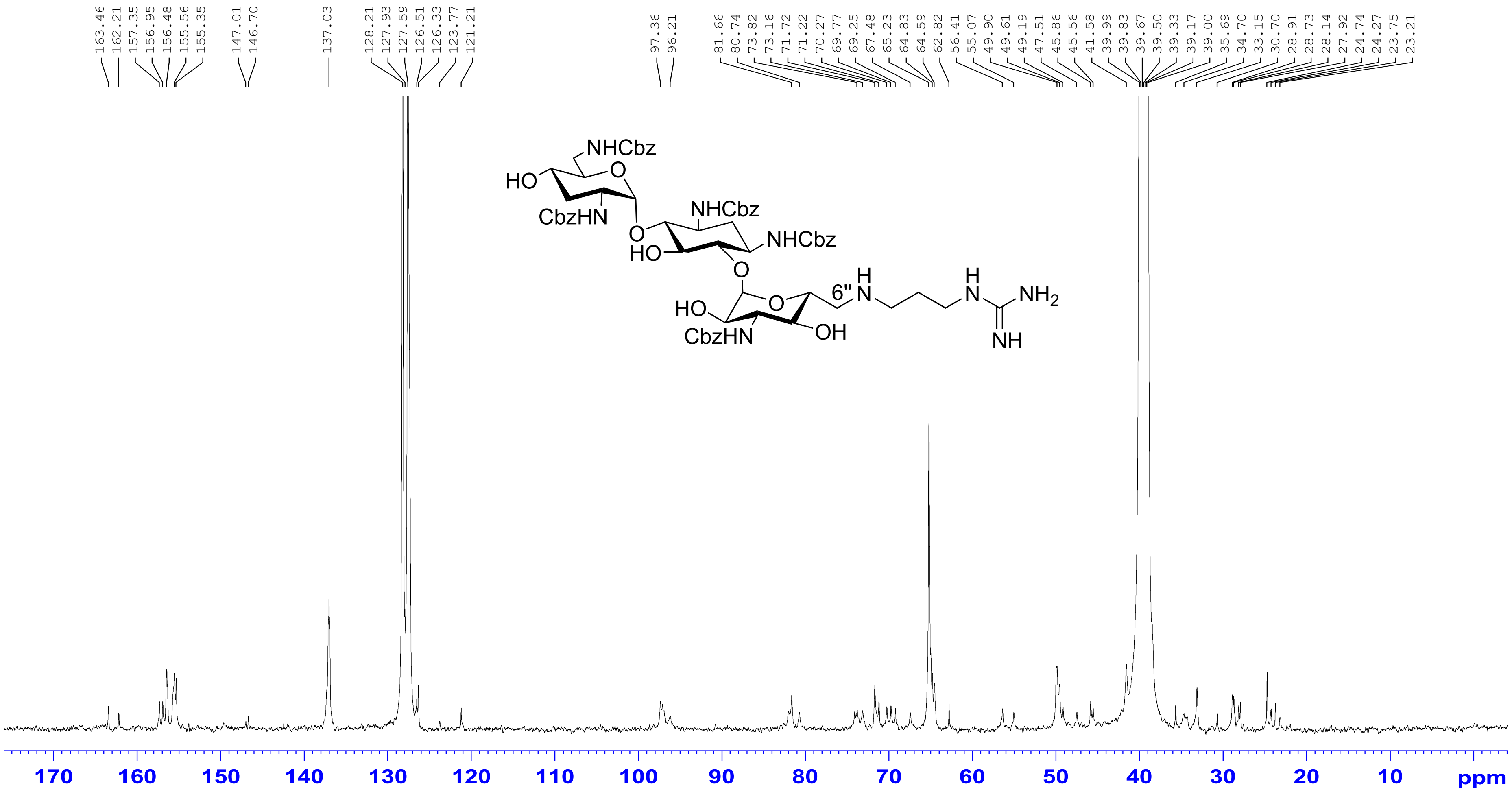

Figure S16. ^{13}C NMR (125.8 MHz, DMSO-d_6) spectrum of 1,3,6',2′,3''-penta-N-Cbz-6″-(3-guanidinpropil-1-amino)-6''-deoxytobramycin **5b**

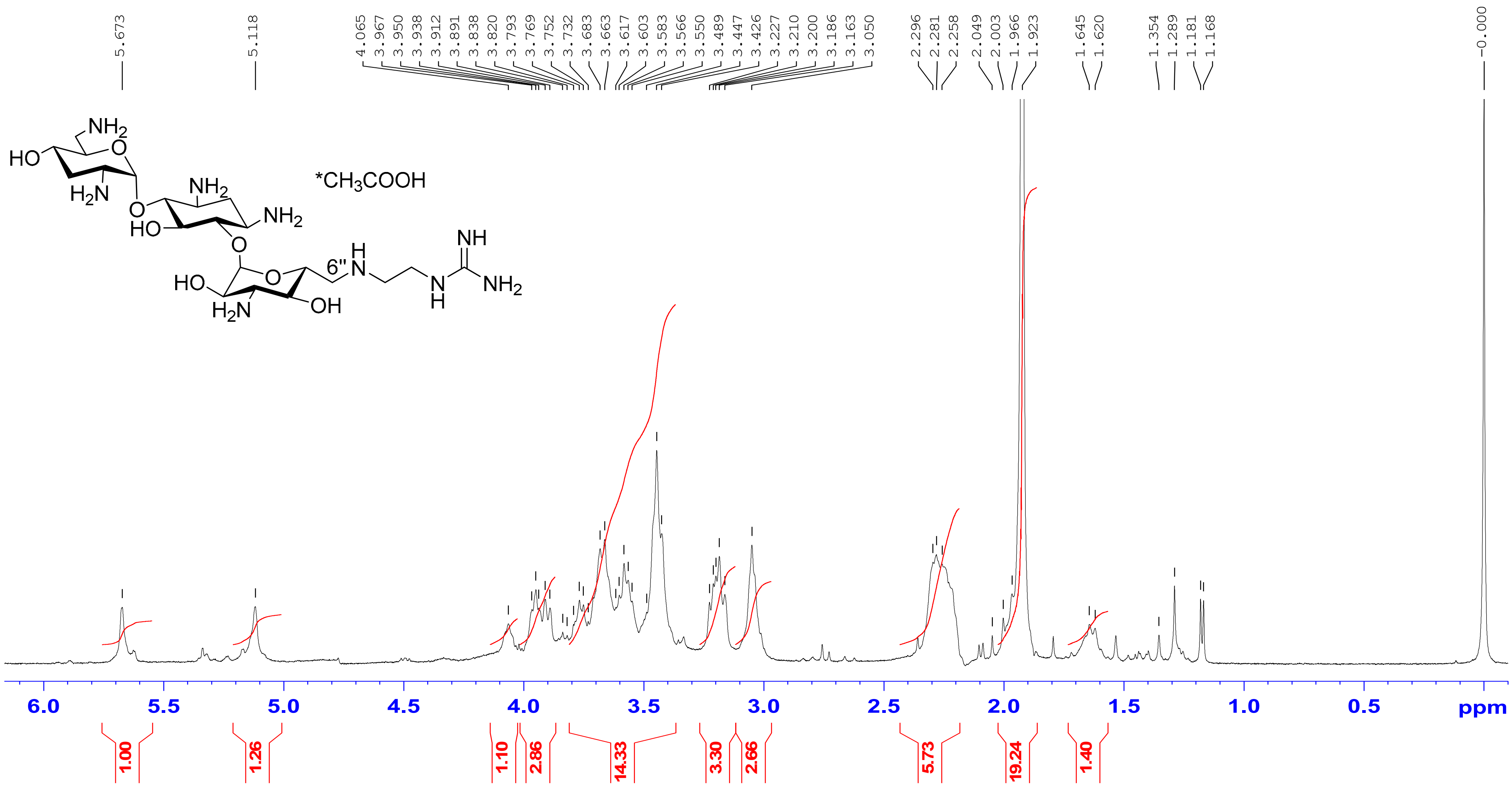

Figure S17. ^{1}H NMR (500.2 MHz, D_2O) spectrum of 6″-(2- Guanidinoethylamino)-6″- deoxytobramycin **6a**

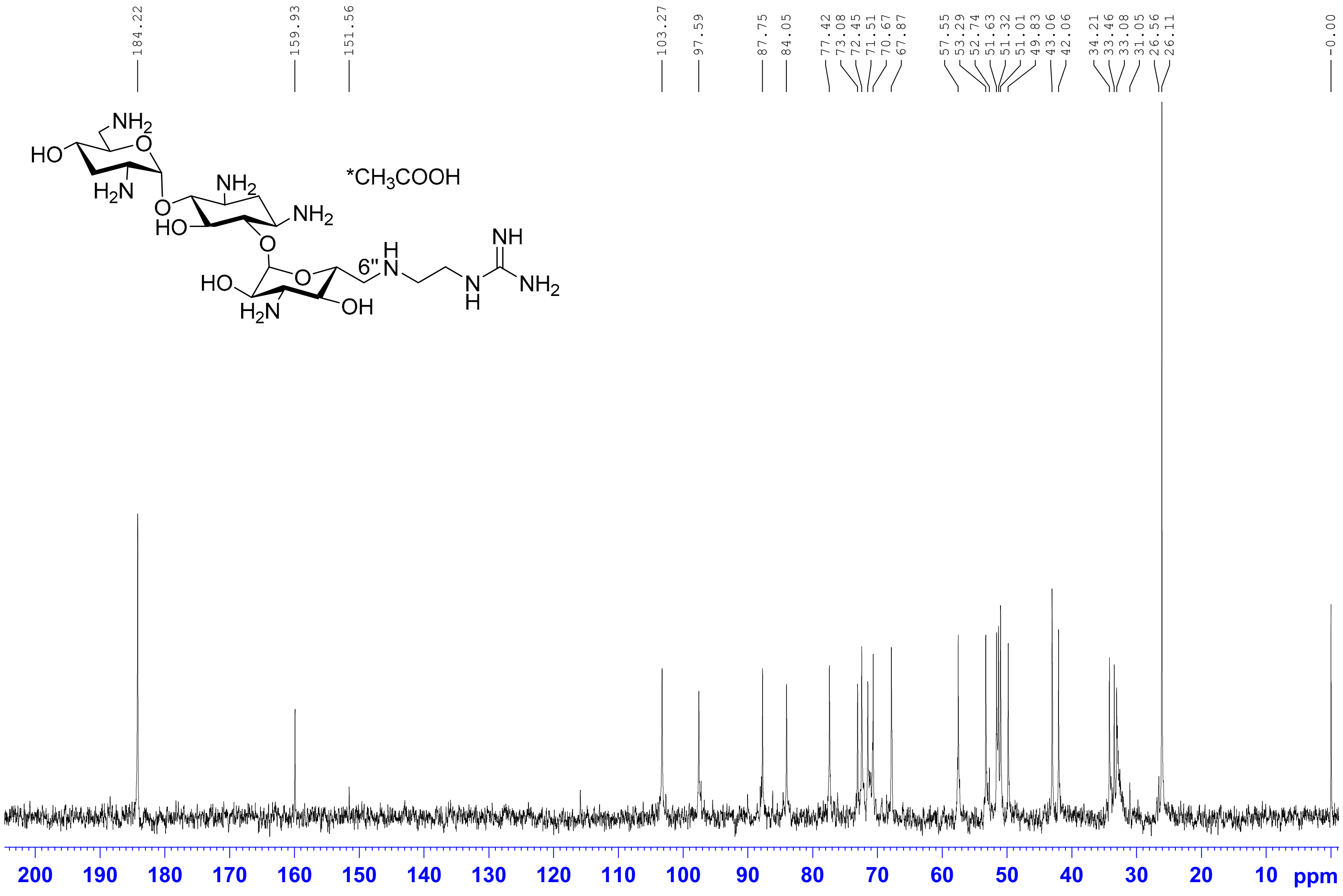

Figure S18. ^{13}C NMR (125.8 MHz, D_2O) spectrum of 6″-(2- Guanidinoethylamino)-6″- deoxytobramycin **6a**

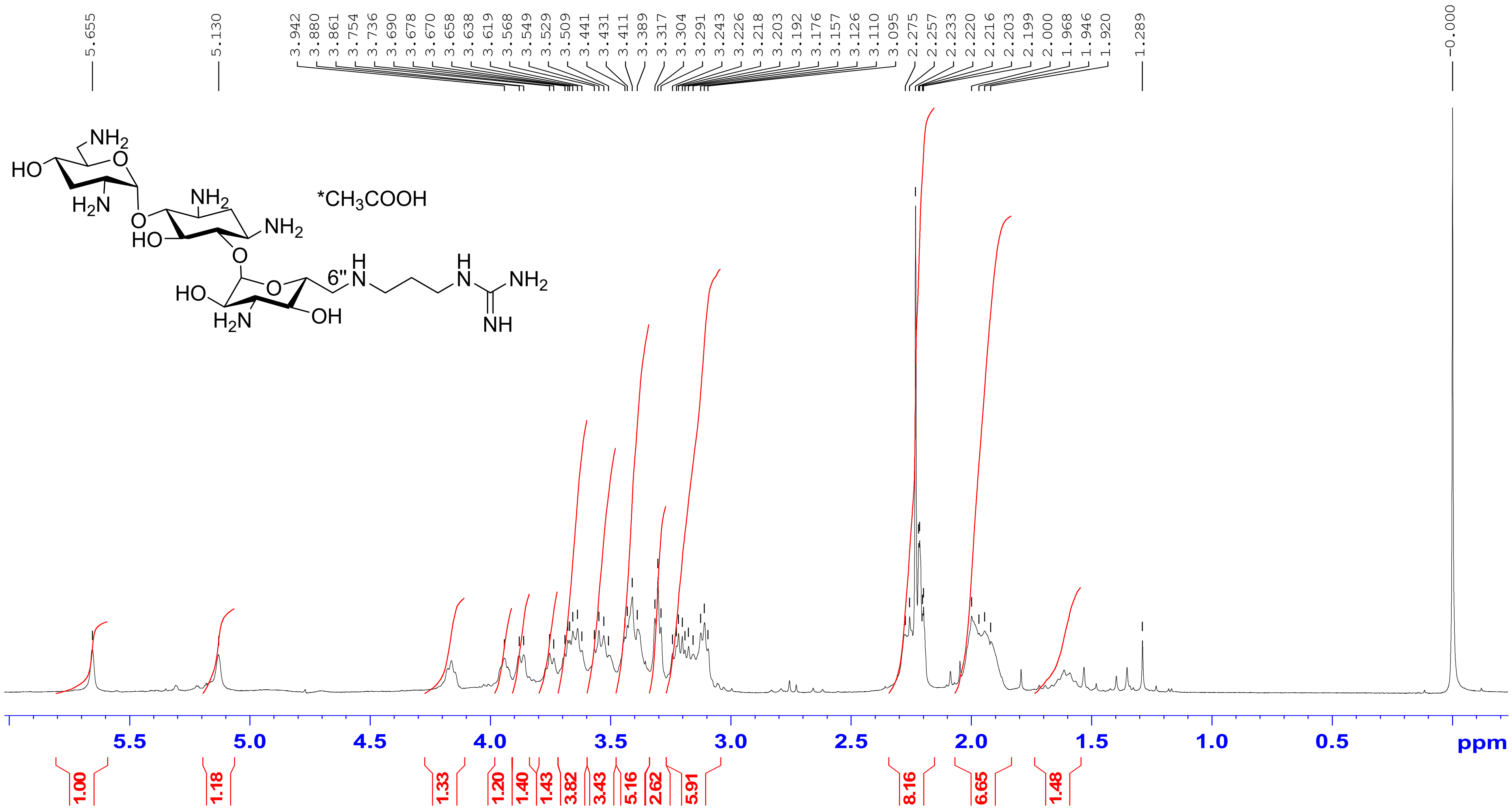

Figure S19. ^{1}H NMR (500.2 MHz, D_2O) spectrum of 6''-(3-Guanidinopropil-1-amino)-6''-deoxytobramycin **6b**

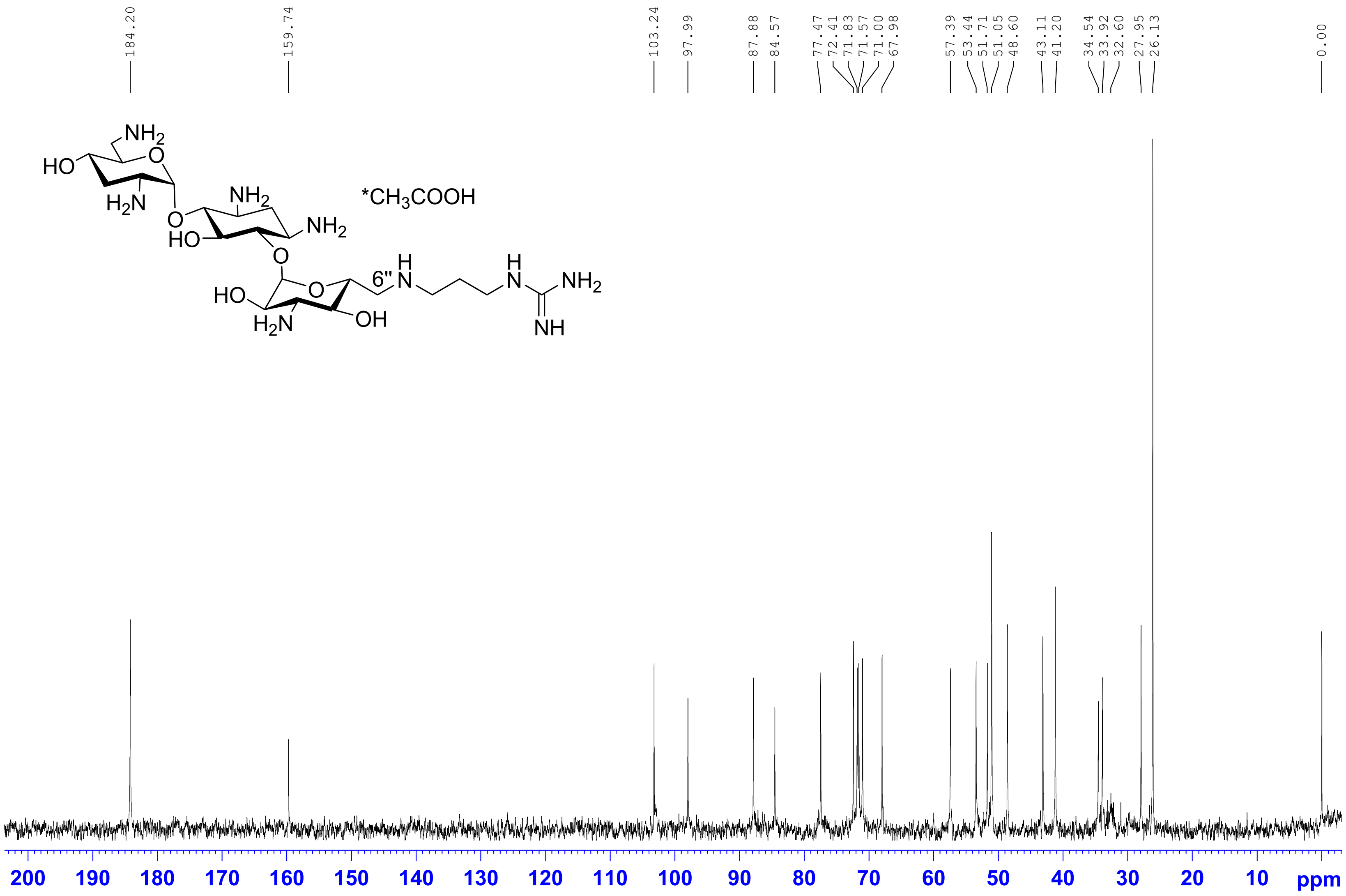

Figure S20. ^{13}C NMR (125.8 MHz, D_2O) spectrum of 6''-(3-Guanidinopropil-1-amino)-6''-deoxytobramycin **6b**

Compound Spectrum List Report

Analysis Info

Analysis Name	D:\Data\Tevjashova\SK-81-2(new).d	Acquisition Date	11/8/2024 9:46:01 PM
Method	tune_high.norm.m	Operator	Mitrokhov
Sample Name	Tune wide	Instrument / Ser#	micrOTOF-Q II 10225
Comment			

Acquisition Parameter

Source Type	ESI	Ion Polarity	Positive	Set Nebulizer	0.4 Bar
Focus	Not active	Set Capillary	4500 V	Set Dry Heater	180 °C
Scan Begin	50 m/z	Set End Plate Offset	-500 V	Set Dry Gas	4.0 l/min
Scan End	3000 m/z	Set Collision Cell RF	1600.0 Vpp	Set Divert Valve	Source

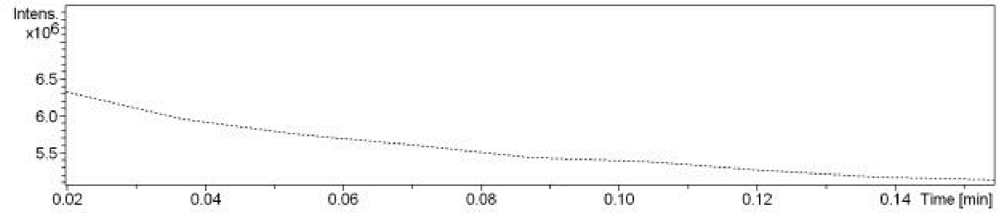

#	RT [min]	Area	Int. Type	Intens.	S/N	Chromatogram	Max. m/z
n.a.	0.1	n.a.	Average spectrum	n.a.	n.a.	n.a.	1138.4543

+MS, 0.1-0.2min #(3-9)

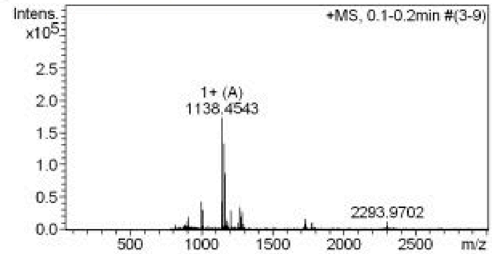

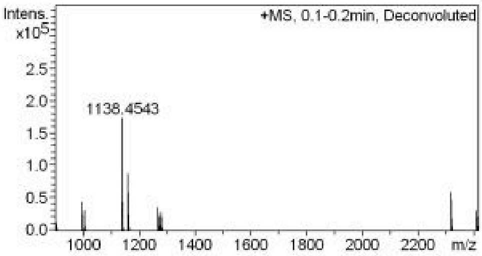

#	m/z	Res.	S/N	I	I %
1	996.6327	6534	26795.7	42108	24.4
2	1138.4543	6732	109926.7	172742	100.0
3	1139.4565	6696	70681.7	111071	64.3
4	1140.4793	6674	26891.5	42258	24.5
5	1155.5002	6854	84267.8	132421	76.7
6	1156.5043	6781	55855.0	87772	50.8
7	1157.4797	6086	36242.2	56952	33.0
8	1158.4799	6124	28931.2	45463	26.3
9	1160.4592	6594	55112.5	86605	50.1
10	1161.4605	6690	36191.2	56872	32.9

#	m/z	Res.	S/N	I	I %
1	902.8200			18725	10.8
2	996.6339			42107	24.4
3	1004.4343			29923	17.3
4	1138.4535			172742	100.0
5	1160.4577			86605	50.1
6	1266.5695			33939	19.6
7	1272.5089			19401	11.2
8	1278.5724			27083	15.7
9	2313.9306			39650	23.0
10	2404.0280			29534	17.1

Figure S21. HRMS (ESI) spectrum of 1,3,6',2′,3''-penta-*N*-Cbz-tobramycin **2a**

Compound Spectrum List Report

Analysis Info

Analysis Name	D:\Data\Tevjashova\SK-127-fr-2.d	Acquisition Date	11/17/2023 9:27:44 PM
Method	tune_high.norm.m	Operator	Mitrokhov
Sample Name	Tune wide	Instrument / Ser#	micrOTOF-Q II 10225
Comment			

Acquisition Parameter

Source Type	ESI	Ion Polarity	Positive	Set Nebulizer	0.4 Bar
Focus	Not active	Set Capillary	4500 V	Set Dry Heater	180 °C
Scan Begin	50 m/z	Set End Plate Offset	-500 V	Set Dry Gas	4.0 l/min
Scan End	3000 m/z	Set Collision Cell RF	1600.0 Vpp	Set Divert Valve	Source

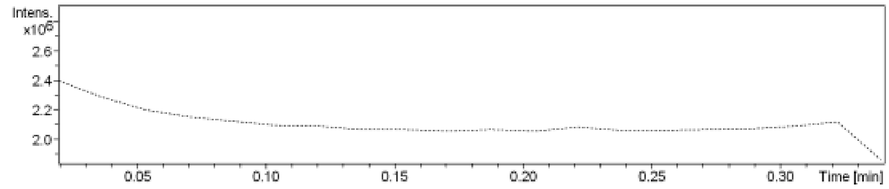

#	RT [min]	Area	Int. Type	Intens.	S/N	Chromatogram	Max. m/z
n.a.	0.1	n.a.	Average spectrum	n.a.	n.a.	n.a.	1421.5891

+MS, 0.0-0.1min #(2-7)

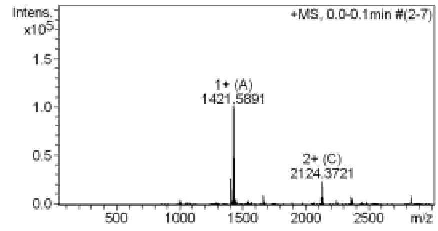

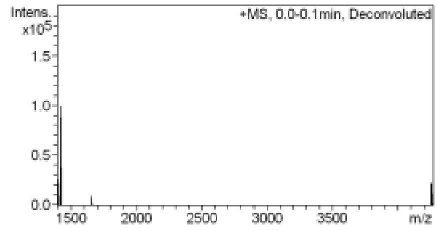

#	m/z	Res.	S/N	I	I %
1	1404.5624	9741	216.5	26941	27.1
2	1405.5670	9062	172.5	21495	21.6
3	1421.5891	9033	789.7	99309	100.0
4	1422.5905	9902	735.0	92500	93.1
5	1423.5893	10000	368.5	46449	46.8
6	1424.5773	8696	174.2	22022	22.2
7	2123.8721	11014	185.3	17095	17.2
8	2124.3721	10321	252.3	23203	23.4
9	2124.8724	9558	226.9	20876	21.0
10	2125.3718	10095	172.2	15882	16.0

#	m/z	Res.	S/N	I	I %
1	1404.5617			26941	27.1
2	1421.5848			99308	100.0
3	1425.5476			14267	14.4
4	1658.6680			10194	10.3
5	4245.7144			23202	23.4

Figure S22. HRMS (ESI) spectrum of 1,3,6′,2′,3″-penta-N-Cbz-6″-*O*-(2,4,6-triisopropylbenzosulfonyl)tobramycin **2b**

Compound Spectrum List Report

Analysis Info

Analysis Name	D:\Data\Tevjashova\SK-135.d	Acquisition Date	11/29/2023 9:05:31 PM
Method	tune_high.norm.m	Operator	Mitrokhov
Sample Name	Tune wide	Instrument / Ser#	micrOTOF-Q II 10225
Comment			

Acquisition Parameter

Source Type	ESI	Ion Polarity	Positive	Set Nebulizer	0.4 Bar
Focus	Not active	Set Capillary	4500 V	Set Dry Heater	180 °C
Scan Begin	50 m/z	Set End Plate Offset	-500 V	Set Dry Gas	4.0 l/min
Scan End	3000 m/z	Set Collision Cell RF	1600.0 Vpp	Set Divert Valve	Source

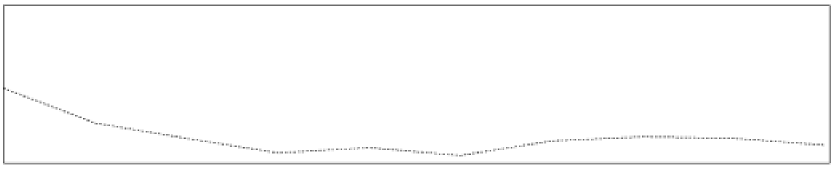

#	RT [min]	Area	Int. Type	Intens.	S/N	Chromatogram	Max. m/z
n.a.	0.1	n.a.	Average spectrum	n.a.	n.a.	n.a.	1181.5524

+MS, 0.1-0.2min #(3-9)

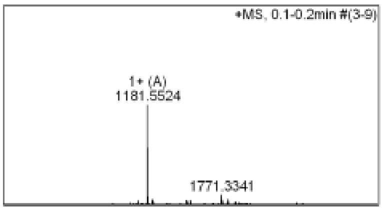

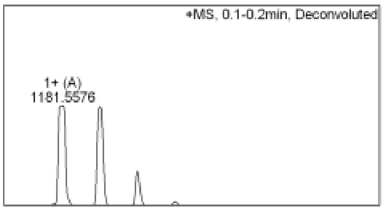

#	m/z	Res.	S/N	I	I %
1	1180.5522	5059	4116.5	1275000	100.0
2	1181.0423	9374	305.8	94900	7.4
3	1181.5524	5810	4127.0	1275000	100.0
4	1182.5662	6674	4109.4	1266274	99.3
5	1183.5646	9392	1465.5	450631	35.3
6	1224.5451	8956	247.8	68503	5.4
7	1770.8312	9085	320.5	114998	9.0
8	1771.3341	9188	338.6	121464	9.5
9	1771.8336	9235	251.3	90363	7.1
10	1820.3186	9223	166.4	60699	4.8

#	m/z	Res.	S/N	I	I %
1	1181.5576			1275000	100.0

Figure S23. HRMS (ESI) spectrum of 6''-(2-aminoethyamino)-1,3,6',2′,3''-penta-*N*-Cbz-6''-deoxytobramycin **3a**

Compound Spectrum List Report

Analysis Info

Analysis Name	D:\Data\Tevjashova\SK-139.d	Acquisition Date	12/11/2023 11:35:38 AM
Method	tune_norm.m	Operator	Mitrokhov
Sample Name	Tune wide	Instrument / Ser#	micrOTOF-Q II 10225
Comment			

Acquisition Parameter

Source Type	ESI	Ion Polarity	Positive	Set Nebulizer	0.4 Bar
Focus	Not active	Set Capillary	4500 V	Set Dry Heater	180 °C
Scan Begin	50 m/z	Set End Plate Offset	-500 V	Set Dry Gas	4.0 l/min
Scan End	3000 m/z	Set Collision Cell RF	550.0 Vpp	Set Divert Valve	Source

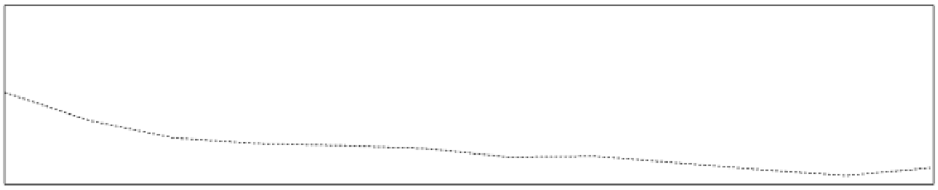

#	RT [min]	Area	Int. Type	Intens.	S/N	Chromatogram	Max. m/z
n.a.	0.1	n.a.	Average spectrum	n.a.	n.a.	n.a.	1194.5286

+MS, 0.1-0.2min #(3-10)

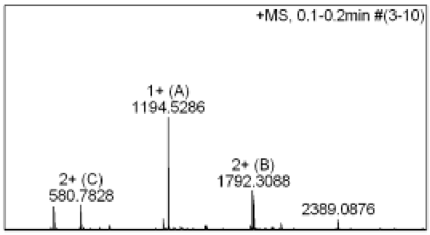

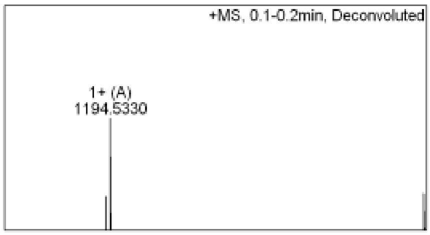

#	m/z	Res.	S/N	I	I %
1	388.1808	6027	4874.1	251894	19.8
2	580.7828	6516	3797.0	271472	21.3
3	1194.5286	5813	6112.8	1275001	100.0
4	1195.5342	6162	6133.7	1274994	100.0
5	1196.5464	7340	5139.4	1064691	83.5
6	1197.5476	8296	1265.7	261542	20.5
7	1791.3049	7639	889.1	189138	14.8
8	1791.8073	7695	1872.1	397815	31.2
9	1792.3088	7798	2048.8	435374	34.1
10	1792.8098	7722	1534.8	326297	25.6

#	m/z	Res.	S/N	I	I %
1	388.1806			251893	19.4
2	1160.5599			395721	30.5
3	1194.5330			1296919	100.0
4	3581.6018			435374	33.6

Figure S24. HRMS (ESI) spectrum of 6''-(3-aminopropyl-1-amino)-1,3,6',2′,3''-penta-*N*-Cbz-6''-deoxytobramycin **3b**

Compound Spectrum List Report

Analysis Info

Analysis Name	D:\Data\Tevjashova\SK-145.d	Acquisition Date	1/22/2024 12:36:17 PM
Method	tune_norm.m	Operator	Mitrokhov
Sample Name	Tune wide	Instrument / Ser#	micrOTOF-Q II 10225
Comment			

Acquisition Parameter

Source Type	ESI	Ion Polarity	Positive	Set Nebulizer	0.4 Bar
Focus	Not active	Set Capillary	4500 V	Set Dry Heater	180 °C
Scan Begin	50 m/z	Set End Plate Offset	-500 V	Set Dry Gas	4.0 l/min
Scan End	3000 m/z	Set Collision Cell RF	550.0 Vpp	Set Divert Valve	Source

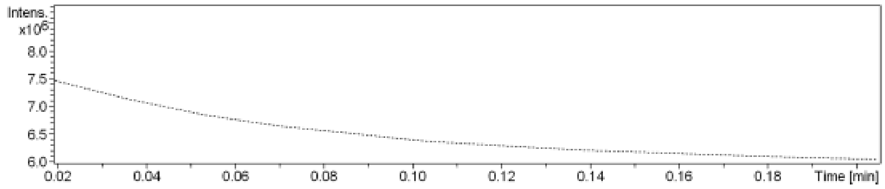

#	RT [min]	Area	Int. Type	Intens.	S/N	Chromatogram	Max. m/z
n.a.	0.1	n.a.	Average spectrum	n.a.	n.a.	n.a.	510.3222

+MS, 0.1-0.2min #(3-10)

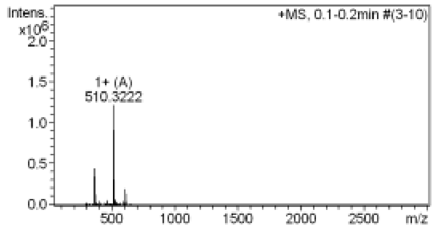

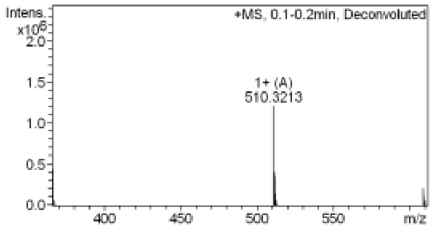

#	m/z	Res.	S/N	I	I %
1	366.2318	5959	2017.6	438464	36.2
2	367.2338	6196	327.7	71570	5.9
3	464.3030	6748	163.5	48537	4.0
4	510.3222	5357	3645.3	1212681	100.0
5	511.3228	6644	1240.7	413859	34.1
6	512.3242	6616	202.0	67709	5.6
7	524.3322	6534	195.4	67394	5.6
8	590.3812	6886	132.8	40673	3.4
9	608.3915	6629	655.6	188634	15.6
10	609.3947	6703	201.3	57858	4.8

#	m/z	Res.	S/N	I	I %
1	366.2316			438464	36.2
2	510.3213			1212680	100.0
3	608.3915			211206	17.4

Figure S25. HRMS (ESI) spectrum of 6''-(2-aminoethyamino)-6''-deoxytobramycin **4a**

Compound Spectrum List Report

Analysis Info

Analysis Name	D:\Data\Tevjashova\SK-142.d	Acquisition Date	12/16/2023 4:21:58 PM
Method	tune_norm.m	Operator	Mitrokhov
Sample Name	Tune wide	Instrument / Ser#	micrOTOF-Q II 10225
Comment			

Acquisition Parameter

Source Type	ESI	Ion Polarity	Positive	Set Nebulizer	0.4 Bar
Focus	Not active	Set Capillary	4500 V	Set Dry Heater	180 °C
Scan Begin	50 m/z	Set End Plate Offset	-500 V	Set Dry Gas	4.0 l/min
Scan End	3000 m/z	Set Collision Cell RF	550.0 Vpp	Set Divert Valve	Source

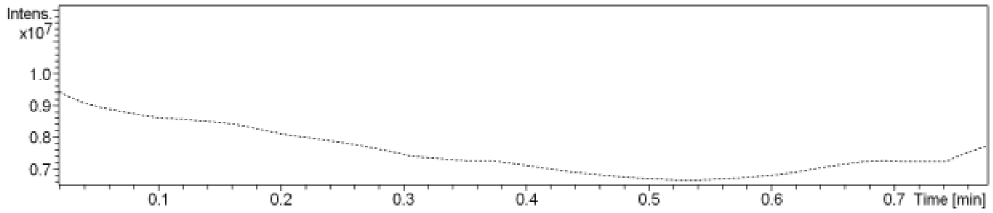

#	RT [min]	Area	Int. Type	Intens.	S/N	Chromatogram	Max. m/z
n.a.	0.2	n.a.	Average spectrum	n.a.	n.a.	n.a.	524.3519

+MS, 0.1-0.3min #(3-18)

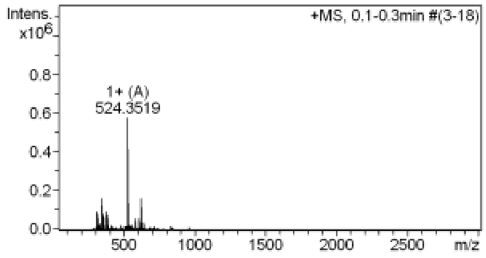

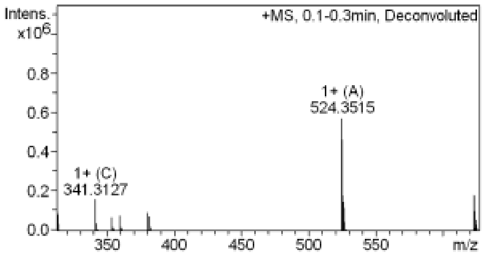

#	m/z	Res.	S/N	I	I %
1	313.2818	6013	484.4	91847	15.9
2	341.3129	6183	722.0	158790	27.5
3	353.2739	6407	282.6	63781	11.1
4	359.3237	6348	339.6	77578	13.4
5	380.2589	6228	379.0	90202	15.6
6	381.3017	5998	304.1	72557	12.6
7	524.3519	6472	1899.7	576915	100.0
8	525.3538	6646	476.4	145168	25.2
9	580.3781	6986	191.5	55524	9.6
10	622.4263	6998	608.5	161950	28.1

#	m/z	Res.	S/N	I	I %
1	313.2817			91847	15.9
2	341.3127			158790	27.5
3	353.2746			63781	11.1
4	359.3234			77577	13.4
5	380.2785			90202	15.6
6	524.3515			576915	100.0
7	622.4255			178028	30.9

Figure S26. HRMS (ESI) spectrum of 6″-(3-aminopropyl-1-amino)- 6″-deoxytobramycin **4b**

Compound Spectrum List Report

Analysis Info

Analysis Name	D:\Data\Tevjashova\SK-151.d	Acquisition Date	2/11/2024 5:47:21 PM
Method	tune_norm.m	Operator	Mitrokhov
Sample Name	Tune wide	Instrument / Ser#	micrOTOF-Q II 10225
Comment			

Acquisition Parameter

Source Type	ESI	Ion Polarity	Positive	Set Nebulizer	0.4 Bar
Focus	Not active	Set Capillary	4500 V	Set Dry Heater	180 °C
Scan Begin	50 m/z	Set End Plate Offset	-500 V	Set Dry Gas	4.0 l/min
Scan End	3000 m/z	Set Collision Cell RF	550.0 Vpp	Set Divert Valve	Source

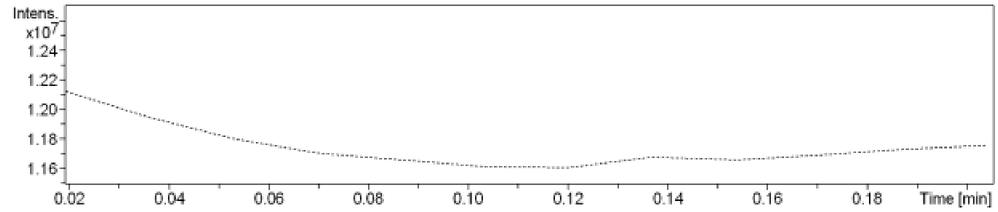

#	RT [min]	Area	Int. Type	Intens.	S/N	Chromatogram	Max. m/z
n.a.	0.1	n.a.	Average spectrum	n.a.	n.a.	n.a.	1222.5284

+MS, 0.1-0.2min #(3-10)

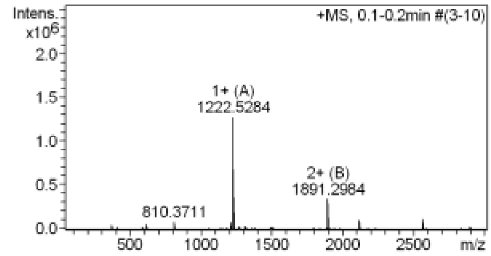

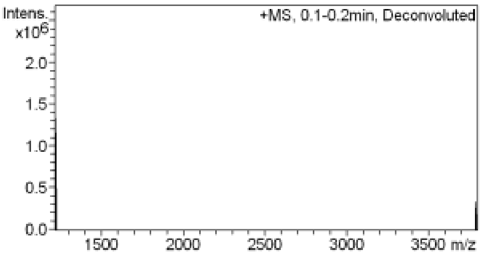

#	m/z	Res.	S/N	I	I %
1	1222.5284	5605	7139.8	1275001	100.0
2	1223.5275	6325	7165.1	1275000	100.0
3	1224.5440	7118	6820.0	1209214	94.8
4	1225.5430	9073	2028.6	358628	28.1
5	1890.2952	8285	556.3	142061	11.1
6	1890.7968	8281	1195.8	305360	23.9
7	1891.2984	8483	1348.4	344943	27.1
8	1891.7992	8488	1035.3	265491	20.8
9	1892.3008	8439	596.1	153383	12.0
10	2113.8712	8053	606.3	103854	8.1

#	m/z	Res.	S/N	I	I %
1	1222.5301			1335191	100.0
2	3779.5815			344942	25.8

Figure S27. HRMS (ESI) spectrum of 1,3,6',2',3''-penta-N-Cbz-6''-(2-guanidinoethylamino)-6''-deoxytobramycin **5a**

Compound Spectrum List Report

Analysis Info

Analysis Name	D:\Data\Tevjashova\SK-154().d	Acquisition Date	3/14/2024 6:34:03 PM
Method	tune_norm.m	Operator	Mitrokhov
Sample Name	Tune wide	Instrument / Ser#	micrOTOF-Q II 10225
Comment			

Acquisition Parameter

Source Type	ESI	Ion Polarity	Positive	Set Nebulizer	0.4 Bar
Focus	Not active	Set Capillary	4500 V	Set Dry Heater	180 °C
Scan Begin	50 m/z	Set End Plate Offset	-500 V	Set Dry Gas	4.0 l/min
Scan End	3000 m/z	Set Collision Cell RF	550.0 Vpp	Set Divert Valve	Source

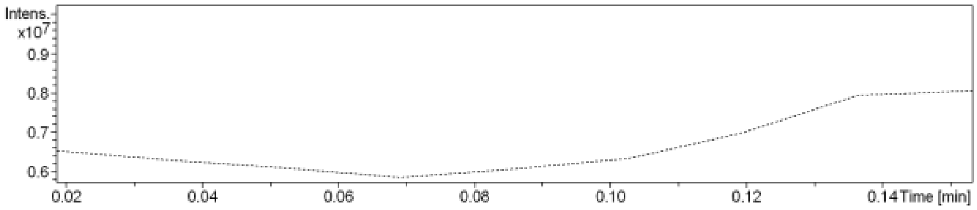

#	RT [min]	Area	Int. Type	Intens.	S/N	Chromatogram	Max. m/z
n.a.	0.1	n.a.	Average spectrum	n.a.	n.a.	n.a.	1236.5483

+MS, 0.0-0.1min #(2-6)

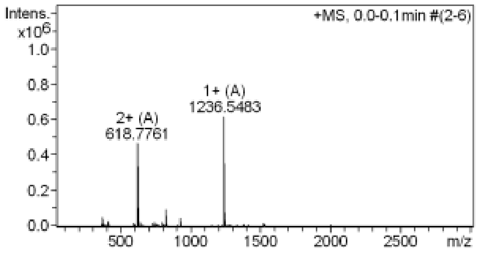

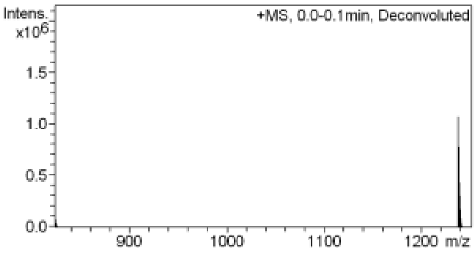

#	m/z	Res.	S/N	I	I %
1	369.1811	6149	1779.8	47229	7.6
2	618.7761	6634	5805.9	466723	75.5
3	619.2782	6697	4177.7	336491	54.4
4	619.7793	6755	1674.8	135208	21.9
5	824.3849	7429	841.2	89252	14.4
6	923.5131	7636	480.6	45880	7.4
7	1236.5483	7561	4910.1	618560	100.0
8	1237.5506	7840	3523.3	445900	72.1
9	1238.5526	7916	1323.5	168374	27.2
10	1239.5541	8107	344.5	44182	7.1

#	m/z	Res.	S/N	I	I %
1	824.3859			89252	8.2
2	1236.5463			1085281	100.0

Figure S28. HRMS (ESI) spectrum of 1,3,6',2′,3"-penta-N-Cbz-6″-(3-guanidinpropil-1-amino)-6"-deoxytobramycin **5b**

Compound Spectrum List Report

Analysis Info

Analysis Name	D:\Data\Tevjashova\SK-157().d	Acquisition Date	3/23/2024 12:39:47 PM
Method	tune_norm.m	Operator	Mitrokhov
Sample Name	Tune wide	Instrument / Ser#	micrOTOF-Q II 10225
Comment			

Acquisition Parameter

Source Type	ESI	Ion Polarity	Positive	Set Nebulizer	0.8 Bar
Focus	Not active	Set Capillary	4500 V	Set Dry Heater	180 °C
Scan Begin	50 m/z	Set End Plate Offset	-500 V	Set Dry Gas	4.0 l/min
Scan End	3000 m/z	Set Collision Cell RF	550.0 Vpp	Set Divert Valve	Source

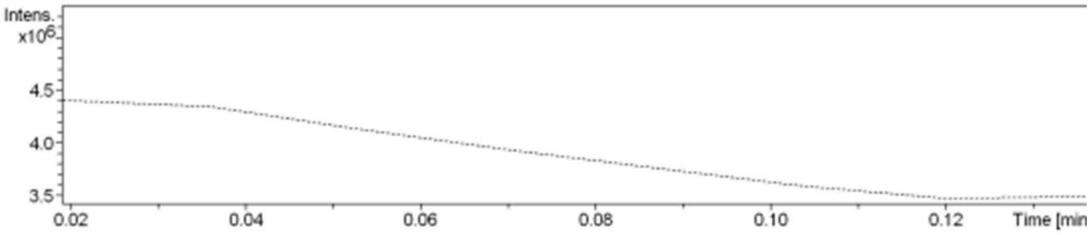

#	RT [min]	Area	Int. Type	Intens.	S/N	Chromatogram	Max. m/z
n.a.	0.1	n.a.	Average spectrum	n.a.	n.a.	n.a.	552.3418

+MS, 0.0-0.1min #(2-6)

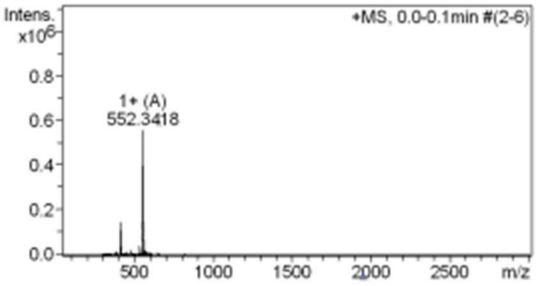

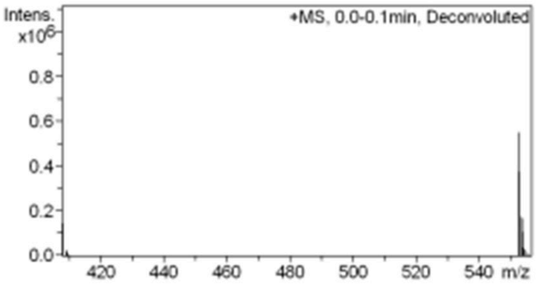

#	m/z	Res.	S/N	I	I %
1	391.2096	6890	71.3	13458	2.4
2	408.2370	6262	717.4	145264	26.1
3	409.2393	6379	138.4	28245	5.1
4	481.2390	6875	73.2	19700	3.5
5	535.2949	6800	119.5	37806	6.8
6	552.3418	6152	1827.1	557155	100.0
7	553.3228	6990	562.2	171100	30.7
8	554.3246	6695	105.0	32045	5.8
9	568.3144	7252	70.1	20622	3.7
10	580.3142	6971	57.3	16321	2.9

#	m/z	Res.	S/N	I	I %
1	408.2361			145263	26.1
2	552.3411			557155	100.0

Figure S29. HRMS (ESI) spectrum of 6″-(2- Guanidinoethylamino)-6″-deoxytobramycin **6a**

Compound Spectrum List Report

Analysis Info

Analysis Name	D:\Data\Tevjashova\SK-156.d	Acquisition Date	3/23/2024 1:35:23 PM
Method	tune_norm.m	Operator	Mitrokhov
Sample Name	Tune wide	Instrument / Ser#	micrOTOF-Q II 10225
Comment			

Acquisition Parameter

Source Type	ESI	Ion Polarity	Positive	Set Nebulizer	0.4 Bar
Focus	Not active	Set Capillary	4500 V	Set Dry Heater	180 °C
Scan Begin	50 m/z	Set End Plate Offset	-500 V	Set Dry Gas	4.0 l/min
Scan End	3000 m/z	Set Collision Cell RF	550.0 Vpp	Set Divert Valve	Source

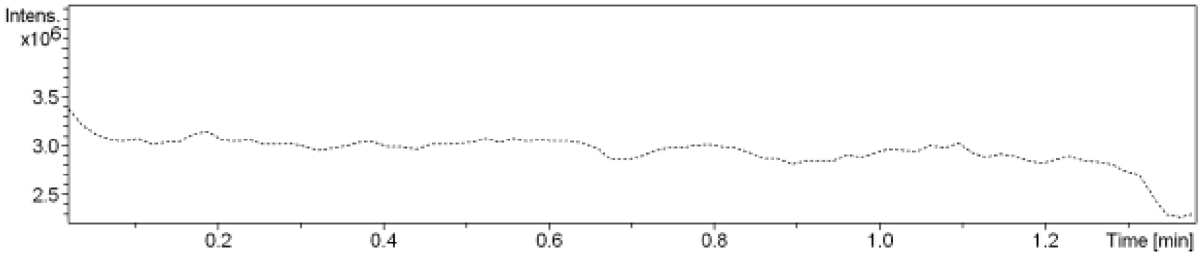

#	RT [min]	Area	Int. Type	Intens.	S/N	Chromatogram	Max. m/z
n.a.	0.6	n.a.	Average spectrum	n.a.	n.a.	n.a.	566.3572

+MS, 0.2-1.1min #(9-68)

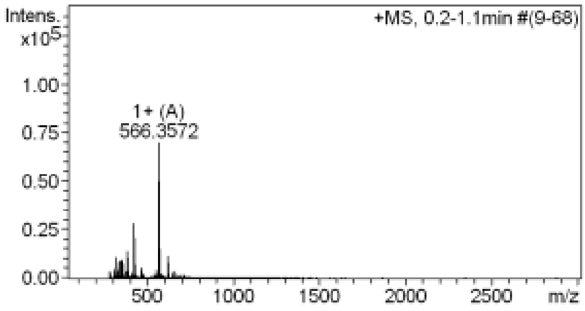

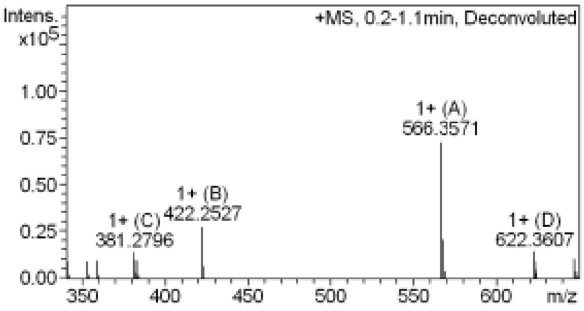

#	m/z	Res.	S/N	I	I %
1	323.7022	6233	114.1	11013	15.8
2	341.2892	6151	87.6	9138	13.1
3	353.2501	6375	79.8	8701	12.5
4	359.2993	6149	85.8	9563	13.7
5	381.2796	6304	119.0	14255	20.4
6	383.1871	6127	81.4	9830	14.1
7	422.2528	6372	210.6	28550	40.9
8	566.3572	6730	400.0	69824	100.0
9	567.3397	7150	116.0	20354	29.2
10	622.3607	6985	71.0	11746	16.8

#	m/z	Res.	S/N	I	I %
1	341.2890			9138	12.4
2	353.2500			8700	11.8
3	359.2992			9562	13.0
4	381.2796			14254	19.3
5	383.1874			9830	13.3
6	422.2527			28549	38.7
7	566.3571			73768	100.0
8	622.3607			14752	20.0
9	646.3967			11012	14.9

Figure S30. HRMS (ESI) spectrum of 6''-(3-Guanidinopropil-1-amino)-6''-deoxytobramycin **6b**

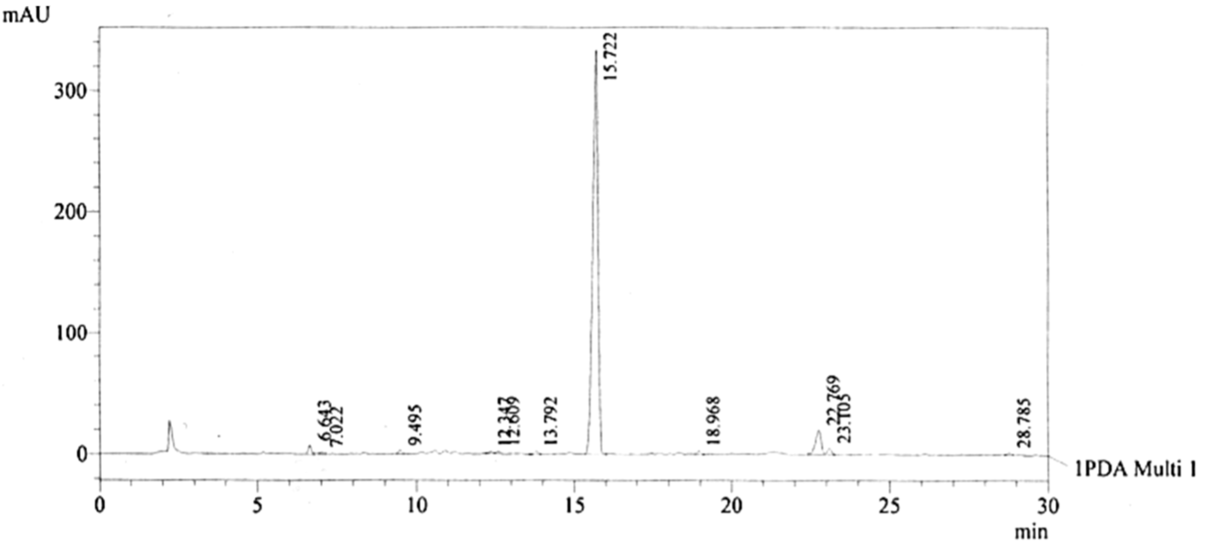

1 PDA Multi 1 / 254nm 4nm

PeakTable

PDA Ch1 254nm 4nm

Peak#	Ret. Time	Area	Height	Area %
1	6.643	43589	6831	0.998
2	7.022	10458	1350	0.240
3	9.495	21338	2731	0.489
4	12.347	9842	1225	0.225
5	12.609	13409	1790	0.307
6	13.792	17973	2124	0.412
7	15.722	3938145	333194	90.196
8	18.968	18598	2325	0.426
9	22.769	235890	19651	5.403
10	23.105	40673	5056	0.932
11	28.785	16316	2042	0.374
Total		4366230	378319	100.000

Figure S31. HPLC chromatogram of 1,3,6',2′,3''-penta-*N*-Cbz-tobramycin **2a**

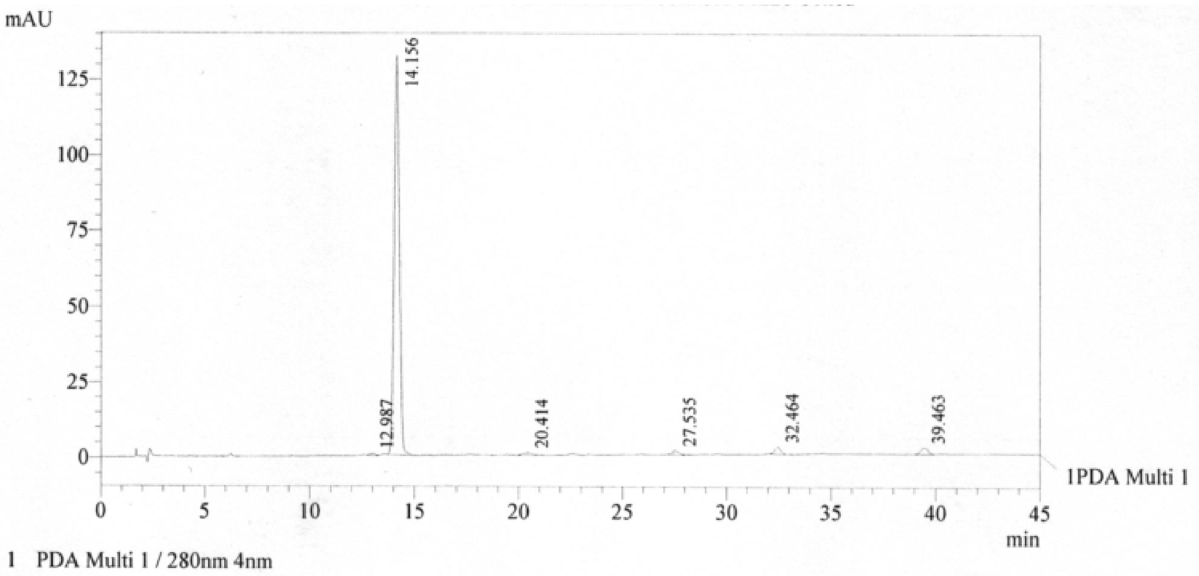

PeakTable

PDA Ch1 280nm 4nm

Peak#	Ret. Time	Area	Height	Area %
1	12.987	8690	577	0.357
2	14.156	2301841	131834	94.452
3	20.414	17617	689	0.723
4	27.535	22373	1133	0.918
5	32.464	40238	2228	1.651
6	39.463	46300	1949	1.900
Total		2437060	138410	100.000

Figure S32. HPLC chromatogram of 1,3,6′,2′,3″-penta-N-Cbz-6″-*O*-(2,4,6-triisopropylbenzosulfonyl)tobramycin **2b**

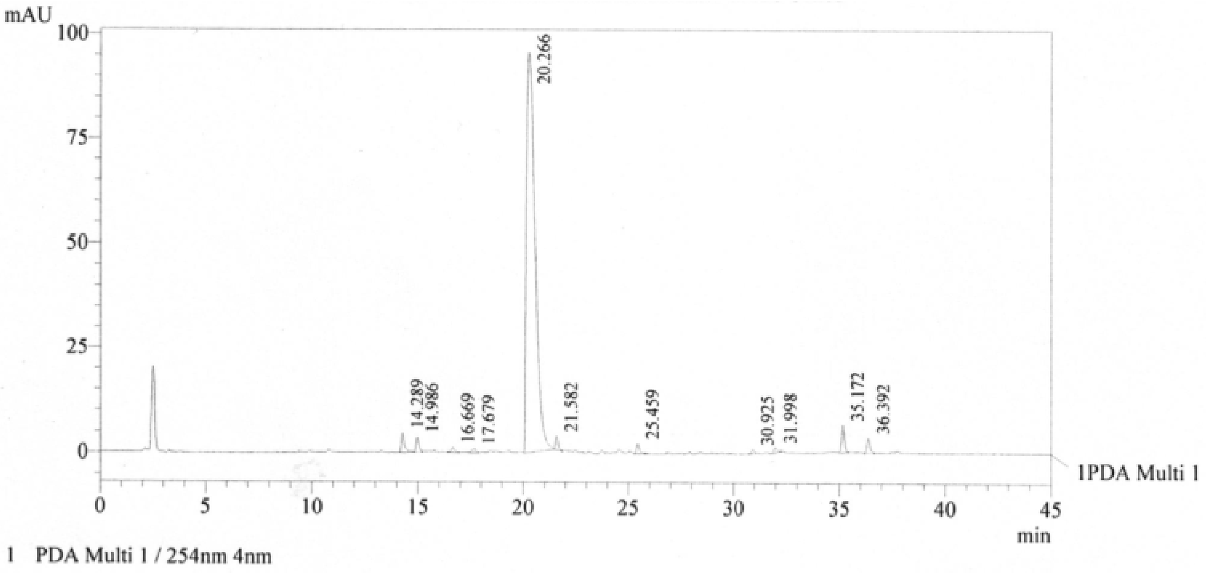

PeakTable

PDA Ch1 254nm 4nm

Peak#	Ret. Time	Area	Height	Area %
1	14.289	44720	4492	1.517
2	14.986	29556	3411	1.003
3	16.669	12161	1053	0.413
4	17.679	11204	788	0.380
5	20.266	2692931	95605	91.368
6	21.582	25494	3177	0.865
7	25.459	20353	2239	0.691
8	30.925	7737	826	0.263
9	31.998	10924	1112	0.371
10	35.172	58823	6340	1.996
11	36.392	33433	3428	1.134
Total		2947337	122472	100.000

Figure S33. HPLC chromatogram of 6''-(2-aminoethyamino)-1,3,6',2′,3''-penta-*N*-Cbz-6''-deoxytobramycin **3a**

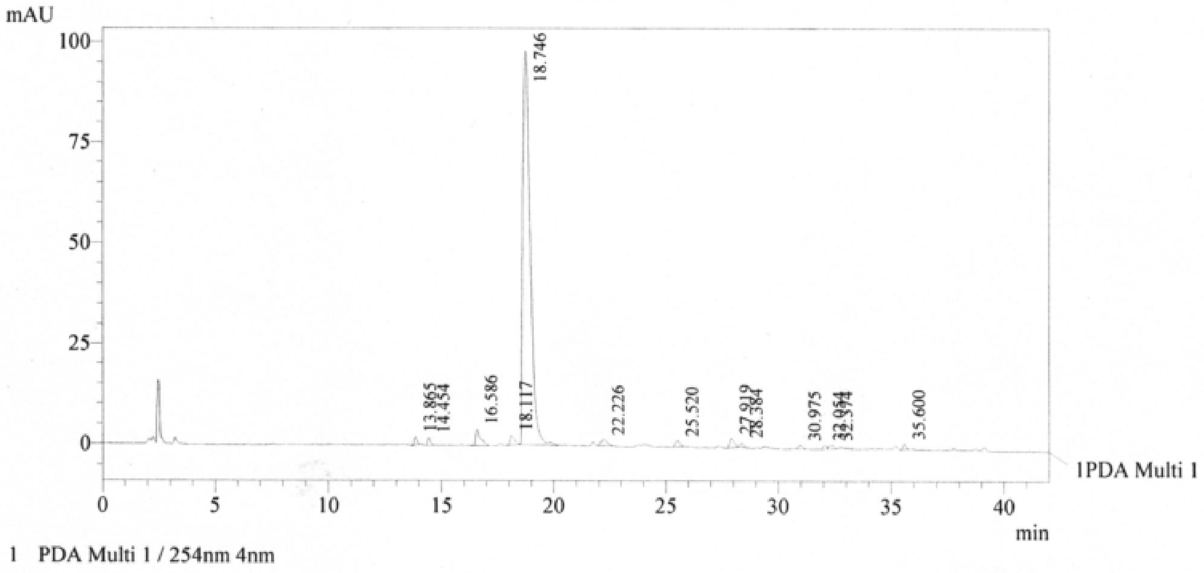

PeakTable

PDA Ch1 254nm 4nm

Peak#	Ret. Time	Area	Height	Area %
1	13.865	15550	1939	0.630
2	14.454	14419	1775	0.584
3	16.586	55854	3953	2.263
4	18.117	30197	2250	1.224
5	18.746	2230503	97836	90.388
6	22.226	24800	1428	1.005
7	25.520	18173	1384	0.736
8	27.919	30461	2198	1.234
9	28.384	9025	1078	0.366
10	30.975	8441	821	0.342
11	32.054	8590	848	0.348
12	32.374	11647	860	0.472
13	35.600	10046	1260	0.407
Total		2467705	117631	100.000

Figure S34. HPLC chromatogram of 6''-(3-aminopropyl-1-amino)-1,3,6',2′,3''-penta-*N*-Cbz-6''-deoxytobramycin **3b**

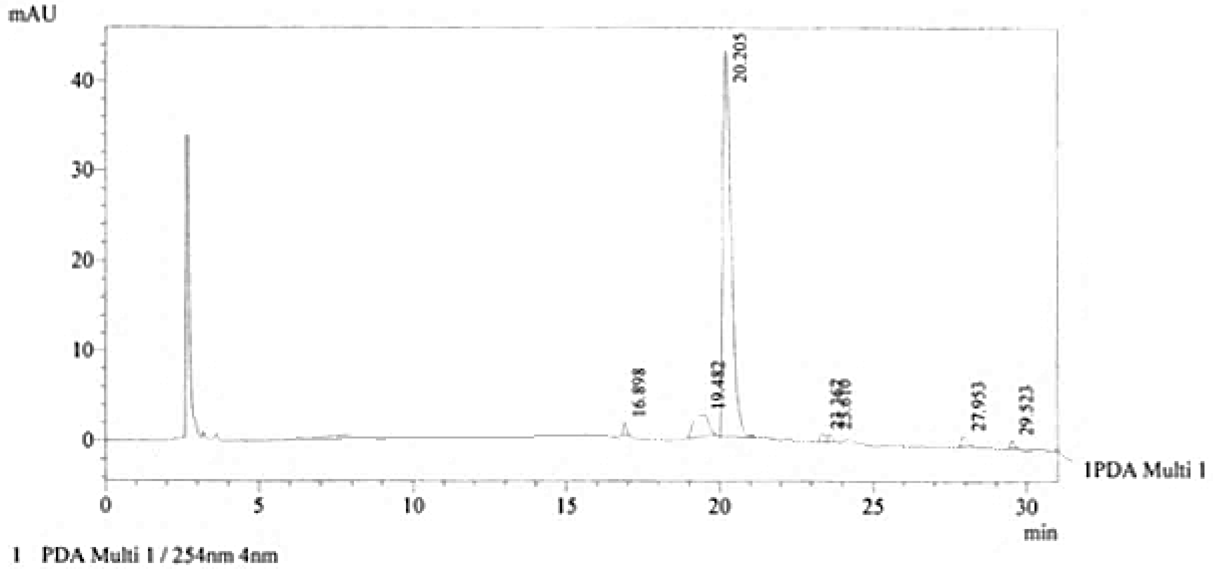

PeakTable

PDA Ch1 254nm 4nm

Peak#	Ret. Time	Area	Height	Area %
1	16.898	10894	1361	1.153
2	19.482	79660	2352	8.429
3	20.205	812912	42912	86.019
4	23.367	11074	879	1.172
5	23.610	7380	862	0.781
6	27.953	14817	1175	1.568
7	29.523	8304	922	0.879
Total		945041	50462	100.000

Figure S35. HPLC chromatogram of 1,3,6',2′,3''-penta-N-Cbz-6″-(2-guanidinoethylamino)-6''-deoxytobramycin **5a**

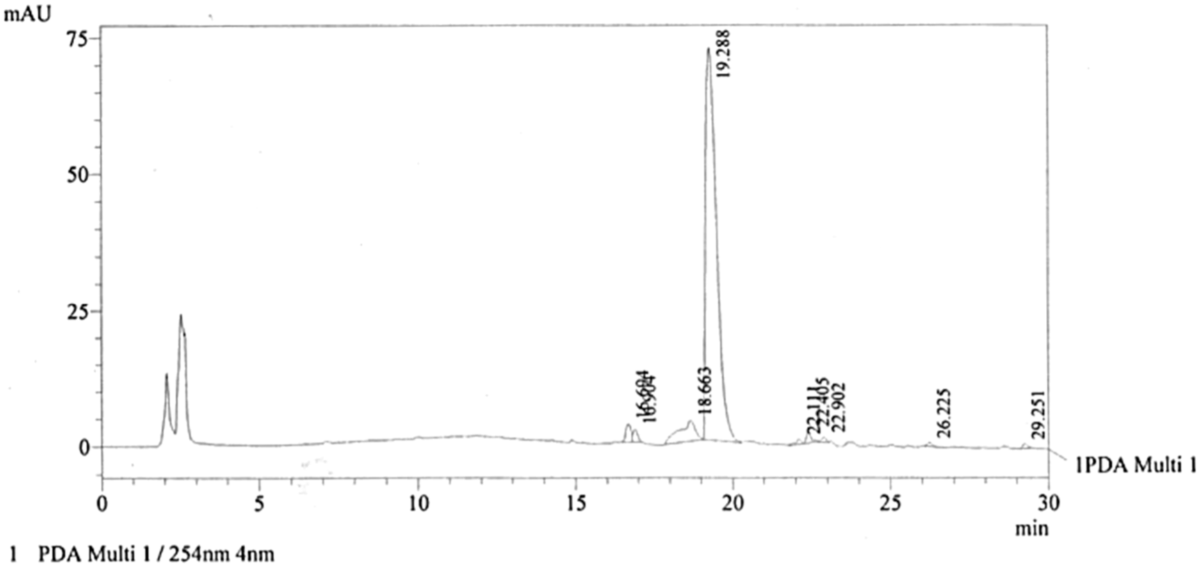

PeakTable

PDA Ch1 254nm 4nm

Peak#	Ret. Time	Area	Height	Area %
1	16.694	35512	3202	1.817
2	16.904	21322	2252	1.091
3	18.663	130317	3697	6.669
4	19.288	1716075	71932	87.826
5	22.111	9275	892	0.475
6	22.405	20644	2171	1.057
7	22.902	7550	849	0.386
8	26.225	6061	625	0.310
9	29.251	7197	762	0.368
Total		1953954	86384	100.000

Figure S36. HPLC chromatogram of 1,3,6',2′,3''-penta-N-Cbz-6″-(3-guanidinpropil-1-amino)-6''-deoxytobramycin **5b**

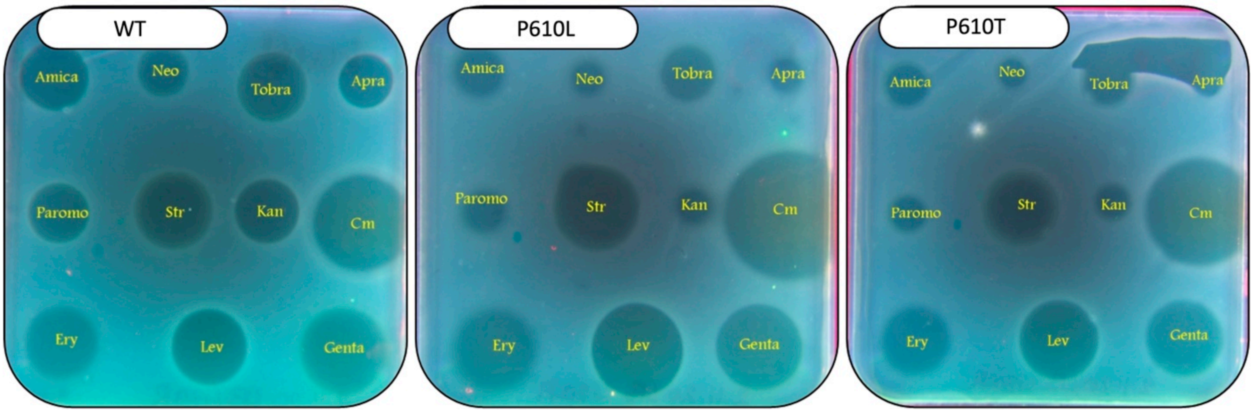

Figure S37. Drop-test for *E. coli* strains with P610T/L substitution in EF-G. Mutants are shown to have some resistance to the antibiotics of the 2-deoxystreptamine group. Antibiotics: amikacin, neomycin, tobramycin, apramycin, paromomycin, streptomycin, kanamycin A, chloramphenicol, erythromycin, levofloxacin, gentamicin.

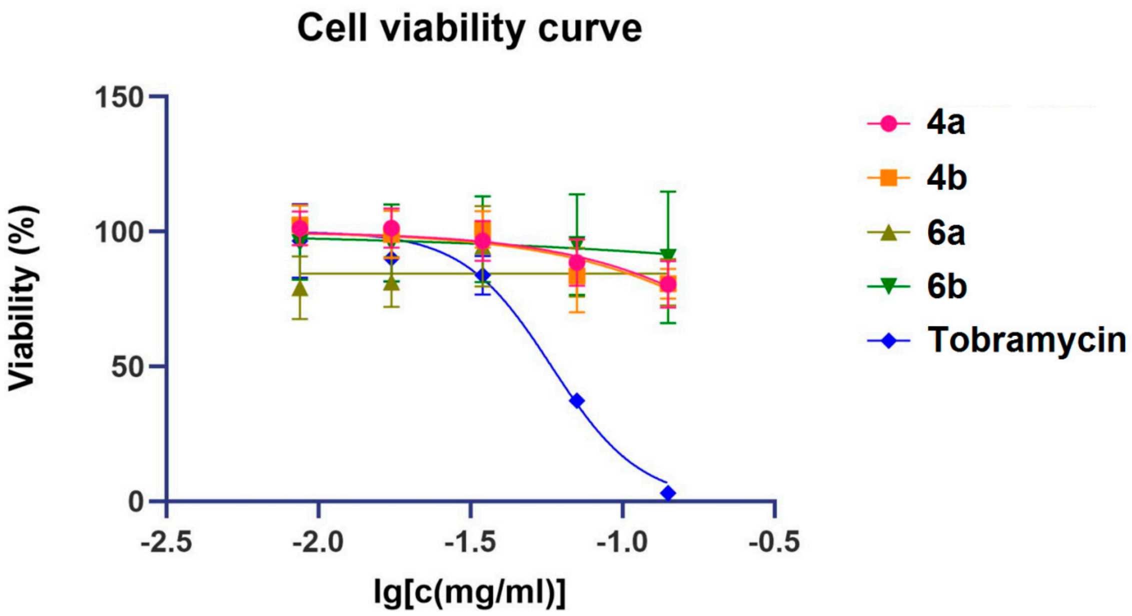

Figure S38. Cell viability curves of HEK293T cell line incubated with tested substances. IC50abs values were calculated by approximating the cells percent of viability using the four parameters nonlinear regression function.
